# Strong and localized recurrence controls the dimensionality of neural activity across brain areas

**DOI:** 10.1038/s41593-026-02395-w

**Published:** 2026-08-19

**Authors:** David Dahmen, Stefano Recanatesi, Xiaoxuan Jia, Gabriel K. Ocker, Nilufar Lahiji, Simon Musall, Luke Campagnola, Stephanie Seeman, Tim Jarsky, Moritz Helias, Eric Shea-Brown

**Affiliations:** 1 Institute for Advanced Simulation (IAS-6), Jülich Research Centre, Jülich, Germany; 2 University of Washington Computational Neuroscience Center, Department of Applied Mathematics, and Swartz Center for Theoretical Neuroscience, Seattle, WA USA; 3 Ruth and Bruce Rappaport Faculty of Medicine, Technion-Israel Institute of Technology, Haifa, Israel; 4 School of Life Sciences and IDG/McGovern Institute for Brain Research, Tsinghua University, Beijing, China; 5 Allen Institute, Seattle, WA USA; 6 Department of Mathematics and Statistics, Boston University, Boston, MA USA; 7 Systems Neurophysiology, Institute of Zoology, RWTH Aachen University, Aachen, Germany; 8 Bioelectronics (IBI-3), Jülich Research Centre, Jülich, Germany; 9 Institute of Experimental Epileptology and Cognition Research, Medical Faculty, University of Bonn, Bonn, Germany; 10 Department of Physics, Faculty 1, RWTH Aachen University, Aachen, Germany

**Keywords:** Network models, Neural circuits, Visual system

## Abstract

The brain contains an astronomical number of neurons, but it is their collective activity that underlies brain function. The number of degrees of freedom that this activity explores (its dimensionality) is therefore a fundamental signature of neural dynamics. However, it is not known what controls dimensionality in the biological brain. Through analysis of high-density Neuropixels recordings, here, we argue that areas across the mouse cortex predominantly operate in a sensitive regime that gives recurrent synaptic networks a strong role in regulating dimensionality. This control is expressed across time, as cortical activity transitions among states with different dimensionalities. Moreover, this control is mediated through highly tractable features of synaptic networks (network motifs). Analyzing a massive synaptic physiology dataset, we find that motifs impacting dimensionality are prevalent in both mouse and human brains. Thus, local circuitry scales up systematically to help control the degrees of freedom that brain networks may explore and exploit.

## Abstract (web summary)

Brain activity is complex but not arbitrary, and some patterns dominate others, with dimensionality describing how many such patterns exist. The authors show how brain connectivity controls dimensionality, allowing complexity to vary across time and behavior.

## Main

The complexity of a neural network’s activity can be measured by its dimensionality, that is, the number of collective degrees of freedom that its neurons explore. Dimensionality is closely linked to neural computation. Signal classification, for example, benefits from network activities that increase the dimensionality of the incoming signals to be classified^1–5^. However, compressing inputs into lower-dimensional activity patterns helps generalization to novel signals^6–8^. Studies have emphasized the comparatively high^5,9,10^ or low ^11,12^ dimensionality of recordings in various experimental settings. Moreover, the dimensionality of neural dynamics can change over time^13^, throughout the information processing hierarchy^14^ or during learning^7,15,16^. These findings, taken together, highlight the importance of dimensionality as a property of network activity that will vary depending on the type of computation performed in a circuit. A key question is: how can the connectivity of a network regulate the dimensionality of its activity^17–21^?

This question is of particular interest for cortical networks that typically exhibit quite asynchronous activity^22–24^; this asynchrony at first seems to imply almost independent dynamics that are roughly as high-dimensional as the number of neurons in the network. However, by analyzing electrophysiological recordings from over 8,000 neurons^25,26^, we show that this is not the case; as quantified by several popular dimensionality metrics, dynamics are constrained to spaces of much lower dimension than the number of neurons in local brain circuits. This results from the accumulation of many weak but diverse pairwise correlations across the networks (see also ref. ^27^), as quantified by the variance of these correlations, over and above their average.

To understand the mechanistic origins of this rather low relative dimensionality of cortical activity, we show how the corresponding high variance of correlations results from the network’s strong recurrent connectivity. Moreover, we show that in this strongly recurrent regime, dimensionality is highly sensitive to changes in recurrent connectivity. Beyond overall synaptic strength, specific connectivity patterns (or motifs) between pairs and triplets of cells ^21,28–36^ can also tune the dimensionality of neural activity. We analyze newly released synaptic physiology datasets^37^, quantifying connections among more than 32,000 pairs of neurons, and find that the connectivity motifs implicated by our theory are strongly present in both mouse and human brains. Moreover, connectivity strength and motifs depend on the activity level of cell types engaged in a circuit at a given time, providing a mechanism by which dimensionality can change across cortical states in line with marked dimensionality transitions we observe in the cortex.

## Results

### Highly constrained dimensionality of neural states

**Fig. 1:**
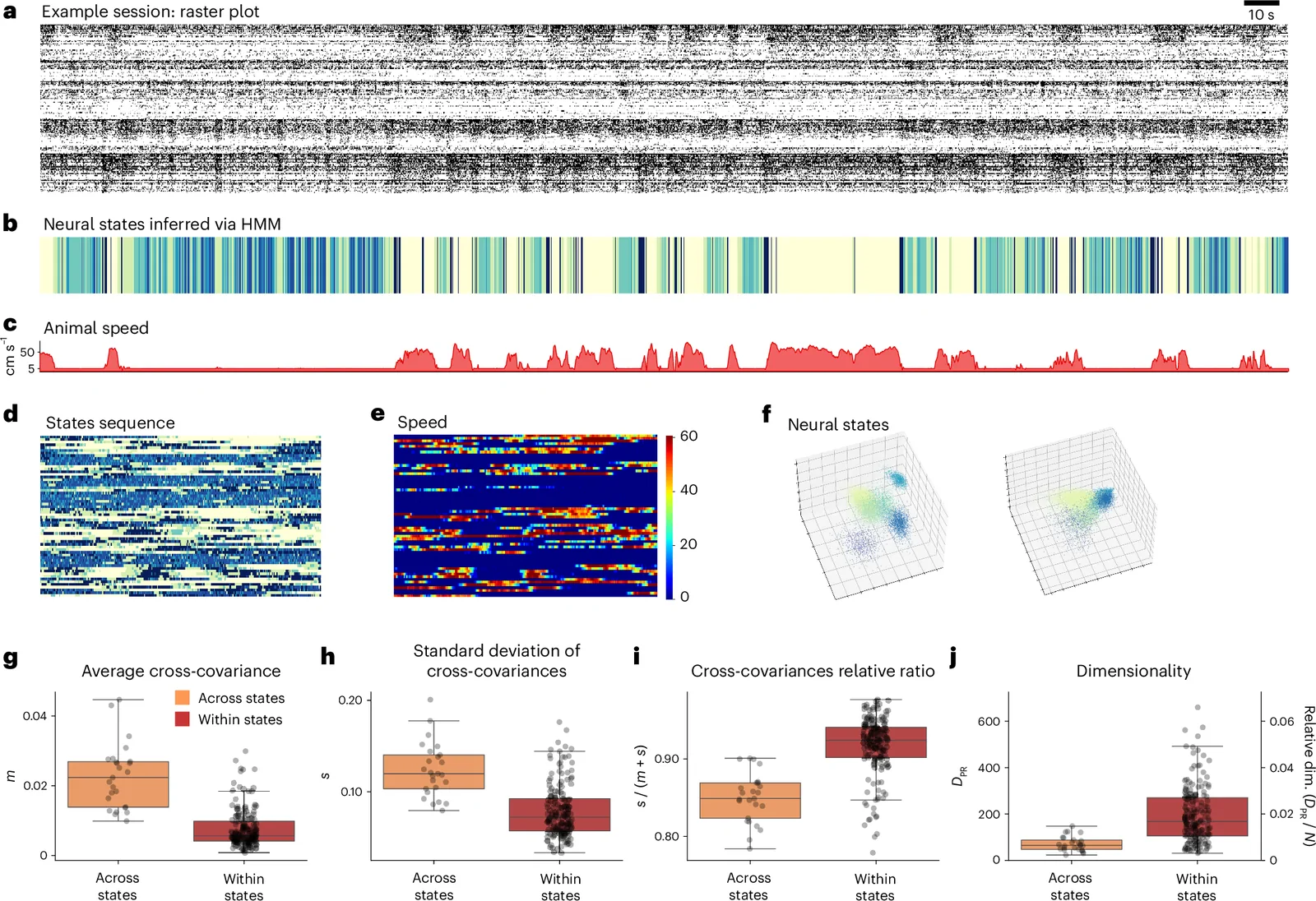
Dimensionality estimation in neural circuits. **a**, Raster plot example of Neuropixels recordings for one experimental session (session ID 715093703). **b**, Sequence of states inferred by the HMM. **c**, Animal speed. **d**, Neural states across the entire recording session. Each row represents 1 min of activity from top to bottom, in parallel with the recording. Color represents the HMM inferred state at each given time. **e**, Same as **d** where color represents the animal speed. **f**, Neural activity of an example session for each neural state before (left) and after (right) performing LFA on the activity of each neural state. The three axes represent directions of the top three principal components after performing principal components analysis. The color code is the same as in **b** and **d**. **g**, Normalized covariances $m=\frac{\bar{c}}{\bar{a}}$ (see explanation of equation (1)) averaged across neurons and across the entire experimental session (orange) and averaged within each neural state (red). **h**, Same as **g** but for the normalized standard deviation of covariances $s=\frac{\delta c}{\bar{a}}$. **i**, Same as **g** for the relative ratio (*s* / (*s* + *m*)). **j**, Same as **g** for the dimensionality measured by the participation ratio (*D*_PR_), equation (1). The left scale (*y* axis) is extrapolated dimensionality to *N* → N^*^ = 10^4^ neurons, and the right scale (*y* axis) is percentage normalized to the number of neurons; dim. dimensionality. Boxes show median and interquartile range (IQR), and whiskers show 1.5× IQR. Overlaid points show individual observations.

To quantify dimensionality in vivo, we analyzed large-scale electrophysiology recordings from the mouse visual cortex, part of the Allen Brain Observatory^25,26^. In each experimental session, neural activity was recorded with six Neuropixels probes (from *N* = 125−520 neurons per session across the visual cortex, 319 ± 86 on average across 26 recorded sessions) while head-fixed mice freely behaved on a treadmill with no visual stimulus (gray mean luminance screen condition). We first observed that this activity appeared to follow a sequence of distinct states^38,39^, in which periods of roughly stationary neural activity were interrupted by abrupt changes (Fig. 1a,b and Supplementary Video 1). This suggests that dimensionality should be quantified within and across states, rather than as a single all-encompassing number.

**Extended Data Fig. 1:**
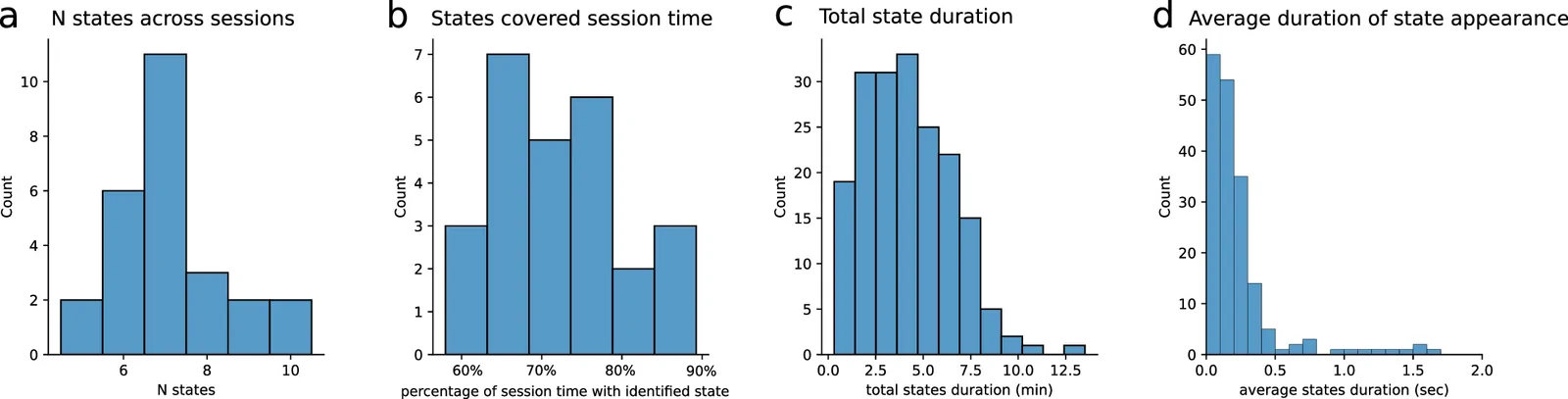
Hidden Markov Model (HMM) analysis of spontaneous activity in mouse visual cortex. This figure provides further details on the Hidden Markov Model (HMM) analysis used to infer various neural states from the spontaneous activity of the mouse visual cortex. This HMM analysis is the basis for the results presented in Fig. 1-Fig. 3 of the main text. The HMM algorithm extracts, in an unsupervised way, periods where the neural activity is well captured by the same latent state. It returns a parsing of 30 minutes of spontaneous periods of activity into multiple underlying states. In each state the hypothesis of the algorithm is that the average neural activity is steady. **a**) Histogram of number of detected states across all recording sessions. **b**) Histogram across all sessions of the fraction of session time (in percentage) covered by HMM states detected above the confidence threshold. **c**) Histogram of total durations, across all appearances of an individual state within a session, of states across all recording sessions. **d**) Histogram of average duration of individual occurrences of HMM states across all states and sessions.

We identified states using a hidden Markov model (HMM). Here, transitions among states are assumed to occur discretely via a Markov chain; within each state (colored intervals in Fig. 1b,d), neurons fire spikes as Poisson processes, with firing rates particular to that state. To restrict to clearly identified states, we retained only time bins for which the likelihood of being in the identified state (HMM’s posterior probability) was at least 80% (time bins where no state satisfied this criterion were left unassigned). In each session, the number of states was set via an unsupervised cross-validation procedure (7.4 ± 1.7 across 26 sessions, which ranged from 5 to 11 patterns; see Methods and Extended Data Fig. 1). Neural states appeared separated in the space of population responses (Fig. 1f) and were influenced by animal behavior (for example, running; Fig. 1c).

**Extended Data Fig. 2:**
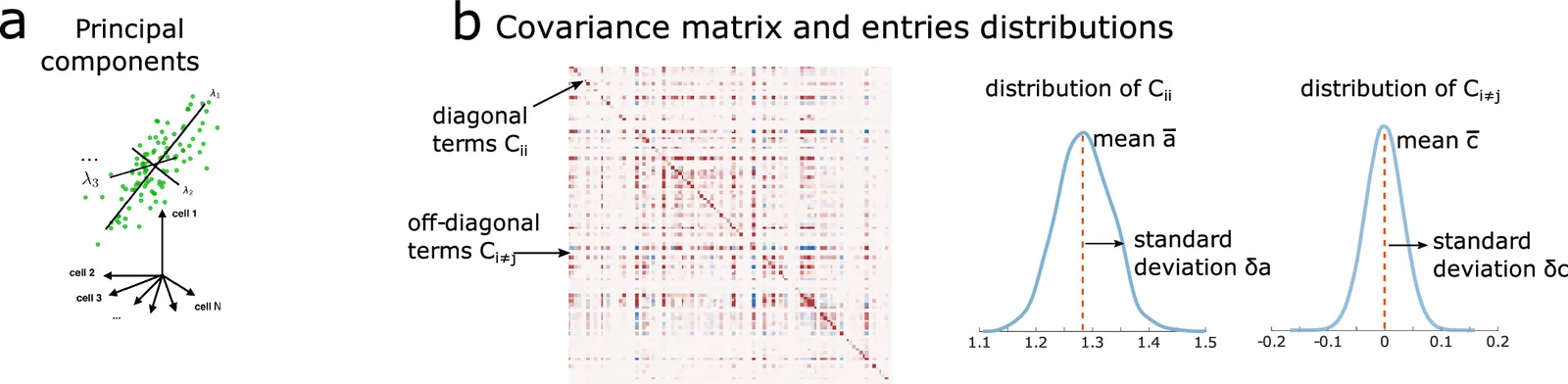
Statistics of covariances and dimensionality estimation. This figure introduces the concept of dimensionality of neural activity and its relationship to the statistics (means and standard deviations) of activity covariances. **a**) Example of data distribution where each green dot corresponds to a single vector of activity. In our experimental analyses, such a vector contains the spike counts of all neurons in a window of 100ms duration. The geometrical interpretation for such a point cloud is that the axes of major variability are determined by the eigenvectors of the covariance matrix. Eigenvalues, when normalized to their sum, determine the amount of variance of the data in the direction identified by the eigenvector. **b**) Example of spike-count covariance matrix and statistics. The two distributions on the right capture respectively the statistics of diagonal (variances) and off-diagonal (covariances) entries of the covariance matrix. Of specific importance for our study are the standard deviation of covariances *δc* and the mean of variances $\bar{a}$ as their ratio, here termed *s*, is in one-to-one correspondence with the recurrency (spectral radius) *R*.

We computed the widely used participation ratio *D*_PR_^3,4,11,13,40^ (see Methods and Extended Data Fig. 2) to measure dimensionality. *D*_PR_ is defined via the percentages of explained variance of activity along the principal components, given by the eigenvalues *λ*_*i*_ of the covariance matrix of neural activity. Importantly, *D*_PR_ can also be expressed exactly in terms of the statistics of entries in this covariance matrix *C*, which can be tractably measured and modeled (Extended Data Fig. 2b). Specifically, with *N* the number of neurons analyzed, the participation ratio is (see Methods and refs. ^4,13^)

1 $${D}_{{\rm{PR}}}(C)=\frac{{\left({\sum }_{i}{\lambda }_{i}\right)}^{2}}{{\sum }_{i}{\lambda }_{i}^{2}}=\frac{N}{1+{v}^{2}+(N-1)({m}^{2}+{s}^{2})}\,.$$

Here, *v*, *m* and *s* are, respectively, the ratios between the standard deviation (*δa*) of variances (diagonal elements of *C*), the average ($\bar{c}$) of covariances (off-diagonal elements of *C*) and the standard deviation (*δc*) of covariances to the average ($\bar{a}$) of variances: $v=\frac{\delta a}{\bar{a}}$, $m=\frac{\bar{c}}{\bar{a}}$ and $s=\frac{\delta c}{\bar{a}}$. Here, $\bar{a}$ may be thought of as a firing rate normalization. Equation (1) shows that, for large populations *N*, the factors *m* and *s* are the key factors modulating dimensionality. Although each appears equally in the formula, the two terms may have largely different origins. For example, a high *m* can be driven by a relatively small number of ‘low-rank’ behavioral components^10,41^, while a high *s*, as we will show, may result from strong recurrent connections among neurons.

Thus, we computed *m* and *s* in the neural recordings and asked whether either dominates. We first computed values across the entire period of activity (that is, across states) and separately within each neural state (Fig. 1g,h). As expected due to state transitions, *m* was substantially greater across states than within them. The same was true for *s*, although to a much lesser extent. Overall, *s* dominated *m* (Fig. 1i), with the ratio *s* / (*s* + *m*) exceeding 90% within states. This is consistent with previous findings for cortical activity data^23,24^ and models of cortical networks^18,22,24,42,43^. We verified that this trend becomes even stronger when we remove remaining low-rank components of the covariance via a cross-validated latent factor analysis (LFA) for each neural state (Supplementary Information and Supplementary Figs. 1–3).

**Fig. 2:**
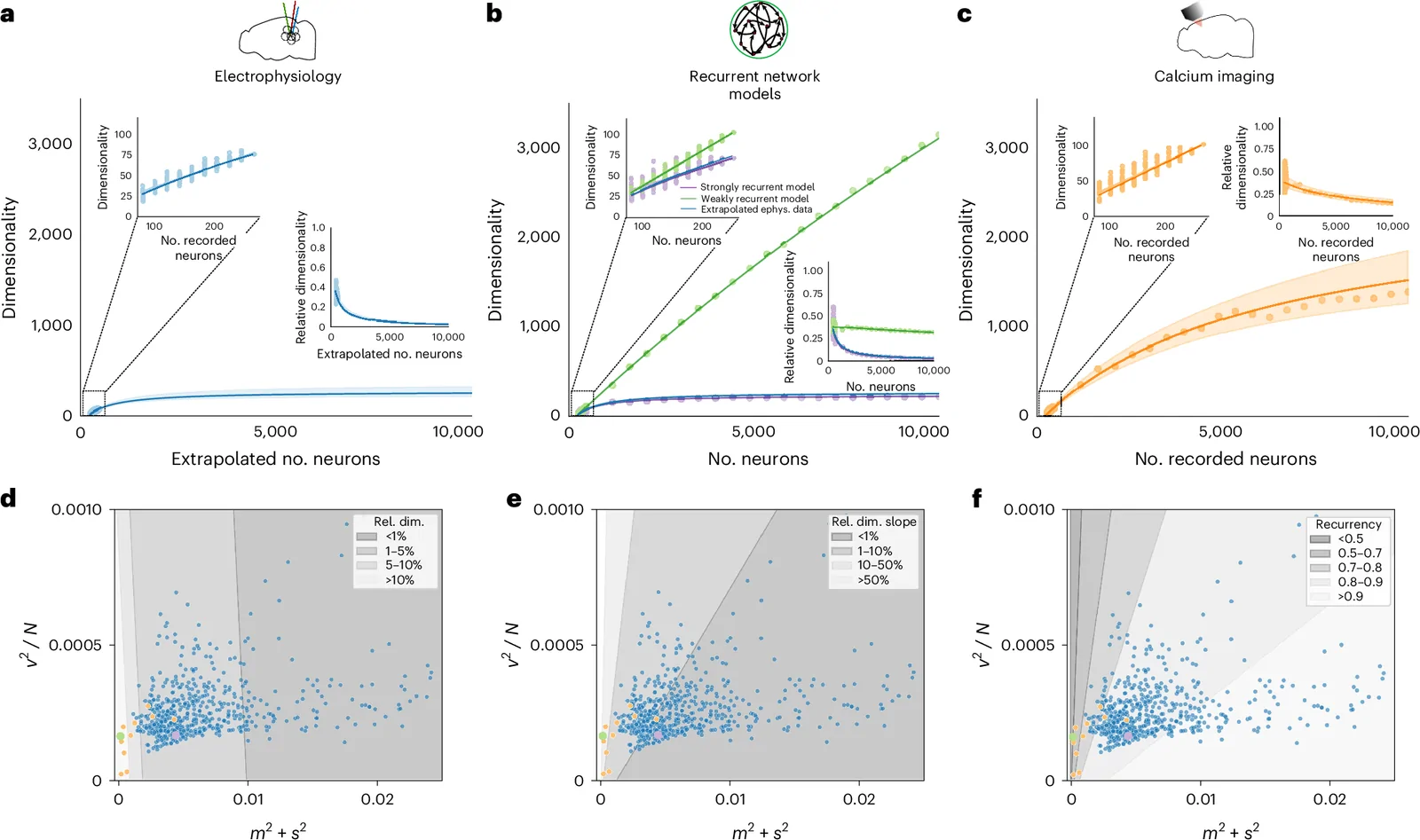
Dimensionality estimates based on subsampling and extrapolation. **a**, Example state from one session of visual cortex data. The left inset shows dimensionality (participation ratio) based on 20 different random subsamples (markers) of recorded neurons, as a function of subsample size. The light blue line indicates linear fit, and the dark blue curve indicates dimensionality extrapolation with equation (1) based on the largest possible sample (*N* = 275). The main graph shows dimensionality extrapolation (participation ratio) with equation (1) toward larger numbers of neurons based on the largest possible sample only (dark blue curve) and based on all subsamples (light blue error band, showing mean ± 1 s.d.). The right inset shows the same as the main graph but for dimensionality relative to the number of neurons. **b**, Network models with strong (purple) or weak (green) recurrent connectivity. The same dimensionality analysis was performed as for the experimental data in **a**. Here, dimensionality extrapolation of the participation ratio *D*_PR_ is based on *N* = 275 (dark green and dark purple curves; the dark purple curve lies below the dark blue curve from experimental data). It can be directly compared to and agrees well with actual dimensionality measurements (markers) from larger subsamples of model activity. The dependence of the participation ratio on the number of neurons, including the different behavior for strongly and weakly recurrent networks, is very similar for metrics of dimensionality based on variance explained (Extended Data Fig. 4); ephys., electrophysiology. **c**, Same analysis as in **a** but for an example session of two-photon calcium imaging data in the mouse frontal cortex. The extrapolation and insets are based on a random subsample of *N* = 275 neurons to mimic the scenario in **a**. As in the model data in **b**, dimensionality extrapolation based on *N* = 275 can be directly compared to and agrees well with actual dimensionality measurements (markers) from larger subsamples (see Extended Data Fig. 5 for statistical quantification across sessions and states). The yellow error band indicates extrapolation mean ± 1 s.d. across all subsamples of *N* ≤ 275 as in **a**. **d**, Extrapolated relative dimensionality at N^*^ = 10^4^. **e**, Dimensionality slope at N^*^ = 10^4^ relative to the slope at *N* = 275; Rel. dim., relative dimensionality. **f**, Inferred recurrency, as a function of covariance statistics *m*^2^ + *s*^2^ and *v*^2^ for all states in the visual cortex (blue markers, electrophysiology), the network models with strong (purple) and weak (green) recurrent connectivity and for running and rest states of two-photon calcium imaging data in the frontal cortex (yellow).

**Extended Data Fig. 3:**
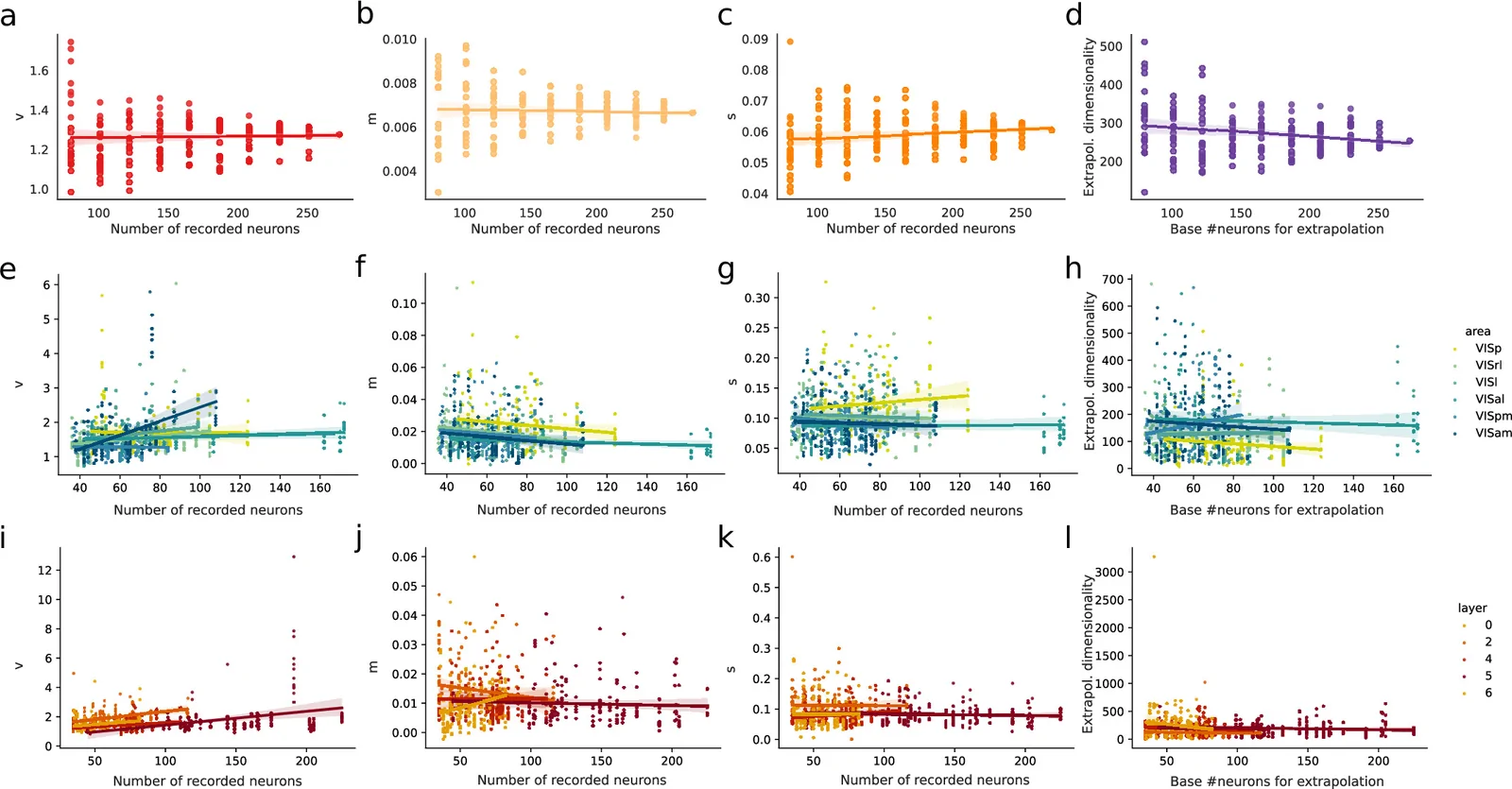
Dependence of covariance statistics and dimensionality on number of recorded neurons. This figure shows that the statistics of variances *v* and covariances *m* and *s* as well as the extrapolated dimensionality (participation ratio) do not strongly depend on the number of recorded neurons. In contrast to non-extrapolated dimensionality measures that show a strong almost linear growth with number of recorded neurons in the experimentally accessible regime (see Fig. 2 and Extended Data Fig. 9), the extrapolated participation ratio is not biased by the subsampling of visual cortical neurons and therefore a more appropriate method for comparing distinct sessions, regions, and layers, as shown in Fig. 1, Fig. 2 and Fig. 3 of the main text. **a**-**d**) Example state of one recording session in visual cortex. **a**) Variance of variances *v* (markers) calculated from 20 different random neuron subsamples per subsample size including linear fit (straight line). **b**) Mean covariance *m* (markers) calculated from 20 different random neuron subsamples per subsample size including linear fit (straight line). **c**) Variance of covariances *s* (markers) calculated from 20 different random neuron subsamples per subsample size including linear fit (straight line). **d**) Extrapolated dimensionality at 10^4^ neurons as calculated from 20 different random neuron subsamples per subsample size including linear fit (straight line). **e**-**h**) Same as panels a-d but for all HMM states, resolved across cortical areas. **i**-**l**) Same as panels a-d but for all HMM states, resolved across cortical layers.

Finally, we combined the contributions of the variance *v* and covariance *m* and *s*, together with the number of neurons analyzed *N*, to compute the dimensionality *D*_PR_(*C*) (equation (1)). For the given number *N* of recorded neurons in each session, this yields a value for the dimensionality of the recorded data. According to equation (1), we expect that a different, smaller dimensionality would have been found if one recorded fewer neurons (but with these same variance and covariance statistics). This was indeed the case. We took random subsamples of the recorded neurons and calculated the covariance statistics *v*, *m* and *s* based on these and then used formula equation (1) to compute the corresponding dimensionality, shown for one example state in Fig. 2a (left inset). Although the variance and covariance statistics did not show any trend with the number of recorded neurons (Extended Data Fig. 3), the dimensionality shrank when taking into account fewer neurons (Fig. 2a), as predicted by the explicit dependence on *N* of equation (1).

**Extended Data Fig. 4:**
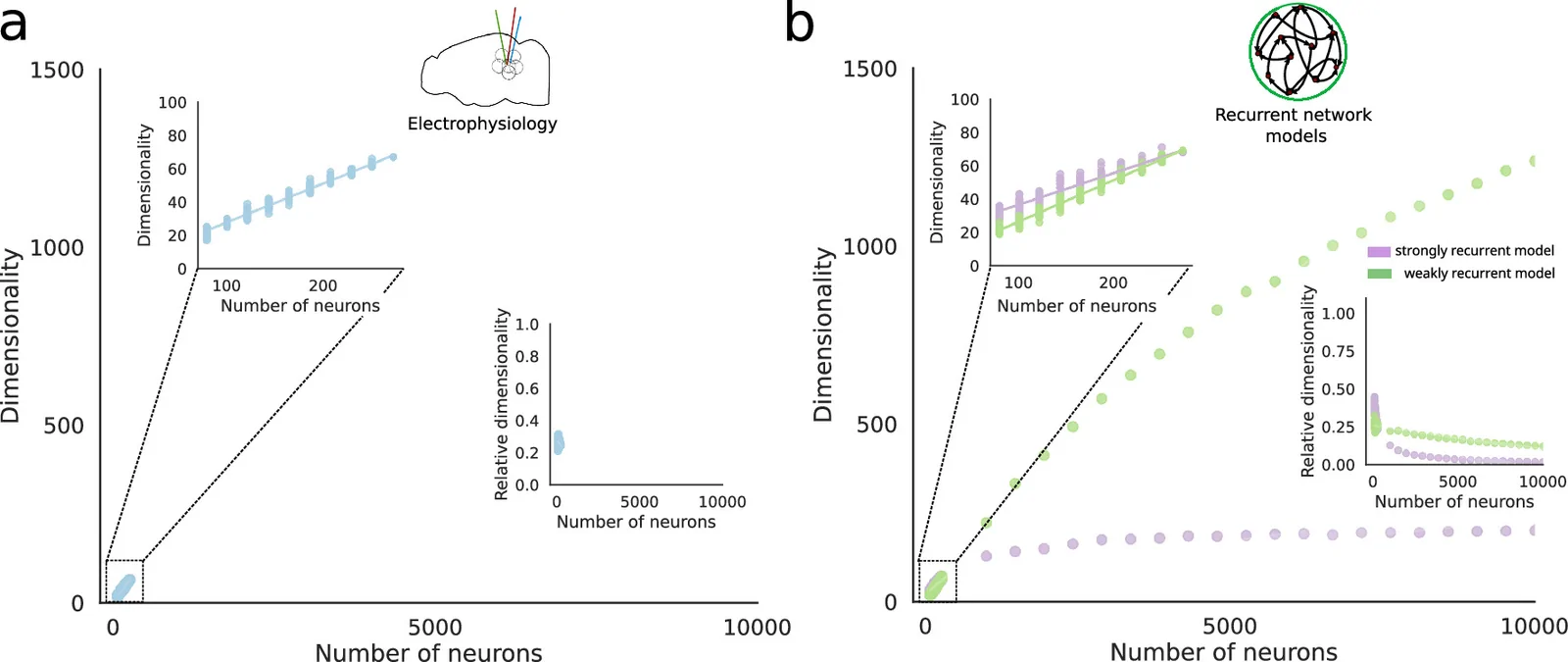
Dependence of dimensionality in electrophysiological data (a, blue) and in network models (b) with strong (purple) and weak (green) recurrency on the number of neurons taken into account for analysis. This figure is analogous to main text Fig. 2a and Fig. 2b, but dimensionality is measured as the number of components required to explain 70% variance rather than the participation ratio. The figure shows that results are qualitatively the same as for the participation ratio. In particular, weakly and strongly recurrent networks behave very similarly for the numbers of neurons experimentally accessible here. For larger numbers of neurons, the weakly recurrent network continues to show a strong growth in dimensionality, while the dimensionality of the strongly recurrent network shows a saturation. For large numbers of neurons, the relative dimensionality of the weakly recurrent network stays high, while the relative dimensionality of the strongly recurrent network shrinks to very small values. Left insets: Dimensionality based on 20 different random subsamples (markers) as a function of subsample size for experimentally accessible sample sizes. Colored straight lines are linear fits. Main panel: Dimensionality as a function of subsample size for the range up to N^*^ = 10^4^. This analysis is only feasible in the models. No extrapolation from subsamples is available. Right inset: Same as main panel, but for dimensionality relative to the number of neurons.

Similarly, increasing *N* beyond the number of recorded neurons yields estimates of the dimensionality in the local circuit from which the multiple Neuropixels probes simultaneously sample. The *N* dependence of equation (1) allows us to achieve this (Fig. 2a and Methods). This extrapolation toward larger numbers of neurons similarly assumes that the measured covariance statistics remain constant and representative for a local network of larger size than the specific number of neurons recorded in a given session. Figure 2a shows this extrapolation and demonstrates an interesting effect: as the number of neurons *N* increases, the dimensionality grows more slowly than *N*, so that the relative dimensionality (*D*_PR_(*C*) / *N*) shrinks. Mathematically, this is due to the presence of *N* in the denominator of equation (1); intuitively, it is because the effects of a given level of covariance among neurons in a population of a given size places greater constraints on the population’s activity patterns for larger population sizes, as the increasing number of positive and negative correlations among many pairs of neurons compound in their effect. We note that this phenomenon also holds for other popular dimensionality measures, such as those based on the explained variance (Extended Data Fig. 4), but may not necessarily hold for every measure of dimensionality^44^.

Figure 2a shows that *D*_PR_(*C*) saturates by a few thousand neurons, which is a low number compared to the number of neurons in the visual cortex areas from which the up to six Neuropixels probes simultaneously record. As a conservative choice, we take N^*^ = 10^4^ when analyzing data and modeling networks below (Methods), achieving in turn a conservative upper bound for the relative dimensionality. Before proceeding, we conduct two checks to verify our methods for extrapolating *D*_PR_(*C*) to N^*^ = 10^4^. As a first check, we use this N^*^ = 10^4^ value in a network model (Methods) to confirm that the dimensionality of a network of this size can be correctly extrapolated from subsamples of a similar size as in the recordings (Fig. 2b, purple). As a second check, we directly verified our method for extrapolating dimensionality using a distinct, complementary experimental dataset. To obtain sample sizes up to and beyond N^*^ = 10^4^ from a local area, we made recordings with two-photon calcium imaging.

**Extended Data Fig. 5:**
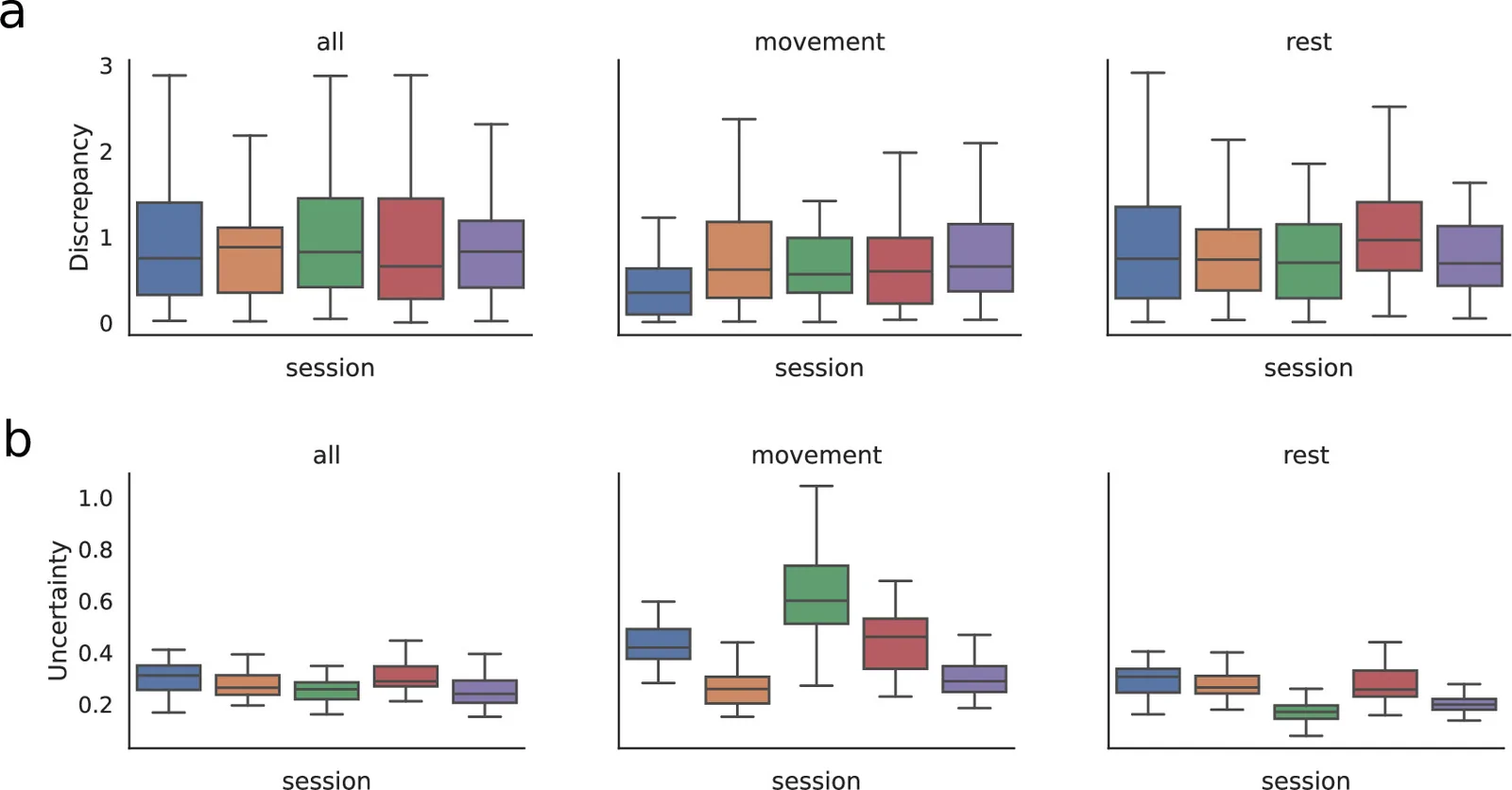
Validation of dimensionality extrapolation in two-photon calcium imaging data. Statistics are obtained from *n* = 50 different random neuron subsamples *i* of size 275 for each recording session. Boxes show median and IQR, whiskers show 1.5 × IQR. **a**) Statistics of $$| {D}_{\mathrm{PR},i}^{\mathrm{extrapol}}-{D}_{\mathrm{PR}}^{\mathrm{true}}| /{\mathrm{std}}_{j}$$$$({D}_{\mathrm{PR},i,j}^{\mathrm{extrapol}})$$, that are the absolute discrepancies between the extrapolated participation ratio $${D}_{{\rm{PR}},i}^{{\rm{extrapol}}}$$ (cf. solid yellow line in Fig. 2c) and the true participation ratio $${D}_{{\rm{PR}}}^{{\rm{true}}}$$ of all recorded neurons (cf. rightmost yellow marker in Fig. 2c), measured relative to the standard deviation $${{\rm{std}}}_{j}({D}_{{\rm{PR}},i,j}^{{\rm{extrapol}}})$$ of the extrapolated dimensionalities (uncertainty, yellow band in Fig. 2c) obtained from further random subsamples *j* of each 275-neuron sample *i*. The difference between the true dimensionality at large recording sizes and the extrapolated value is on average within the uncertainty estimated from further subsampling, that is the true dimensionality lies on average within the yellow uncertainty band around the extrapolated value in Fig. 2c. b) Statistics of $${{\rm{std}}}_{j}({D}_{{\rm{PR}},i,j}^{{\rm{extrapol}}})/{D}_{{\rm{PR}}}^{{\rm{true}}}$$, that are the uncertainties in extrapolated dimensionalities *D*_PR,*i*,*j*_ relative to the size of the true dimensionality $${D}_{{\rm{PR}}}^{{\rm{true}}}$$. The median uncertainty (standard deviation) is below 30% of the true dimensionality value for most sessions and conditions. The yellow uncertainty band in Fig. 2c is therefore typically much smaller than the true dimensionality value. Low vs high relative dimensionalities at large recording sizes can be thus reliably be distinguished with the dimensionality extrapolation procedure. Columns refer to data not segmented according to movement and rest states (left), movement states only (center), and rest states only (right).

The recordings were performed in the mouse frontal cortex during spontaneous activity (Methods). To mimic the analyses of our electrophysiological recordings, we considered a random subsample of imaged neurons of the same size (*N* = 275) as used in Fig. 2a and performed the same analysis. The important difference is that the dimensionality extrapolation curve, which, as above, is based on the subsample of *N* = 275 neurons, can be directly compared to real measurements of dimensionality of larger sample sizes up to N^*^ = 10^4^ (compare markers with the solid line in Fig. 2c). We find good agreement of the extrapolated and true dimensionalities. We further statistically quantified this agreement by repeating the procedure in Fig. 2c for 50 different random subsamples of size *N* = 275 for each recording session (Extended Data Fig. 5a). The true dimensionality value was found to be on average within 1 s.d. from the extrapolated value. This standard deviation, obtained for each individual subsample by further subsampling (compare the yellow area in Fig. 2c and left inset) thus serves as a measure of uncertainty of the extrapolation. The size of this standard deviation was furthermore consistently much smaller than the true dimensionality value (Fig. 2c and Extended Data Fig. 5b; median 28% across sessions and states). Note that the precise values of extrapolated dimensionality in Fig. 2c are not meant to be directly compared to those in Fig. 2a, as they arise from a different recording modality and brain area. Rather, Fig. 2c verifies that the true dimensionality regime measured directly at these larger local network sizes can be faithfully predicted from smaller sample sizes with our dimensionality extrapolation procedure.

**Fig. 3:**
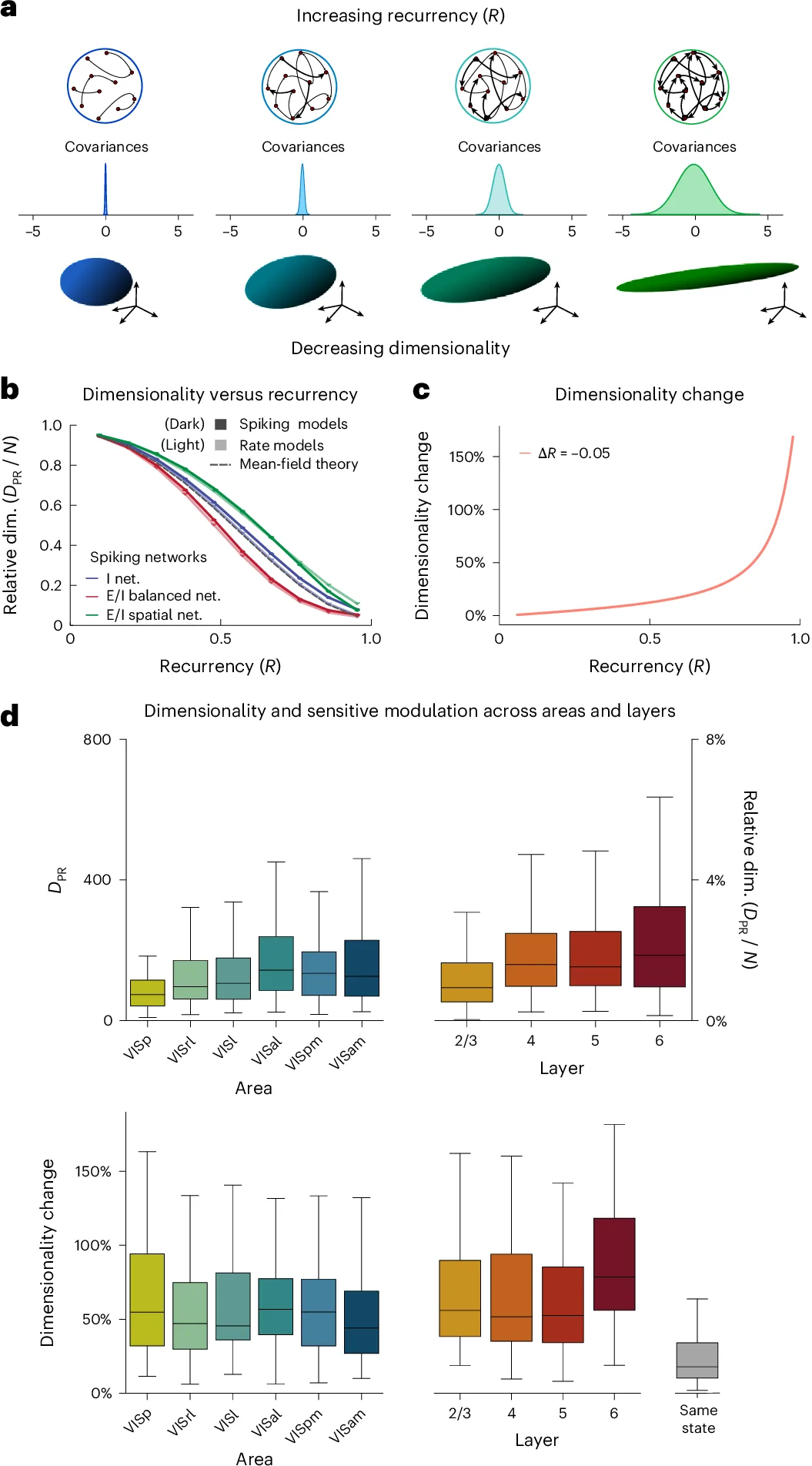
Dimensionality and recurrency *R* across the visual cortical circuit. **a**, Three-way connection between recurrency, width of covariance distribution and dimensionality of neural activity. **b**, Normalized dimensionality *D*_PR_ / *N* as a function of the recurrency *R*. Theoretical predictions for the dimensionality in homogeneous inhibitory networks (gray) are accurate for simulations of rate models (light colors) and spiking models (dark colors) across various network topologies (blue, homogeneous single-population inhibitory networks; red, homogeneous two-population excitatory–inhibitory networks; green, spatially organized single-population inhibitory networks); E, excitatory; I, inhibitory; net. network. **c**, Dimensionality change (relative modulation of dimensionality) as a function of recurrency (Δ*D*_PR_). **d**, Dimensionality extrapolated to a network size of 10^4^ neurons (top) and dimensionality change (relative modulation of dimensionality) between neural states (bottom) across visual cortical areas and cortical layers; control (gray bar, right-most plot), dimensionality change computed between multiple instances of the same neural state. Boxes show median and IQR, and whiskers show 1.5× IQR. Statistics (top): primary visual cortex (VISp) *n* = 145; rostrolateral visual cortex (VISrl) *n* = 109, lateral visual cortex (VISl) *n* = 110, anterolateral visual cortex (VISal) *n* = 112, posteromedial visual cortex (VISpm) *n* = 98, anteromedial visual cortex (VISam) *n* = 152, layer 2/3 *n*  = 143, layer 4 *n* = 157, layer 5 *n* = 170 and layer 6 *n* = 136. Statistics (bottom): VISp *n* = 588, VISrl *n* = 382, VISl *n* = 396, VISal *n* = 488, VISpm *n* = 410, VISam *n* = 610, layer 2/3 *n* = 544, layer 4 *n* = 624, layer 5 *n* = 662, layer 6 *n* = 530, same-state control *n* = 240.

**Extended Data Fig. 6:**
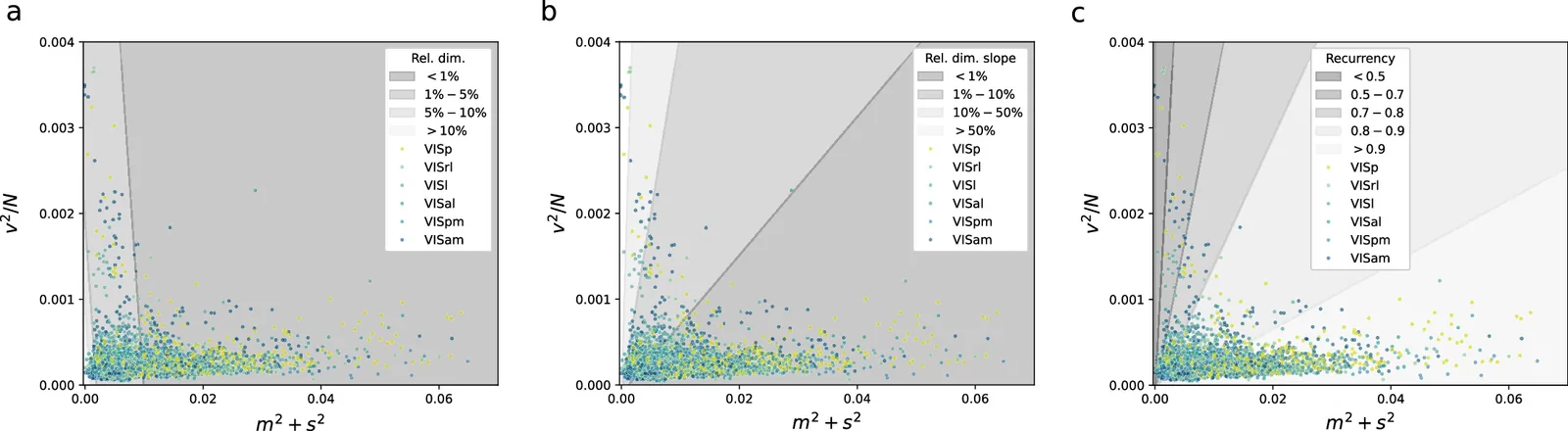
Area-resolved analysis of relative dimensionality, dimensionality slope and inferred recurrency. This figure shows an analogous analysis as in Fig. 2d–Fig. 2f, with the difference that data is subdivided into individual visual cortical areas. **a**) Extrapolated relative dimensionality at N^*^ = 10^4^, **b**) dimensionality slope at N^*^ = 10^4^ relative to the slope at *N* = 275, and **c**) inferred recurrency, as a function of covariance statistics *m*^2^ + *s*^2^ and *v*^2^ for all states across visual cortical areas.

**Extended Data Fig. 7:**
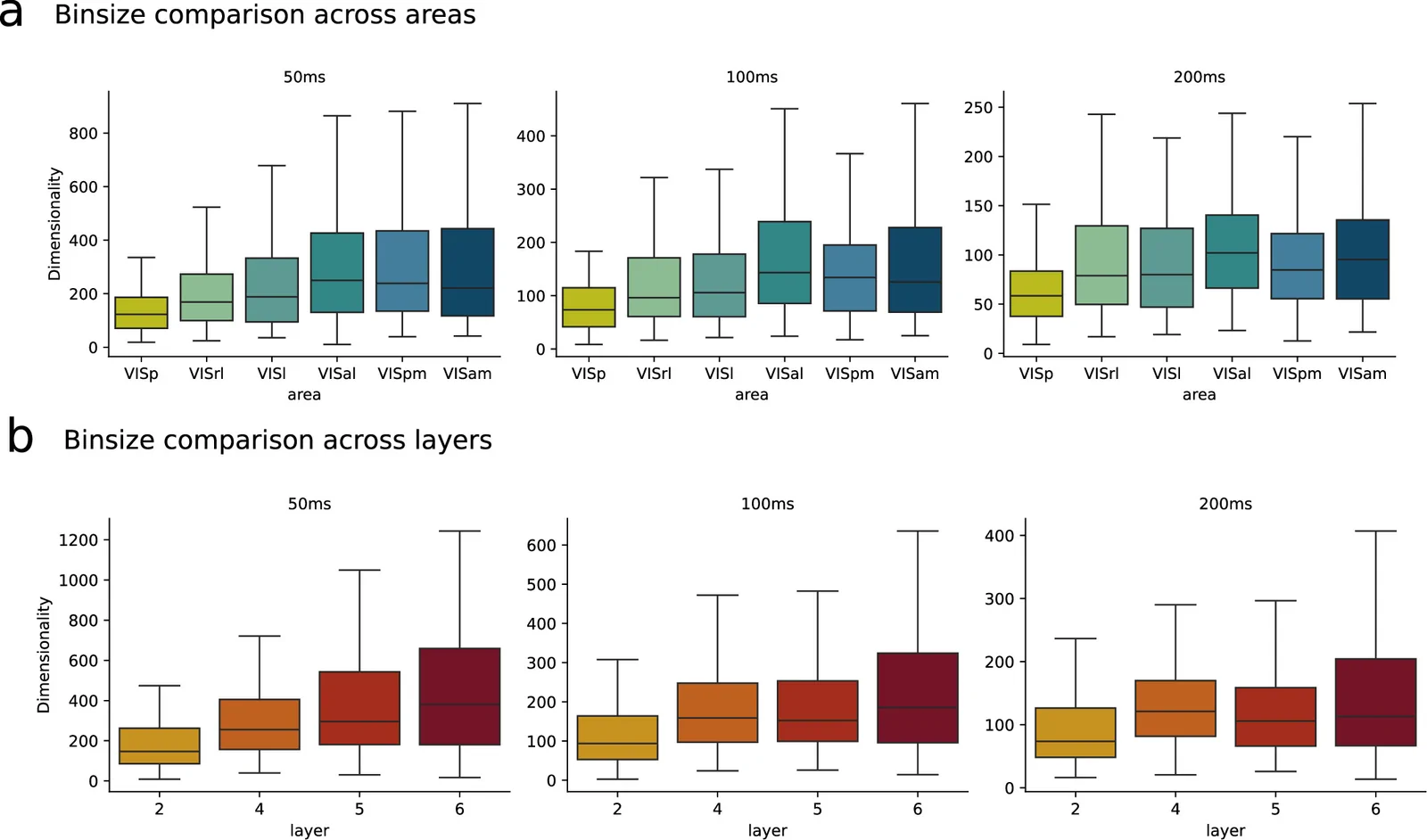
Dependence of dimensionality on bin size. **a**) Dimensionality of activity in different visual areas for bin sizes of 50ms (left), 100ms (middle) and 200ms (right). **b**) Dimensionality of activity in different cortical layers for bin sizes of 50ms (left), 100ms (middle) and 200ms (right). Boxes show median and IQR, whiskers show 1.5 × IQR. Same n-values as in Fig. 3d top.

Given this verification of our dimensionality estimation procedure from both the model and in distinct calcium imaging data, we proceeded in using N^*^ = 10^4^ neurons to compute the dimensionality *D*_PR_(*C*), the relative dimensionality *D*_PR_(*C*) / *N* and the saturation behavior of dimensionality curves for all sessions and states in the electrophysiological data from the visual cortex (Figs. 1j and 2d,e). The small relative dimensionalities found at realistic network sizes were robustly observed across all HMM states and all recording sessions in the visual cortex and were consistent when computed across both cortical layers and areas (Fig. 3d and Extended Data Figs. 3 and 6) and different bin sizes (Extended Data Fig. 7).

The predicted saturation of dimensionality and small relative dimensionalities are striking phenomena, and we next argue that they are not generic consequences of extrapolating the dimensionality of networked systems but rather occur for networks constrained by variance and covariance statistics in the regime that we measured experimentally.

Specifically, we next fit a modified network model with weaker recurrent connectivity (Fig. 2b, green), designed to preserve the statistics of variance *v* and mean covariance *m* but to have a different, smaller value of *s*. The initial growth of dimensionality with *N* appears qualitatively similar for the two network models (Fig. 2b, left inset). This makes sense, as equation (1) shows that, for small *N*, this value is largely set by the heterogeneity in firing rates reflected in *v* (ref. ^45^), which is the same in both models and much larger than *m* and *s* (Extended Data Fig. 3). However, the trends in dimensionality across larger numbers of neurons are strikingly different (Fig. 2b, middle), with only the original network model with all three covariance statistics as measured in the data showing saturating values of dimensionality and low values of relative dimensionality across the shown range of *N*. This is because, for larger *N*, the covariance statistics *m* and *s* dictate the dimensionality. Extended Data Fig. 4 shows similar results for an alternative measure of dimensionality.

In sum, we have argued thus far that the covariance statistics recorded from cortical data predict low relative dimensionality across populations at the scale of thousands of neurons and that these phenomena are not generic in network models but can emerge systematically.

### Strong recurrence as a mechanism for low relative dimensionality

What is the mechanistic origin of the strongly constrained dimensionality of neural activity that we predict? Our analyses above suggest that it lies in the underlying recurrent network connectivity (Fig. 3a) and how this sets the main quantity *s* determining dimensionality. Previous studies^18,43^ showed that *s* is determined by the level of network recurrency for a given neural state: as network recurrency becomes stronger, longer and longer synaptic paths impact the covariation in activity between each pair of neurons. These longer paths are highly variable from one neuron pair to the next, and this variability drives a wide range in the covariances across neural pairs, precisely the quantity that *s* measures (Fig. 3a).

**Extended Data Fig. 8:**
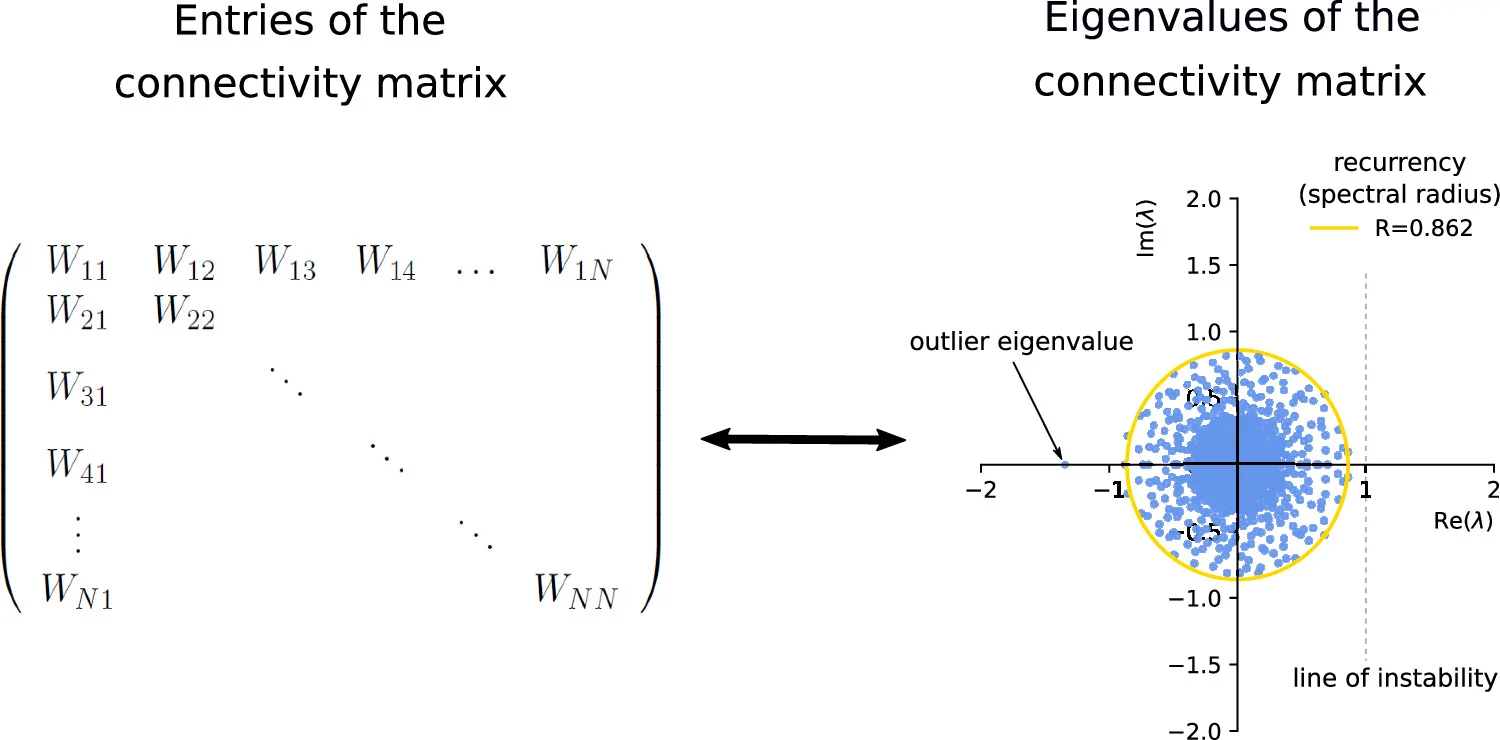
Illustration of recurrency (spectral radius). Network connectivity can be equivalently characterized by the entries *W*_*ij*_ of the connectivity matrix (left) or the eigenvalues *λ*_*i*_ of the connectivity matrix (right). The figure shows an example eigenvalue spectrum, that is the distribution of eigenvalues in the complex plane, for a random sparse excitatory-inhibitory network without motifs. Here, heterogeneity of connectivity induced by sparseness creates the bulk of eigenvalues, a disc (yellow circle) whose radius is called the spectral radius or recurrency. Additional network structure, such as the ratio between excitation and inhibition, can generate outlier eigenvalues outside the bulk. In networks with excess inhibition, these outlier eigenvalues are negative, such that the extent of the bulk, that is the recurrency, and its distance to the line of instability at ${\rm{Re}}(\lambda )=1$ determine the stability of the circuit. In this study we show how, in addition to sparseness, other connectivity statistics, such as motifs, influence the recurrency of neural circuits.

This intuition, linking covariation in neural activity to underlying synaptic paths, can be formalized by means of quantifying recurrency through a single number *R*, the spectral radius of the effective network connectivity. *R* characterizes the distribution of eigenvalues of the effective connectivity matrix, which in turn is determined by the statistics of network connections and the overall strength of recurrent coupling (Extended Data Fig. 8 and Methods). Via a three-way link between low-dimensional neural activity (Fig. 3a, bottom), large variance of correlations (Fig. 3a, center) and strong recurrent connections (Fig. 3a, top), there is a direct relationship between dimensionality (extrapolated to or measured at the full network size *N*) and recurrency *R*:

2 $${D}_{{\rm{PR}}}/N={(1-{R}^{2})}^{2}.$$

See the Supplementary Information for a formal derivation based on ref. ^18^ and see ref. ^21^ for an alternative derivation. This relationship is robust, as shown by our validation in nonlinear spiking networks, and holds for networks with a wide range of topologies (Fig. 3b).

In turn, the relationship between dimensionality and recurrency suggests that the low relative dimensionality of cortical activity (Figs. 1j and 2d) can be explained if visual cortical networks operate in a strongly recurrent regime^46^, where *R* is close to the critical value *R* = 1 where networks become linearly unstable. In fact, a quantitative inference of recurrency (Methods) yields a recurrency larger than 0.8 for 93% of all states in the visual cortex and *R* > 0.9 for 17% of the states (Fig. 2f).

Note that our results from the frontal cortex generally suggest a slightly larger dimensionality and smaller recurrency than in the visual cortex (Fig. 2a,c,d,f). Quantitative comparisons between the visual cortex and frontal cortex, however, have to be taken with caution here as the measurement modalities for the two regions differ. In particular, normalization of fluorescence signals could distort the results. Therefore, the calcium data should be solely regarded as a validation for the dimensionality extrapolation procedure here.

The conclusion of strong recurrency in the visual cortex is also robust in scenarios where the dimensionality is substantially modulated by large variability in firing rates, as reflected in a large *v* (Fig. 2f) and under assumptions about inputs to networks (Supplementary Figs. 3 and 5). Specifically, we fit two types of network models to visual cortex data (Supplementary Information). The first type assumes that all activity is generated intrinsically, whereas the second considers low-rank input components shared across many neurons. Both models can quantitatively reproduce the experimentally observed covariance statistics and dimensionality (Supplementary Fig. 4). Even when all low-rank activity components are attributed to inputs, we still find that strong recurrency is required to produce the activity statistics, in particular the high value of *s*, observed in neural recordings (Supplementary Fig. 4).

### The sensitive regime for modulation of dimensionality

In the same strongly coupled regime, the relative change in dimensionality with respect to a change Δ*R* in recurrency *R* is highest. Figure 3c illustrates this via the quantity $\Delta {D}_{{\rm{PR}}}(R,\Delta R)=\frac{2| {D}_{{\rm{PR}}}(R)-{D}_{{\rm{PR}}}(R+\Delta R)| }{{D}_{{\rm{PR}}}(R)+{D}_{{\rm{PR}}}(R+\Delta R)}$. For low and medium values of recurrency, a small change Δ*R* does not yield a strong relative change Δ*D*_PR_ in dimensionality. However, when *R* increases to larger values, neural networks acquire a sensitive control of dimensionality: the capacity to sharply regulate dimensionality via underlying effective connectivity features that impact the recurrency. As above, we verified that this strong sensitivity remains in the presence of low-rank network inputs consistent with the neural recordings (Supplementary Fig. 5b).

But how can the recurrency of a network rapidly change? One natural possibility arises from the nonlinear input–output behavior of neurons^47^. A change in the activity level of a neuron induces a change in its gain and thus the effective strength of recurrent coupling (and, in turn, the quantity *R*)^43^. Our finding that the cortex operates in a strongly recurrent regime where dimensionality is under sensitive control therefore suggests that different cortical states, defined by different firing rate patterns across neurons, should produce notably different dimensionalities. To test this, we measured the dimensionality change between any two states *A* and *B* in the same session, $\Delta {D}_{{\rm{PR}}}=\frac{2| {D}_{{\rm{PR}}}(A)-{D}_{{\rm{PR}}}(B)| }{{D}_{{\rm{PR}}}(A)+{D}_{{\rm{PR}}}(B)}$. Values close to or above 50% for Δ*D*_PR_, as found in our analysis (Fig. 3d), imply that dimensionality changes across the two states are substantial, on average roughly half or more as large as the dimensionalities themselves.

Importantly, the states we extract from neural activity via the HMM are solely defined by neuronal firing rate patterns and not by the covariances among neurons. An overall change in firing rate does not directly influence our dimensionality measure, due to the firing rate normalization of *m* and *s*. Still, firing rates impact covariances and dimensionality indirectly by setting neuronal gains, which in turn affect the recurrency *R*. Our analyses show that such gain modulation is a particularly effective mechanism for recurrency and dimensionality control of neural circuits when they operate in the strongly recurrent regime.

### Local tuning of the global recurrency *R*

**Fig. 4:**
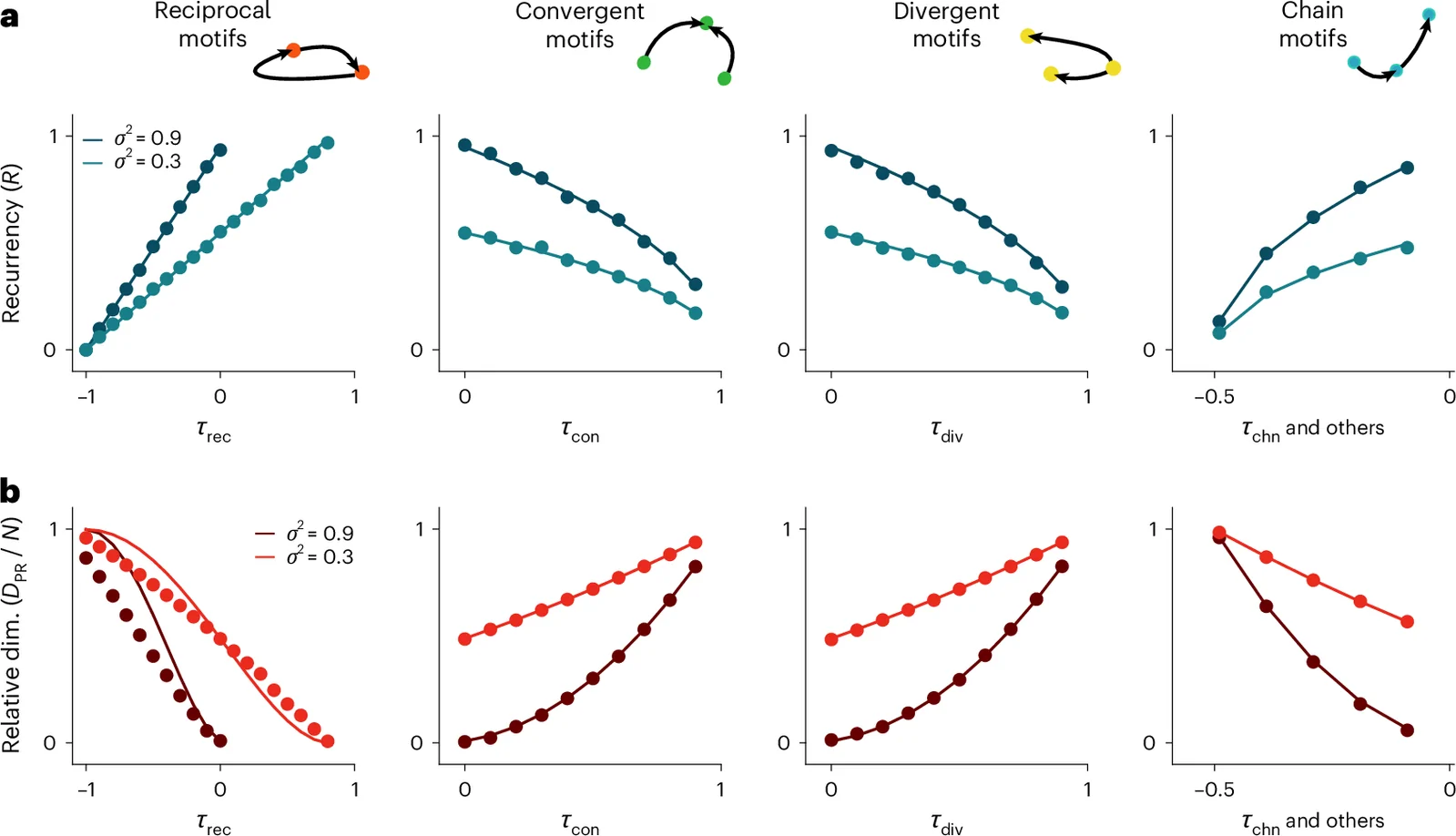
Theory for recurrency and dimensionality in networks with second-order motifs. **a**, Theoretical dependence of recurrency on abundances of reciprocal, convergent, divergent and chain motifs. **b**, Theoretical dependence of dimensionality on motif abundances. Solid lines indicate theory, and markers indicate simulations. The abundance of chain motifs *τ*_chn_ is constrained by theoretical limits, as reflected in the range of values of *τ*_chn_ (Supplementary Information).

We next asked which structural connectivity features of neural networks can regulate their overall recurrency *R* and hence their dimensionality (Fig. 4). Although many previous studies established how global features of recurrent connectivity affect *R* (refs. ^18,48,49^), here, we focus on the impact of local features of connectivity: connection probabilities and strengths and second-order connectivity motifs.

These motifs are statistics of the neural connectivity *W* that involve ‘pairs’ of connections (Methods) and together with overall connection probabilities form the fundamental local building blocks of networks. Second-order motifs appear in four types (Fig. 4a): reciprocal, divergent, convergent and chain motifs, together with the variance (strength) of neural connections already present in purely random models^48^. These motifs have been shown to play important roles determining neuron-to-neuron correlations and allied circuit dynamics^28–31,34,50–54^ and emerge from learning rules consistent with biological spike timing-dependent plasticity mechanisms^55,56^.

We developed a comprehensive theory that takes full account of all second-order motifs in networks of excitatory and inhibitory neurons, generalizing allied results developed via distinct theoretical tools ^21,53^. Our analysis yields a compact analytical quantity that shows how recurrency is modulated by local structure: *R* = *σ* ⋅ *R*_motifs_.

The first term, *σ*, stems from the strength and number of connections and has been studied before^57^ (Supplementary Information). The second,

3 $${R}_{{\rm{motifs}}}=\frac{1-{\tau }_{{\rm{div}}}-{\tau }_{{\rm{con}}}-2{\tau }_{{\rm{chn}}}+{\tau }_{{\rm{rec}}}}{\sqrt{1-{\tau }_{{\rm{div}}}-{\tau }_{{\rm{con}}}}}$$

compactly describes the influence of connectivity motifs in a form that is new here. In this equation ${\tau }_{{\rm{rec}}},\,{\tau }_{{\rm{chn}}},\,{\tau }_{{\rm{div}}},\,{\tau }_{{\rm{con}}}$ respectively capture the abundance of reciprocal, chain, divergent and convergent motifs (Methods and Supplementary Information). This formula describes how the recurrency *R* is affected by increasing or decreasing the prevalence of specific motifs (Fig. 4a) and thus links the modulation of covariances and the dimensionality of neural responses across the global network to the statistics of local circuit connectivity, as shown in Fig. 4b. Although equation (3) is exact for networks with homogeneous connection statistics, we show that it generalizes to models with distinct connection statistics for excitatory and multiple inhibitory neuron types. Here, *σ* and *τ* combine the corresponding statistics of the excitatory and inhibitory populations (Supplementary Fig. 6, Methods and Supplementary Information). This direct link between quantifiable, local connectivity statistics and the global network property *R* opens the door to novel functional analyses of very large-scale synaptic physiology datasets in both mice and humans, as we describe next.

### Local synaptic motifs modulate the recurrent coupling of cortical circuits in mice and humans

We analyzed newly released synaptic physiology datasets from both mouse and human cortex^37^ to assess the involvement of synaptic connectivity properties in modulating network recurrency. This dataset is based on simultaneous in vitro recordings of three- to eight-cell groups (Methods) and consists of 2,767 identified synapses from mouse primary visual cortex (of more than 32,000 potential connections that were tested) and 363 synapses from human cortex.

**Fig. 5:**
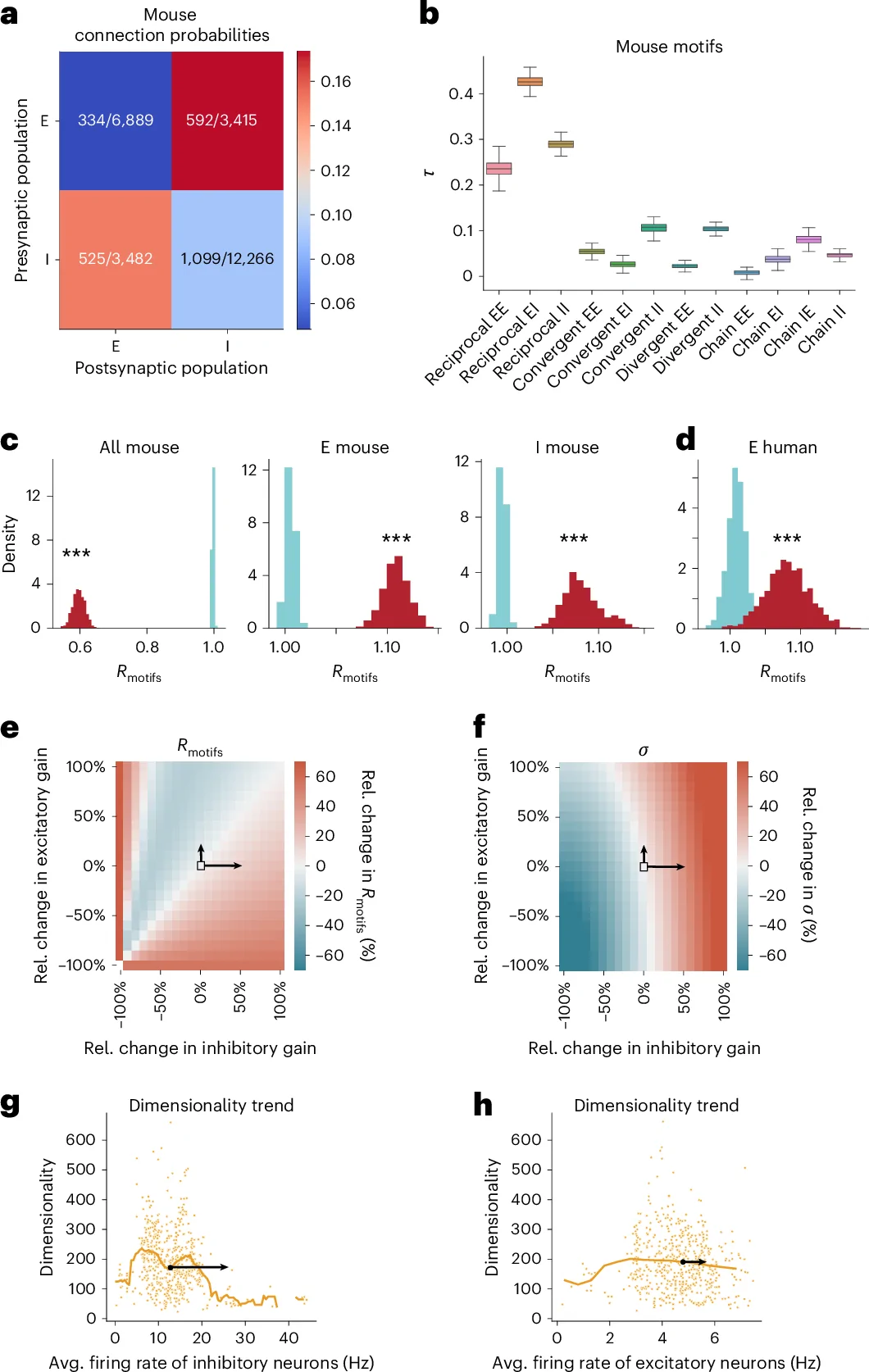
Motif analysis in synaptic physiology datasets. **a**, Statistics of mouse connectivity across populations (synapses identified/synapses probed). **b**, Population-resolved motif abundances in the mouse primary visual cortex. Two-synapse motifs are split by presynaptic excitatory/inhibitory class. Per-box *n* values are reported as probed motif opportunities/found motifs: reciprocal EE = 3,179/41, reciprocal EI = 3,123/263, reciprocal II = 5,576/158, convergent EE = 11,021/130, convergent EI = 9,663/100, convergent II = 17,622/315, divergent EE = 17,632/164, divergent II = 25,446/456, chain EE = 21,499/99, chain EI = 9,553/159 and chain II = 34,822/394. Boxes show median and IQR of 1,000 bootstraps based on random subsets of 80% of sessions, and whiskers show 1.5× IQR. **c**, Estimation of *R*_motifs_ from mouse data (red distribution; statistics across the same bootstraps as in **b**); blue distribution, estimation of *R*_motifs_ on 1,000 ensembles of randomly connected neurons (Methods). The data on the left show *R*_motifs_ computed using all synapses, both excitatory and inhibitory, in a balanced setting. The data in the middle and on the right consider the extreme scenarios, where only the excitatory or inhibitory population is active, corresponding to only excitatory or only inhibitory synapses contributing to *R*_motifs_ (E and I, respectively). Data were analyzed by two-sided *t*-test comparing bootstrap distributions; ***P < 0.001. **d**, Same as in **d** for excitatory motifs in the human dataset. **e**, Relative change in *R*_motifs_ when changing the gain of inhibitory or excitatory neurons. The cases (−100%, 0%) and (0%, −100%) correspond to the excitatory- and inhibitory-only cases in **c**. **f**, Relative change in *σ* when changing the gain of inhibitory or excitatory neurons. **g**, Dimensionality of visual cortex activity (extrapolated to *N* → N^*^ = 10^4^; compare Figs. 1 and 3) as a function of the average firing rate of putative inhibitory neurons in HMM states. Each marker corresponds to one individual state, the solid line is a moving average of the data in steps of 0.5 Hz with a window size of 3 Hz; Avg., average. **h**, Dimensionality of visual cortex activity (compare Figs. 1 and 3) as a function of the average firing rate of putative excitatory neurons in states defined via the HMM (Fig. 1).

From adjacency matrices inferred from groups of simultaneously patch-clamped cells, we obtained connection probabilities and motif coefficients *τ*. We distinguished between excitatory and inhibitory synapses and found that these have significantly different connectivity statistics (Fig. 5a,b). Although all types of motifs were overrepresented compared to the assumption of independence between synapses, we found that reciprocal connections are particularly abundant. Our theory, equation (3), predicts that reciprocal motifs will therefore have the strongest impact on the recurrency and dimensionality of the circuit. To quantify this effect, we combined excitatory and inhibitory motif coefficients into effective parameters (Methods and Supplementary Information). In this process, the individual coefficients are weighted according to the relative strength of excitatory and inhibitory synapses. Beyond this weighting, the effective connectivity strengths of synapses within network models depend on the gain of neurons^43,47^ and are therefore subject to changes across time. In particular, the effective strengths measured from in vitro recordings do not necessarily match in vivo settings. We therefore studied a range of different scenarios.

We started with a reference setting, where inhibition is sufficiently strong to balance excitation (Fig. 5c, left). In this scenario, all excitatory and inhibitory motifs enter in determining *R*_motifs_. We find that *R*_motifs_ is then significantly smaller than 1, meaning that motifs stabilize the dynamics, reducing recurrency and, by equation (2), increasing dimensionality. We then consider the two ‘extreme’ scenarios in which either only the excitatory (Fig. 5c, middle) or inhibitory population (Fig. 5c, right) is active, so that the corresponding EE and II motifs are the only ones that influence *R*_motifs_. We find that this leads to *R*_motifs_ > 1 (one-sided *t*-test *P* < 10^−20^), thus leading to the opposite destabilizing effect.

The same was true for motifs within the excitatory population in human cortical circuits (Fig. 5d, one-sided *t*-test *P* < 10^−20^). We further confirmed that this effect is also predicted for previously published data on excitatory connections in the rat visual cortex^32^ (Supplementary Information). The discrepancy between the reference EI scenario and the extreme cases (only E or I) underscores the prominent role of EI motifs, specifically reciprocal EI motifs. These motifs become relevant when both populations are active and counteract the influence of EE and II motifs, leading to the overall decrease in recurrency *R*_motifs_ < 1 (Fig. 5c and Supplementary Fig. 6). Departing from the extreme cases of only excitation or inhibition to more reasonable modulations of effective excitatory and inhibitory strengths away from the reference scenario shows that increased inhibition in general leads to an increase of *R*_motifs_ (Fig. 5e). Increased excitation instead has the opposite effect of decreasing *R*_motifs_.

Recall that the overall recurrency of a circuit is, however, determined not only by motifs but also by the factor *σ*, which in turn depends on the connection probabilities of populations within the mouse primary visual cortex network. A similar weighting of excitatory and inhibitory contributions in relation to the effective strength of the respective synapses applies here (Methods and Supplementary Information). We found that *σ* follows the same trend as *R*_motifs_ when increasing inhibition (Fig. 5f); both *σ* and *R*_motifs_ increase such that the overall recurrency of the circuit increases and the dimensionality decreases. For excitation, however, we found opposing roles of *σ* and *R*_motifs_. While *R*_motifs_ decreases with increasing excitation (Fig. 5e), *σ* increases slightly (Fig. 5f), and this compensation limits the effects of changing excitation on the overall recurrency and dimensionality.

To test these theoretical predictions, we re-evaluated the dimensionality statistics of the HMM states of spontaneous activity in the mouse visual cortex by relating the state-resolved dimensionality to the average firing rate of neural populations. Putative excitatory and inhibitory neurons were distinguished based on spike waveform (Methods). We indeed found that for elevated inhibitory firing and thus effective inhibitory gain, the dimensionality of states tends to decrease (Fig. 5g), in line with our prediction. Also in agreement with our theory, we did not find a systematic trend in dimensionality with excitatory neuron firing rates (Fig. 5h). We argue that this is in part because of the opposing effects of *R*_motifs_ and *σ*; it is also true that excitatory firing rates simply did not change as strongly as inhibitory rates across states, so that the overall changes in excitatory gain are expected to be weaker.

The data in Fig. 5g,h also clearly show that a large spread of dimensionalities (across states and sessions) remains at any given overall firing rate of excitatory and inhibitory cells. This variability is likely due to specific configurations of effective connectivity in the different conditions, above and beyond overall trends to (sub)population averaged firing rates; other effects such as varying external inputs could also contribute. In the analysis above, we only considered a rather coarse classification of cells into excitatory and inhibitory groups. In fact, Neuropixels electrodes record at different cortical depths and in different cortical layers with distinct excitatory and inhibitory populations. We therefore generalized our connectivity analyses to account for layer information, which can be used to obtain more specific results on recurrency (Supplementary Information and Supplementary Fig. 7). Furthermore, it is well known that there are multiple different excitatory and inhibitory neuron subtypes with different dynamical, functional and wiring properties^37,58,59^. In our synaptic physiology data, we indeed had access to inhibitory subtypes and could distinguish between vasoactive intestinal peptide-, somatostatin- and parvalbumin-expressing cells. We performed a similar connectivity analysis on the level of these subtypes and again could identify substantial differences in cell-type-specific connection probabilities and motif abundances (Supplementary Fig. 9). Applying our theory of recurrency to these data showed differential effects of elevated gain of inhibitory subtypes (Supplementary Information and Supplementary Fig. 8), which, like the differences across layers, may contribute to the variability observed in Fig. 5g,h. Optogenetic perturbation techniques, beyond the optotagging available in this dataset, provide a path to future tests of the role of cell-type-specific connectivity in shaping neural covariability and dimensionality.

In sum, our systematic theoretical formalism provides useful tools that link the statistics of connectivity and activity across synaptic to circuit-wide scales.

## Discussion

We showed that neural networks across the mouse cortex operate in a strongly recurrent regime, in which the dimensionality of their activity is strongly constrained. A feature of circuits in this regime is the ability to sensitively modulate the relative dimensionality of their activity patterns via their recurrency *R*, a unifying measure of a network’s overall recurrent coupling strength. We note that related concepts of activity sensitivity have been studied in other close-to-critical dynamical regimes, such as the reverberating regime controlled by excitatory–inhibitory balance and hypothesized to have important consequences for computation^60^. With this said, while we argue that the dimensionality of activity in the networks that we sample is low relative to the number of neurons in local circuits, it is not low in absolute terms, still numbering in (at least) the hundreds. Moreover, we emphasize that, while we measure dimensionality using a commonly used metric ^3,4,11,13,40^ and show that similar trends occur for another popular metric (Methods and Extended Data Fig. 4), the same will not necessarily be true for all valid and interesting measures of dimensionality, such as those that count numbers of significant modes without explicitly weighting them via their explained variance (for example, the recent work of Manley et al.^44^).

Our theoretical work links observations on dimensionality to predictions for network recurrency *R*: a higher dimensionality suggests a lower recurrency and vice versa. We showed that the summary covariance statistics *m*^2^ + *s*^2^ and *v*^2^ / *N* displayed in Fig. 2d–f partition the space of networks into regions of low and high recurrency and may thus serve as metrics to assess this quantity from experimental data. Moreover, we showed that the critical circuit features that determine a circuit’s recurrency *R* are not only its overall connection strength but also a tractable set of local synaptic motifs. We use theoretical tools to quantify the effect of these motifs via a compact index *R*_motifs_. This provides a concrete target quantity that can, as we show, be readily measured from emerging, large-scale synaptic connectivity datasets and used to test predictions about the role of synaptic structure in controlling dimensionality. We show how the engagement of different neural populations form one avenue for controlling dimensionality via local connectivity statistics. This is, of course, the tip of the iceberg in terms of how cortical circuitry and dynamics is modulated across time and state, and allied effects such as modulations in external inputs and activity heterogeneity within populations^45^ are likely prominent.

As we reviewed above, high-dimensional activity can retain stimulus details, while lower-dimensional activity can promote robust and general downstream decoding; together, this points to new functional roles for modulatory and adaptive mechanisms that may take effect across time and brain states. In sum, our theory and brain-wide experimental analyses converge to provide new evidence for an intriguing concept^53,61,62^: that the connectivity of cortical brain networks exerts control over their activity in a highly tractable manner via the building blocks of their local circuitry.

## Methods

### Electrophysiology data and analysis

#### Data description summary

Data are publicly available, and a detailed description has been made available by the Allen Institute for Brain Science^25,26^. The Allen Institute for Brain Science Institutional Animal Care and Use Committee authorized guidelines for mice kept in the animal facility. After surgery, all mice were single housed and kept on a reverse 12-h light cycle in a shared facility at 20−22 °C with 30–70% humidity. Experiments were performed during the dark cycle. Mice had free access to food and water throughout the experiments.

Each mouse had a grade 5 titanium headframe with a cranial window to facilitate co-registration across surgical, intrinsic signal imaging and electrophysiology rigs. The headframe was glued to a black acrylic photopolymer well that shielded the craniotomy and probes during the experiment and provided a surface for precisely aligning the insertion window.

Mice were mildly anesthetized with 1–1.4% isoflurane and monitored with a PhysioSuite (model PS-MSTAT-RT). Eye drops (Lacri99 Lube Lubricant Eye Ointment, Refresh) kept eyes hydrated and clear during anesthesia. The stimulus we analyzed was a mean brightness gray background and was part of a longer stimulus train (see Siegle et al.^26^ for details). Mice spent 2 weeks habituating in sound-attenuated training boxes with headframe holders, running wheels and stimulus monitors.

Recordings contained 58 experimental sessions in adult mice. At the beginning of the experimental session, the cranial coverslip was replaced with an insertion window containing holes aligned to six cortical visual areas. Neural recordings were performed with six Neuropixels probes each containing 960 recording sites, providing a maximum of 3.84 mm of tissue coverage. Visual stimuli were generated using scripts based on PsychoPy and followed one of two stimulus sequences (‘brain observatory 1.1’ and ‘functional connectivity’).

We focused on spontaneous neural activity registered while the animal was not performing any task. In each session, the spontaneous activity condition lasted 30 min while the animal was in front of a screen of mean gray luminance. We therefore analyzed 26 of the original 58 sessions corresponding to the ‘functional connectivity’ subdataset as they included such periods of spontaneous activity. To perform the analysis, we used and extended the Allen SDK toolbox available at https://github.com/AllenInstitute/AllenSDK.

The fit and analysis of HMM states were performed on the neural activity binned into 5-ms windows, while remaining analyses, such as the computation of dimensionality, was performed on neural activity binned into 100 ms; see further details below (Supplementary Fig. 1). Across all analyses, we only used those recordings where at least 30 neurons for a specific brain region or brain area were simultaneously recorded. In addition, layer and area labels for each cortical unit (layers: 1, 2/3, 4, 5 or 6; areas: primary visual cortex, rostrolateral visual cortex, lateral visual cortex, anterolateral visual cortex, posteromedial visual cortex and anteromedial visual cortex) were assigned to each neuron using CCFv3 coordinates. The relative thickness of each layer, which can vary both within and between areas, is based on the average of the 1,675 individual brains used to generate the volume template^26^.

Neurons were classified as putative excitatory or putative inhibitory based on the waveform duration. Waveform durations were analyzed across all recorded neurons. A Gaussian mixture model was fit to the distribution, and neurons belonging to either the slow or fast distribution with a *P* < 0.05 were classified as putative excitatory or inhibitory neurons, respectively. Neurons that did not meet such requirements were not classified as either.

#### HMM analysis

The HMM analysis of spontaneous activity followed a two-stage procedure and was performed using the ssm toolbox (https://github.com/lindermanlab/ssm). First, we binned spike trains into 5-ms intervals across the 30 min of neural recordings for each session. We then estimated the number of hidden states using a fivefold cross-validation procedure. We fit a cross-validated HMM and computed the log-likelihood of fit for an increasing number of hidden states (from 2 to 21). Using the kneed toolkit (https://github.com/arvkevi/kneed), we chose the number of states by means of detecting an elbow in the average log-likelihood across number of states. This algorithm selects a cross-validation curve point on the basis of a single parameter, *s*. We used the default value *s* = 1. Using the resulting number of states, we then fit an HMM to the spike counts *n* times (*n* = 5) and selected the fit with maximum log-likelihood across these. This is to guarantee that the final fit is among the best possible for the selected number of states. The output of the HMM analysis was a confidence (a posterior probability between 0 and 100%) for each of the underlying hidden factors to have generated the neural activity in each bin. We thresholded this confidence (to 80%) to select only temporal intervals in which the algorithm identified a specific hidden factor as the cause of the collective neural activity. Once all time points, and consequently spike-count population vectors, were assigned to one or no hidden state (if no state passed the confidence threshold), we used all such vectors assigned to the same state to calculate a state-specific covariance capturing neural variability for each state. To compute the covariance matrix of neural population vectors, our reference bin size is 100 ms, whereas the HMM analysis was performed on 5-ms windows of activity. Before calculating the covariance matrix, we therefore rebinned neural activity within each state into 100-ms windows, discarding all appearances of an individual state that lasted less than 100 ms. Extended Data Fig. 1 provides further details on the results of the HMM analysis.

#### Covariance and dimensionality analysis

##### Participation ratio

We analyzed the participation ratio *D*_PR_ (refs. ^3,4,11,13,40^) as a measure of dimensionality (Extended Data Fig. 2). The participation ratio is defined as

4 $${D}_{{\rm{PR}}}=\frac{1}{\mathop{\sum }\nolimits_{i = 1}^{N}{\tilde{\lambda }}_{i}^{2}}$$

with normalized eigenvalues ${\tilde{\lambda }}_{i}={\lambda }_{i}/\mathop{\sum }\nolimits_{k = 1}^{N}{\lambda }_{k}$ of the covariance matrix *C* between *N* neurons. The eigenvalues of the covariance matrix are related to the trace of the matrix, such that this measure can be rewritten in terms of the means $\bar{a}$ and $\bar{c}$ and standard deviations *δa* and *δc* of variances and covariances, respectively (Extended Data Fig. 2), and the number of neurons recorded *N* (similar to ref. ^13^):

5 $${D}_{{\rm{PR}}}=\frac{N}{1+{\left(\frac{\delta a}{\bar{a}}\right)}^{2}+(N-1)\left({\left(\frac{\delta c}{\bar{a}}\right)}^{2}+{\left(\frac{\bar{c}}{\bar{a}}\right)}^{2}\right)}.$$

For convenience, in the main text, we defined the rescaled statistics of covariances $v=\delta a/\bar{a}$, $m=\bar{c}/\bar{a}$ and $v=\delta c/\bar{a}$.

##### Subsampling and dimensionality extrapolation

In most experiments, including those considered here, one only records from a subset of neurons sampled from a given neural network. Rewriting the participation ratio in terms of the statistics of covariances reveals, due to the explicit factor *N* in equation (5), that the dimensionality *D*_PR_ of the recorded activity depends on the number of experimentally recorded neurons *N*. It therefore does not immediately reflect the true dimensionality of the underlying network, which we take to be of size N^*^. The statistics of covariances on which *D*_PR_ is based are derived from a covariance matrix that is likewise subsampled. In the absence of any bias in the subsampling procedure, so that the statistics of covariances are invariant with respect to how many neurons are recorded within the network, one can recover the dimensionality *D*_PR_ of the full network dynamics by replacing *N* with the assumed underlying network size N^*^ in equation (5), thus extrapolating to this assumed size (as shown in Figs. 1–3 and Extended Data Fig. 4). By taking multiple random subsamples of the experimental data, we confirmed that there is indeed no systematic trend in the statistics of covariances with *N* (Extended Data Fig. 3).

We noted that covariance statistics were roughly similar both locally around a given Neuropixels probe (Extended Data Fig. 3e–g) and across multiple probes up to hundreds of microns apart (Extended Data Fig. 3a–c,i–k and Fig. 1g,h). To be conservative in making our claim of a low relative dimensionality, we chose a lower bound number of N^*^ = 10^4^ for the assumed underlying local network size. While we simply wish to take a conservative lower bound number, for reference, we note that 10^4^ roughly corresponds to the number of neurons in a cylinder of 150−200 μm (using a neuron density of 155,000 per mm^3^ and a thickness of height 0.8 mm, corresponding to the visual cortex^63^).

Keeping the statistics of covariances fixed during extrapolation yields, by definition, a saturating dimensionality for *N* → *∞*. Whether this saturation occurs already below the actual network size N^*^ depends on the statistics of covariances as shown in model simulations in Fig. 2 and Extended Data Fig. 4, where the dimensionality can be not only extrapolated from a subsample of neurons but also measured for any *N* ≤ N^*^. For fixed covariance statistics, the slope of the dimensionality extrapolation curve can be predicted by taking the derivative of equation (5) with respect to *N*, $\frac{d{D}_{{\rm{PR}}}}{dN}$. In Fig. 2e, we compare this slope at N^*^ = 10^4^ to the value of the slope at experimentally accessible subsample sizes *N* = 275 in our electrophysiological recordings. This analysis shows that the slope largely decreases for larger *N* across all sessions and states. This is also true when subdividing the data into individual visual cortical areas (Extended Data Fig. 6).

Note that estimators for the (cross-neuron) variances of spike variances and covariances are biased when they are based on a finite number of trials. In this case, the estimators also receive a correction that depends on the number of recorded neurons. These biases can, however, be removed as noted below and in the Supplementary Information. This bias correction is essential to obtain the correct extrapolation behavior, as an estimate of *s* biased to larger values due to noisy covariance estimators will incorrectly lead to early saturation of dimensionality with network size.

In addition to the verification of the dimensionality extrapolation on model data (Fig. 2b), we validated the procedure on two-photon calcium imaging data that allowed us to access simultaneously recorded activity of up to 10,000 or more neurons (Fig. 2c and Extended Data Fig. 5).

##### Comparison to other measures of dimensionality

**Extended Data Fig. 9:**
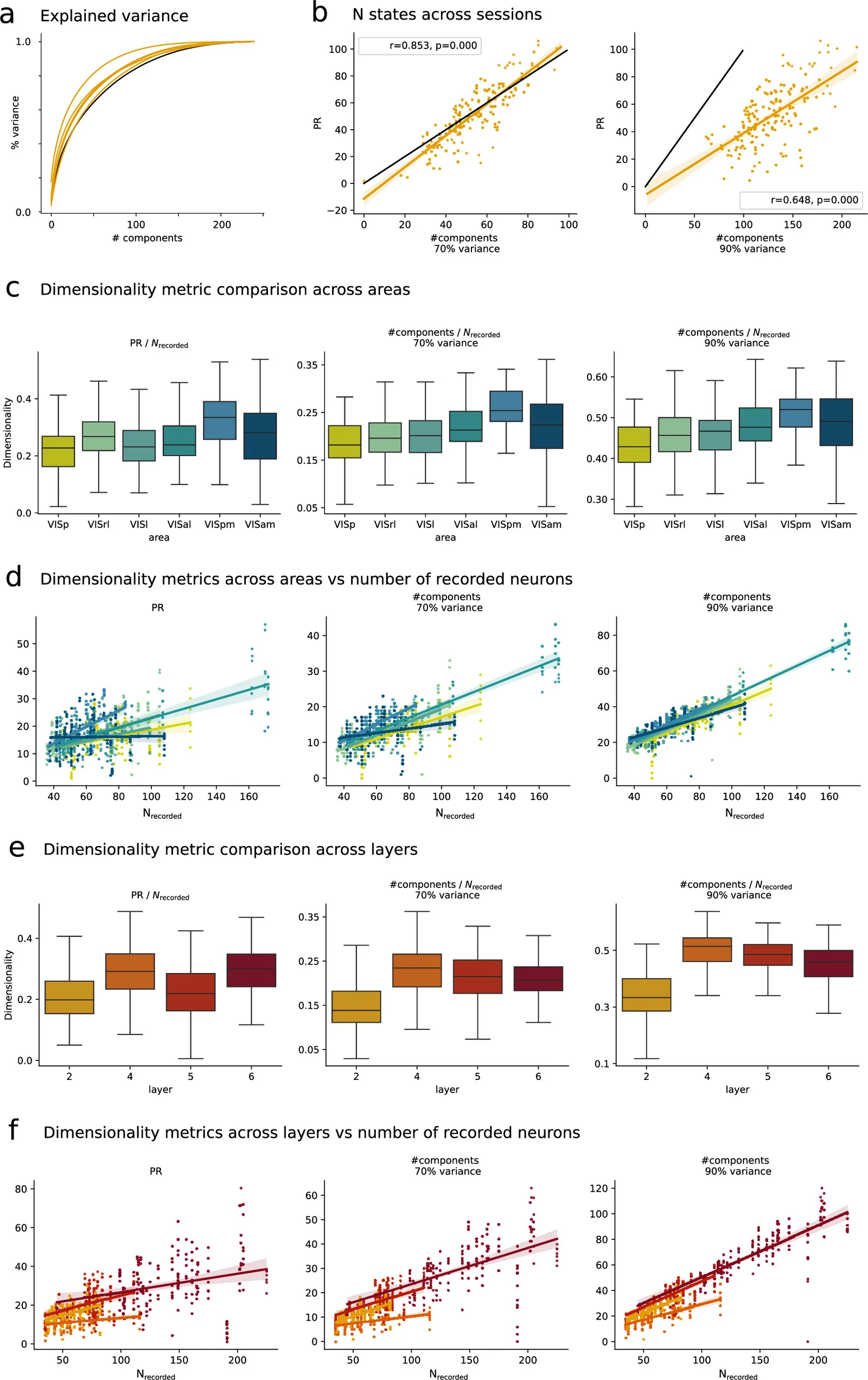
Dimensionality comparison. This figure compares our results using the participation ratio measure of dimensionality, with results based on other measures of dimensionality. The participation ratio measure is shown to correlate well with a measure based on the number of components required to explain 70% of the variance of neural activity. **a**) Curves of variance explained for different HMM states in one example session. The participation ratio ${D}_{{\rm{PR}}}=1/{\sum }_{i}{\tilde{\lambda }}_{i}^{2}$ depends on all values of the curve (rescaled covariance eigenvalues ${\tilde{\lambda }}_{i}={\lambda }_{i}/{\sum }_{k}{\lambda }_{k}$). **b**) Left: Participation ratio vs number of components required to explain 70% variance. Right: Participation ratio vs number of components required to explain 90% variance. **c**) Comparison of area-resolved dimensionality metrics. Same metrics as introduced in panel **b**. **d**) Dependence of dimensionality metrics on the number of recorded neurons (area resolved). **e**) Same as panel d realized across layers. **f**) Same as panel d realized across layers. Boxes show median and IQR, whiskers show 1.5 × IQR. Same n-values as in Fig. 3d top.

The participation ratio is a measure of dimensionality that allows for analytical study of its dependence on the number of recorded neurons. Other well-established measures of dimensionality are based on curves of variance explained. Extended Data Fig. 9 compares our results using the participation ratio with results based on these other measures. The participation ratio is shown to correlate well with a measure based on the number of components required to explain 70% of the variance of neural activity. Additionally, the measures based on variance explained show the same strong dependence on the number of recorded neurons as the (non-extrapolated) participation ratio. As shown in Extended Data Fig. 3, this dependence on the number of recorded neurons can be removed for the participation ratio measure via extrapolation. The extrapolated value does not show a systematic dependence on the number of recorded neurons (Extended Data Fig. 3d,h,l). Such extrapolation is, however, not possible for the other measures of dimensionality, where an analytical expression for the *N* dependence is not available.

Furthermore, in simulations of network models, where we have access to all neuron activities, we tested whether the similarity between the participation ratio and dimensionality based on 70% explained variance still holds beyond the experimentally accessible range of number of recorded neurons. We simulated networks of N^*^ = 10^4^ neurons and subsampled them across the whole range 75 < *N* ≤ N^*^. Extended Data Fig. 4 shows that the behavior of the 70% explained variance measure is qualitatively fully analogous to the behavior of the participation ratio (see also Fig. 2b). For networks with strong recurrency, the dimensionality curve saturates at low values relative to the network size, while networks with weak recurrency keep on showing an almost linear growth in dimensionality with *N* up to values of the full network size. The differences in the dimensionality behavior across networks are thus consistently captured across different dimensionality metrics, with the participation ratio having the advantage that this behavior can be predicted by extrapolation from small subsample sizes.

We therefore conclude that the participation ratio and its extrapolation are an appropriate and more powerful method for comparing distinct sessions, regions and layers, as shown in Figs. 1 and 3 of the main text.

##### Dependence on bin sizes

We finally tested the robustness of our dimensionality results with respect to different bin size choices. Extended Data Fig. 7 shows the dependence of dimensionality (extrapolated participation ratio) on the sizes of time bins in which spikes are counted across different visual areas and cortical layers. The results are generally consistent across the tested bin sizes of 50, 100 (reference value) and 200 ms.

#### Bias correction in the statistics of covariances

We performed a theoretical analysis of the bias on the covariance statistics induced by subsampling both neurons and trials (see also Supplementary Information and ref. ^13^ for a similar analysis): the empirical estimates $\hat{\overline{a}}$ and $\hat{\overline{c}}$ of the average variances and covariances are unbiased, while the variances $\hat{\delta {a}^{2}}$ and $\hat{\delta {c}^{2}}$ of both variances and covariances have a bias that decays with the number of trials *N*_*T*_ as $\sim \frac{1}{{N}_{T}}$:

6 $$\overline{a}=\hat{\overline{a}}$$

7 $$\overline{c}=\hat{\overline{c}}$$

8 $$\delta {a}^{2}=\frac{{N}_{T}-1}{{N}_{T}+1}\hat{\delta {a}^{2}}-\frac{2\left({\hat{\overline{a}}}^{2}-{\hat{\overline{c}}}^{2}\right)}{{N}_{T}+1}+\frac{2\delta {c}^{2}}{{N}_{T}+1}$$

9 $$\delta {c}^{2}=\frac{{N}_{T}-1}{{N}_{T}}\hat{\delta {c}^{2}}-\frac{{\hat{\overline{a}}}^{2}-{\hat{\overline{c}}}^{2}}{{N}_{T}}-\frac{4}{N+1}\frac{{\hat{\overline{c}}}^{2}-\hat{\overline{a}}\,\hat{\overline{c}}}{{N}_{T}}$$

Here, $\hat{\cdot }$ indicates the empirical estimate, and the non-hat quantities indicate the true values. Based on such an analysis, we performed a bias correction (Supplementary Fig. 1 and Methods).

#### Dimensionality analysis and HMM states

The HMM analysis mapped intervals in the spontaneous activity to a number of hidden latent states whose appearance often corresponded to changes in the behavior of the animal (Fig. 1b,c). We then compared the covariance statistics obtained separately in each state to the covariance statistics obtained in the entire interval of spontaneous activity (Fig. 1g–j). Also, as a cross-check, we compared these two conditions to the one where for each neural state we performed a cross-validated LFA over the covariance statistics (Supplementary Fig. 2). This removed any remaining non-stationary component potentially not detected by the HMM.

In Fig. 3d, we computed the dimensionality change across layers and brain areas. This is defined as $\Delta {D}_{{\rm{PR}}}=\frac{2| {D}_{{\rm{PR}}}(A)-{D}_{{\rm{PR}}}(B)| }{{D}_{{\rm{PR}}}(A)+{D}_{{\rm{PR}}}(B)}$ where *A*, *B* are two separate conditions with distinct activity statistics. In all these analyses, these two conditions correspond to a different neural state. In each recording session, we took all possible combinations of two HMM states. We computed *D*_PR_ in each of the two, arbitrarily assigning one state to condition *A* and the second to condition *B* (notice that Δ*D*_PR_ is symmetric in *A*, *B*). We then computed Δ*D*_PR_ averaging across all states and recording sessions. The only difference across panels and bars in Fig. 3d is that the computation of dimensionality was restricted to only those neurons of a given layer or brain area. In the control condition, we split the statistics of states into two parts, the first and second half, and used these two as conditions *A*, *B*.

### Two-photon calcium imaging

#### Experiment and recording setup

All two-photon experiments were performed in accordance with the German Animal Protection Law and local ethics committee (LAVE, NRW). Recordings were done in two 24-week-old transgenic mice expressing the calcium indicator GCamP6s in all excitatory pyramidal neurons in the cortex (tetO-GCaMP6s (The Jackson Laboratory, 024742) crossed with *Camk2a*-tTA mice (The Jackson Laboratory, 007004)).

Detailed surgical procedures are described elsewhere^64^. In short, analgesic treatment was done through a combination of carprofen (4 mg per kg (body weight)) and buprenorphine (0.1 mg per kg (body weight)), and mice were anesthetized using 1–2.5% isoflurane in oxygen. A 3.5-mm-wide craniotomy was performed over the frontal anterior lateral motor cortex, and a 3-mm circular cover slip was placed over the dura. The cover slip was fixed with light-curable dental cement (Tetric Evoflow, Nordenta), and a metal head bar was attached to the skull, using a combination of quick adhesive cement (Superbond C&B, Sun Medical) and fast-curing acrylic resin (Jet Denture repair, Lang Dental). After surgery, animals were kept on a heating blanket and received additional analgesic treatment during the subsequent recovery period.

Two-photon imaging was performed in head-fixed mice using a custom-built setup with a commercial galvo-resonant scanning two-photon microscope (Bergamo II, Thorlabs) and a femtosecond-pulsed Ti:Sapphire laser tuned to 920 nm (Chameleon Vision-S, Coherent). Images were acquired through a ×16/0.8-NA Nikon water-immersion objective (CFI75 LWD 16X W) at a resolution of 512 × 512 pixels, corresponding to a field of view of 700 μm × 700 μm, using ScanImage 2020 (ref. ^65^) for data acquisition. To increase neuron yield (2,689–13,399 neurons across recordings), we performed multiplane imaging with a piezo stage that continuously moved the objective over a range of 250–550 μm from the cortical surface. In three recordings, we collected 10 imaging planes spaced 30 μm apart, with a volumetric sampling rate of 3 Hz, and in two recordings we collected 15 imaging planes spaced 20 μm apart, with a sampling rate of 1.87 Hz. Each recording lasted 30 min. In addition, two video cameras (BFS-U3-16S2M-CS, FLIR) simultaneously captured animal face and body movements. Video frames were acquired at 60 Hz using the labcams software package (v0.2, https://github.com/jcouto/labcams) by J. Couto and were synchronized to the neural imaging data through periodic trigger signals.

#### Data processing

Raw two-photon images were processed using Suite2P^66^ to perform motion correction, model-based region of interest detection, correction for neuropil contamination and spike deconvolution. To account for different expression levels in neurons and systematic trends of fluorescence strength with cortical depth, we normalized inferred-spike signals of each neuron by their respective median fluorescence across time^67^. To remove noise, we only considered neurons above the fifth percentile of the median fluorescence distribution across all neurons and then performed the same covariance and dimensionality analysis as in the electrophysiological recordings.

To segment the imaging data into movement and rest states, we used DeepLabCut^68^ to track the motion of multiple facial and body landmarks. For each marker, we computed a motion-energy signal defined as the time derivative of the absolute value of the Hilbert transform of its position trace. We then reduced the dimensionality of the resulting marker-wise motion signals using principal component analysis and extracted the first principal component as a common movement vector capturing the dominant pattern of animal motion over time. This movement vector was resampled to match the sampling rate of the neural imaging data, and movement epochs were defined as periods in which the movement signal exceeded 2 s.d. above the median. According to this criterion, mice were predominantly at rest: across sessions, 11 ± 5% of time bins were classified as movement epochs.

### Theoretical and computational analysis

#### Network models and linear response theory

We made use of the fact that correlations in spontaneous, asynchronous irregular activity states of spiking networks can be well understood using linear response theory^30,69^: starting from a network of leaky integrate-and-fire neurons, linearization around some stationary state maps the statistics of fluctuations to an equivalent set of Ornstein–Uhlenbeck processes coupled via some effective connectivity matrix^70,71^. The stationarity required for linearization holds if population-level fluctuations are not strongly amplified by large excess excitation. In our analysis of experimental data, we ensure stationarity by studying covariances and dimensionality within individual HMM states.

Ornstein–Uhlenbeck processes are linear stochastic differential equations that can be analyzed using statistical field theory^18,72^. Figure 3b shows that such theory faithfully predicts the statistics of covariances and dimensionality as a function of the recurrency in direct simulations of leaky integrate-and-fire neurons.

##### Recurrency (spectral radius of effective connectivity)

The recurrency *R* is a summary measure of the network’s effective connectivity and formally corresponds to the spectral radius of bulk connectivity eigenvalues (Extended Data Fig. 8 and Supplementary Information). Our theoretical derivations first focused on networks with homogeneous connection statistics. We then expanded this theoretical analysis to investigate networks with excitatory and (multiple) inhibitory populations and distinct connection statistics for the different types of neurons (Supplementary Information). Our results linking the dimensionality and the spectral radius also generalize well to more complex network topologies (Fig. 3b).

#### Theory of recurrency in networks with second-order motifs

Using the path-integral representation of coupled Ornstein–Uhlenbeck processes^72^, we performed an average of the moment-generating function for the network dynamics over the statistics of connections. Second-order connection motifs were thereby incorporated via the covariance tensor Δ_*ijkl*_ = 〈*W*_*ik*_*W*_*jl*_〉 − 〈*W*_*ik*_〉〈*W*_*jl*_〉 between connections *W*_*ik*_ from neuron *k* to neuron *i* and *W*_*jl*_ from neuron *l* to neuron *j*. Similar to the case of reciprocal connections^51,73^, the second-order connectivity statistics yield non-Gaussian integrals that cannot be solved exactly. We obtained good approximations to these integrals for large networks by using a saddle-point approximation of auxiliary fields that were introduced for the terms related to the various motif contributions. The associated self-consistency equations for the saddle points showed parameter-dependent divergence structures that we related to connectivity eigenvalues crossing the line of instability of the linear network. By distinguishing between outlier and bulk eigenvalues, this analysis allowed us to infer a theoretical prediction of the spectral radius of the effective connectivity in relation to the various motif abundances (Supplementary Information).

#### Numerical validation of motifs theory

Following Hu et al.^62^, we numerically generated network connectivity matrices as a superposition of Gaussian independent and identically distributed matrices

10 $${W}_{ij}=\mu +A{\nu }_{ij}+B{\nu }_{ji}+C{\eta }_{i}+D{\eta }_{j}\,.$$

In the Supplementary Information, we show that coefficients *A*, *B*, *C*, *D* can be chosen such that the second-order statistics of *W* reflect the correlations induced by various motif abundances in sparse networks. Note that this Gaussian connectivity does not contain any higher-order cumulant information of networks with motifs. The effect of such higher-order cumulants is, however, typically suppressed by the large network size of biological networks, as discussed in the Supplementary Information. It turns out that equation (10) cannot generate any arbitrary constellation of motif abundances, as detailed in the Supplementary Information. In particular, correlations induced by divergent and convergent motifs are typically positive, and chain motif correlations cannot be generated independently from other motifs. These considerations give rise to the *τ* ranges shown in Fig. 4, where we validated our theoretical predictions for the spectral radius and dimensionality.

The spectral radius is well predicted for all values of *τ* (Fig. 4a). The same holds true for the prediction of the dimensionality (Fig. 4b), except for reciprocal motifs, where the prediction is only correct on a qualitative level. The prediction of the dimensionality relies, in addition to our results on the motif dependence of the spectral radius, on the mapping between the spectral radius and the width of the covariance distribution that was derived in ref. ^18^ for homogeneous random networks using beyond-mean-field techniques. This relation is robust as long as eigenvalue spectra of connectivities show a circular organization in the complex plane. Convergent, divergent and chain motifs do not strongly alter the shape of the bulk of connectivity eigenvalues^34^. Therefore, the theory for homogeneous random networks yields correct quantitative results for these cases. Reciprocal motifs, however, deform the bulk eigenvalues from a circular to an elliptic shape^74^, which causes the minor quantitative mismatch between theory and simulations.

#### Motif coefficients in multipopulation networks

In networks with multiple populations and population-specific connectivity statistics, the latter statistics are combined to form the overall contributions *σ* and *R*_motifs_ that shape the recurrency. While the absolute strength of synapses does not enter the motif coefficients *τ* and therefore *R*_motifs_, their relative strength does. Our theoretical analysis, detailed in the Supplementary Information, yields the following expressions:

$$\begin{array}{ll}{\sigma }^{2}= \displaystyle\frac{{N}_{E}}{N}{\rho }_{E}^{2}{J}^{2}{\hat{\sigma }}_{E}^{2}+\displaystyle\frac{{N}_{I}}{N}{\rho }_{I}^{2}{g}^{2}{J}^{2}{\hat{\sigma }}_{I}^{2}\,,\\ {\tau }_{\mathrm{div}} = \displaystyle\frac{{\rho }_{E}^{2}{\hat{\sigma }}_{E}^{2}{\tau }_{\mathrm{div},E}+\gamma {\rho }_{I}^{2}{g}^{2}{\hat{\sigma }}_{I}^{2}{\tau }_{\mathrm{div},I}}{{\rho }_{E}^{2}{\hat{\sigma }}_{E}^{2}+\gamma {\rho }_{I}^{2}{g}^{2}{\hat{\sigma }}_{I}^{2}}\,,\\ {\tau }_{\mathrm{con}}=\displaystyle\frac{{\rho }_{E}^{2}{\hat{\sigma }}_{E}^{2}{\tau }_{\mathrm{con},EE}+\gamma {g}^{2}{\rho }_{I}^{2}{\hat{\sigma }}_{I}^{2}{\tau }_{\mathrm{con},II}}{{\rho }_{E}^{2}{\hat{\sigma }}_{E}^{2}+\gamma {\rho }_{I}^{2}{g}^{2}{\hat{\sigma }}_{I}^{2}}\,,\\ {\tau }_{\mathrm{rec}} =\displaystyle\frac{1}{2}\displaystyle\frac{{\rho }_{E}^{2}{\hat{\sigma }}_{E}^{2}{\tau }_{\mathrm{rec},EE}+\gamma {\rho }_{I}^{2}{g}^{2}{\hat{\sigma }}_{I}^{2}{\tau }_{\mathrm{rec},II}}{{\rho }_{E}^{2}{\hat{\sigma }}_{E}^{2}+\gamma {\rho }_{I}^{2}{g}^{2}{\hat{\sigma }}_{I}^{2}}\\\qquad-\displaystyle\frac{1}{2}\displaystyle\frac{(1+\gamma )g{\rho }_{E}{\rho }_{I}\sqrt{{\hat{\sigma }}_{E}^{2}{\hat{\sigma }}_{I}^{2}}{\tau }_{\mathrm{rec},EI}}{{\rho }_{E}^{2}{\hat{\sigma }}_{E}^{2}+\gamma {\rho }_{I}^{2}{g}^{2}{\hat{\sigma }}_{I}^{2}}\,,\\ {\tau }_{\mathrm{chn}} = \displaystyle\frac{1}{2}\displaystyle\frac{{\rho }_{E}^{2}{\hat{\sigma }}_{E}^{2}{\tau }_{\mathrm{chn},EE}+\gamma {\rho }_{I}^{2}{g}^{2}{\hat{\sigma }}_{I}^{2}{\tau }_{\mathrm{chn},II}}{{\rho }_{E}^{2}{\hat{\sigma }}_{E}^{2}+\gamma {\rho }_{I}^{2}{g}^{2}{\hat{\sigma }}_{I}^{2}}\,\mathrm{and}\\\qquad-\displaystyle\frac{1}{2}\displaystyle\frac{\displaystyle\frac{1+\gamma }{2}g{\rho }_{E}{\rho }_{I}\sqrt{{\hat{\sigma }}_{E}^{2}{\hat{\sigma }}_{I}^{2}}({\tau }_{\mathrm{chn},EI}+{\tau }_{\mathrm{chn},IE})}{{\rho }_{E}^{2}{\hat{\sigma }}_{E}^{2}+\gamma {\rho }_{I}^{2}{g}^{2}{\hat{\sigma }}_{I}^{2}},\end{array}$$

with ${\hat{\sigma }}_{E}^{2}={p}_{E}(1-{p}_{E})$ and ${\hat{\sigma }}_{I}^{2}={p}_{I}(1-{p}_{I})$ determined by excitatory and inhibitory connection probabilities *p*_E_ and *p*_I_, respectively. The parameters *γ* and *g* determine the relative size of the inhibitory to the excitatory populations and the relative effective strength of inhibition to excitation. In the reference case, these parameters are set to common values^75^ *γ* = *NI* / *NE* = 0.25 and *g* = 4 (Fig. 5), and they are varied in Supplementary Fig. 10. *ρ*_E_ and *ρ*_I_ denote scaling factors, which by default are set to 1 and only varied in Fig. 5e,f to increase the relative strength of excitation or inhibition, respectively.

The above coefficients ${\tau }_{{\rm{div}}},{\tau }_{{\rm{con}}},{\tau }_{{\rm{rec}}},{\tau }_{{\rm{chn}}}$ are then combined according to equation (3) to yield *R*_motifs_. The latter quantity, like *σ*, thus also depends on the relative strength of excitation and inhibition and the neuronal activity state.

#### Fitting network models to experimental data and inferring recurrency

For the comparison of experimental data with network models in Fig. 2, we used a fitting procedure that is based on our results that link the statistics of covariances *v*, *m* and *s* to the statistics of connectivity and external inputs of our network model. The equations and procedure are detailed in the Supplementary Information. We found that the fit network model (Fig. 2b, purple) needs to operate in the strongly recurrent regime to reproduce the observed statistics of variances and covariances. In particular, the large value of *s* in the experiment could only be produced by strong recurrency (Supplementary Fig. 4). This was emphasized by the comparison to a modified network (Fig. 2b, green) where we reduced the recurrency. A priori, this reduction affected all covariance statistics. For *v* and *m*, the effect of the reduction in recurrency was, however, simply compensated by increasing the strength of uncorrelated external input $\bar{D}\to \bar{D}\frac{1-{R}_{{\rm{weak}}}^{2}}{1-{R}_{{\rm{strong}}}^{2}}$ (Supplementary Eqs. (12) and (13)), where *R*_strong_ ≈ 0.82 is the recurrency of the fit strongly recurrent network and *R*_weak_ = 0.1 is the recurrency of the modified weakly recurrent network. Therefore, both models reproduced the experimentally observed *v* and *m*, but *s* largely differed between the models, leading to the distinct behavior of dimensionality at large *N* (Fig. 2b and Extended Data Fig. 4).

Supplementary Eqs. (12)–(15) were likewise used to map the space of covariances in Fig. 2f in terms of recurrency. Because *m* ≪ *s* in the experimental data and models, the mean recurrent connectivity can be neglected, *μ* ≈ 0, and Supplementary Eqs. (12)–(15) can be simply solved for *R* to obtain the recurrency as a function of *v*^2^ and *m*^2^ + *s*^2^ ≈ *s*^2^. Compared to *R*(*s*^2^) in Supplementary equation (10), which neglects the influence of *v*^2^ on the dimensionality and leads to the simple relation equation (2) between dimensionality and recurrency, the refined relation *R*(*m*^2^ + *s*^2^, *v*^2^) between recurrency and covariance statistics accounts for substantial variability in firing rates across neurons, as reflected by a large *v*^2^ in the experimental data and modeled as a large variance of intrinsic stochasticity across neurons in Supplementary Eqs. (14) and (15).

### Synaptic physiology data and analysis

#### Data description summary

We analyzed recently published data collected at the Allen Institute for Brain Science^37^, where synaptic connections were probed using simultaneous patch clamp of three- to eight-cell groups. The dataset reports the synapses (or lack thereof) between any pair of probed neurons. Over 32,000 synaptic connections were probed, resulting in 1,368 chemical synapses from mouse primary visual cortex and 363 from human cortex.

Multiple methodologies were used to identify each subclass of cell based on cell type and layer position. Brightfield, epifluorescence (tdTomato and enhanced green fluorescent protein) and dye-filled recording pipettes were used to acquire images of the recording site. By combining epifluorescence and pipette dye, transgenic expression in cells was then identified. The genetic identification of each cell was complemented or supplemented by a morphological examination (see ref. ^37^ for details).

We primarily analyzed the data accounting for the excitatory or inhibitory nature of the synapses (Fig. 5). In the Supplementary Information, we furthermore distinguished between different layers and inhibitory cell types of pre- and postsynaptic neurons (layer 2 versus layer 5, somatostatin- versus vasoactive intestinal peptide- versus parvalbumin-expressing cells; Supplementary Figs. 7–10).

#### Extraction of connection probabilities and motif statistics from synaptic physiology data

To estimate *R*_motifs_ from the whole dataset or a subset of data (bootstraps), we proceeded as follows. Each motif type considered in this work consists of two connections. In our theoretical work, we, for simplicity, only keep track of the presynaptic cell type of each connection (Supplementary Information). Divergent motifs are thus computed per presynaptic cell type, irrespective of the two postsynaptic cell types. Convergent motifs take into account the identity of both presynaptic cells. As the postsynaptic neuron of one connection in a reciprocal motif is the presynaptic neuron of the other connection, our analysis includes the identity of pre- and postsynaptic neurons. For chain motifs, we take into account both presynaptic neurons, which implies that only the final neuron of the chain is left unspecified. To obtain consistency between our theory and data analysis, we therefore also calculated motif probabilities from the data on this level of granularity.

We first computed the probabilities *p*_*α*_ of having an excitatory (*α* = *E*) or inhibitory (*α* = *I*) synapse among two neurons (combination of values in each row of Fig. 5a) and estimated the variances ${\sigma }_{\alpha }^{2}={p}_{\alpha }(1-{p}_{\alpha })$ according to Bernoulli statistics. We accumulated information across sessions of *n* = 8 or fewer neurons by summing the number of observed connections in all these sessions and dividing by the summed number of possible connections in all these sessions. In addition, for reciprocal and chain motifs, we calculated probabilities *p*_*βα*_ of having a connection between a presynaptic neuron of type *α* and postsynaptic neuron of type *β* (Fig. 5a), along with the respective variances. These probabilities (first-order statistics) are required to normalize the motif probabilities (second-order statistics) to determine whether there is an over- or underrepresentation of motifs compared to an assumption of independent connectivity statistics.

We then computed the probabilities of having a reciprocal, chain, convergent or divergent motif in the data. In each session where *n* (eight or fewer) neurons were probed, we computed the total number of pairs (reciprocal motifs) or triplets of neurons (divergent, convergent, chain motifs) that could carry a corresponding motif. For example, for *n* = 8 excitatory neurons, the number of possible excitatory reciprocal motifs is 8 × 7 / 2 = 28. We then counted the number of motifs in each session of each type, taking into account the identity of presynaptic neurons as described above. Finally, across all sessions, we summed up all the occurrences for each motif type and the total number of pairs or triplets of neurons that could potentially carry such a motif. The division between these two numbers yielded the probability of occurrence for each motif. We then subtracted the independent first-order statistics ${p}_{\alpha }^{2}$ in the case of divergent motifs (*α* → (*X*, *X*)), *p*_*α*_*p*_*β*_ for convergent motifs ((*α*, *β*) → *X*), *p*_*αβ*_*p*_*βα*_ for reciprocal motifs (*α* ↔ *β*) and *p*_*β*_*p*_*βα*_ for chain motifs (*α* → *β* → *X*) and divided by the respective *σ* values to obtain correlation coefficients ${\tau }_{\mathrm{rec},\alpha \beta },{\tau }_{\mathrm{chn},\alpha \beta },{\tau }_{\mathrm{div},\alpha \alpha }\,\mathrm{and}\,{\tau }_{\mathrm{con},\alpha \beta }$ for the individual populations (Fig. 5b).

For Fig. 5c, to compare the empirical distribution to a null hypothesis, we generated ensembles of eight neurons (as many as simultaneously probed in the dataset) and randomly defined each neuron as either excitatory or inhibitory. We randomly generated inhibitory or excitatory synapses, with probabilities matching the probability of having excitatory or inhibitory synapses in the dataset. On this ‘random’ dataset, we then performed the same analysis as on the empirical dataset and subsampled all statistics 1,000 times. Each of these subsamples contained 80% of the total statistics, that is, 80% of all sessions chosen randomly. Finally we computed *R*_motifs_ for each of these (Fig. 5c). Analyses on human data were performed with the same procedures (Fig. 5d).

### Statistics and reproducibility

No statistical method was used to predetermine sample size. Sample sizes were determined by the availability of datasets passing standard preprocessing and analysis quality control criteria. Data were not excluded based on the final analysis outcomes; however, analyses excluded low-quality calcium regions of interest, movement-defined time bins where applicable, sessions lacking required HMM outputs, low-confidence or very short state periods, unsuccessful covariance fits and synaptic pairs or cell groups that did not meet probing or annotation criteria. Experiments were not randomized. Random sampling was used only for bootstrap analyses and randomized network controls. Investigators were not blinded to allocation during experiments or outcome assessment.

### Reporting summary

Further information on research design is available in the Nature Portfolio Reporting Summary linked to this article.

## Online content

Any methods, additional references, Nature Portfolio reporting summaries, source data, extended data, supplementary information, acknowledgements, peer review information; details of author contributions and competing interests; and statements of data and code availability are available at https://doi.org/10.1038/s41593-026-02395-w.

## Supplementary information

Supplementary information Supplementary Figs. 1–10, Discussion and Details on analyses and theory.

Reporting Summary

Supplementary Video Video of activity in the visual cortex.

## Data Availability

The data that support the findings of this study are publicly available in a codeocean collection (https://codeocean.technionneuroailab.com/collections/e85debba-078a-4e64-85be-8e72a175c02d) and in a Zenodo repository (https://doi.org/10.5281/zenodo.18503652)^76^.

## Code availability

The code for all numerical simulations and data analyses are publicly available in a codeocean collection (https://codeocean.technionneuroailab.com/collections/e85debba-078a-4e64-85be-8e72a175c02d) and in a Zenodo repository (https://doi.org/10.5281/zenodo.18503652)^76^.
