# Supplementary material for: Strong and localized recurrence controls the dimensionality of neural activity across brain areas
Source: Nat Neurosci. 2026 Aug 19;29(10):2578–87. doi: 10.1038/s41593-026-02395-w (PMC13633124; doi:10.1038/s41593-026-02395-w)
Supplement: Supplementary file 1 [file 41593_2026_2395_MOESM1_ESM.pdf]

nature neuroscience

Supplementary information https://doi.org/10.1038/s41593-026-02395-w

Strong and localized recurrence controls the dimensionality of neural activity across brain areas

In the format provided by the authors and unedited

Strong and localized recurrence controls the dimensionality of neural activity across brain areas

Supplementary Material

David Dahmen, Stefano Recanatesi, Xiaoxuan Jia, Gabriel K. Ocker, Nilufar Lahiji, Simon Musall, Luke Campagnola, Stephanie Seeman, Tim Jarsky, Moritz Helias, Eric Shea-Brown

Contents

1 Bias correction in the statistics of covariances **2**

2 Extraction and analysis of intrinsic covariances **6**
- 2.1 Relation between intrinsic covariances and network recurrency . . . 6
- 2.2 Effect of other covariance statistics on dimensionality . . . 6
- 2.3 Shared covariances from correlated external inputs . . . 7
- 2.4 Extraction of intrinsic covariances from driven network dynamics using Latent Factor Analysis . . . 9

3 Fitting linear network models to experimental data **11**

4 Field theory of spectral radius **14**
- 4.1 Derivation of spectral radius in homogeneous networks with second-order motifs . . . 14
  - 4.1.1 Moment generating functional and disorder average . . . 14
  - 4.1.2 Second order motifs . . . 14
  - 4.1.3 Outlier eigenvalues of connectivity and relation to the propagator . . . 16
    - Outlier eigenvalues of $\boldsymbol{W}$ and dimensionality . . . 17
    - Excitation-dominated networks . . . 17
    - Inhibition-dominated networks . . . 17
  - 4.1.4 Spectral radius and correlation functions . . . 18
- 4.2 Generalization to networks with multiple populations . . . 18
- 4.3 From covariances of connections to motif abundances . . . 20

5 Constraints on motif abundances and algorithms for network construction **21**

6 Reanalysis of synaptic physiology data from Song et al. **24**

7 Layer- and cell-type resolved analysis of synaptic physiology dataset **24**
- 7.1 Connection probabilities and motif abundances . . . 24
- 7.2 Analysis of σ . . . 25
- 7.3 Analysis of R_{motifs} . . . 25

8 Theory of σ in multi-population networks with statistics depending on pre- and post-synaptic population identities **27**
- 8.1 Relative recurrency . . . 27

1 Bias correction in the statistics of covariances

For experimental data, covariances are estimated based on a limited number of trials. In consequence, the estimators for the dispersion of variances and covariances may be biased towards larger values. To find analytical correction formulas for this finite data bias we assume a Gaussian probability distribution $p(n^1, ..., n^{N_T})$, $n^k \in \mathbb{N}_0^N$, of activities n_i^k of neuron i in trial $k \in \{1, ..., N_T\}$. We further assume that there are no correlations between different trials, so that the probability distribution factorizes over trials and the moment generating function is

$$\phi(l^1, ..., l^{N_T}) = \prod_{k=1}^{N_T} \exp\left(m^{\mathrm{T}} l^k + \frac{1}{2} l^{k,\mathrm{T}} c l^k\right), \tag{S1}$$

where m is the vector of mean activities, c is the covariance matrix with the following meta-statistics: mean variance $\overline{a}$, variance of variances δa^2, mean covariance $\overline{c}$ and variance of covariances δc^2 across neurons. Since we are only interested in the statistics of covariances, we can, without loss of generality, set all mean activities to zero

$$\phi(l^1, ..., l^{N_T}) \to \prod_i \exp\left(c_{ii} \frac{1}{2} \sum_{k=1}^{N_T} l_i^k l_i^k\right) \prod_{i<j} \exp\left(c_{ij} \sum_{k=1}^{N_T} l_i^k l_j^k\right) \tag{S2}$$

The meta-statistics are assumed to be identical across trials. Furthermore, we assume the meta-statistics to be the same for all realizations of distributions of covariances across neurons. In the following, $\langle\rangle$ denotes the average over these realizations obtained from the averaged moment generating function

$$\begin{aligned}
\left\langle \phi(l^1, ..., l^{N_T}) \right\rangle &= e^{\bar{\Phi}(l^1, ..., l^{N_T})} \\
\bar{\Phi}(l^1, ..., l^{N_T}) &= \frac{1}{2} \overline{a} \sum_i \sum_{k=1}^{N_T} l_i^k l_i^k + \frac{1}{8} \delta a^2 \sum_i \sum_{k,l=1}^{N_T} l_i^k l_i^k l_i^l l_i^l \\
&+ \frac{1}{2} \overline{c} \sum_{i \neq j} \sum_{k=1}^{N_T} l_i^k l_j^k + \frac{1}{4} \delta c^2 \sum_{i \neq j} \sum_{k,l=1}^{N_T} l_i^k l_j^k l_i^l l_j^l .
\end{aligned}$$

Therefore we obtain second and fourth order cumulants of activities

$$\begin{aligned}
\langle n_a^m n_b^n \rangle = \langle\langle n_a^m n_b^n \rangle\rangle &= \left. \frac{\partial^2 \bar{\Phi}}{\partial l_a^m \partial l_b^n} \right|_{l=0} \\
&= \delta_{mn} \left(\delta_{ab} \overline{a} + (1 - \delta_{ab}) \overline{c} \right) \ , \\
\langle\langle n_a^m n_b^n n_c^o n_d^p \rangle\rangle &= \left. \frac{\partial^4 \bar{\Phi}}{\partial l_a^m \partial l_b^n \partial l_c^o \partial l_d^p} \right|_{l=0} \\
&= \delta a^2 \delta_{ab} \delta_{ac} \delta_{ad} \left(\delta_{mn} \delta_{op} + \delta_{mo} \delta_{np} + \delta_{mp} \delta_{no} \right) \\
&+ \delta c^2 \delta_{ac} \delta_{bd} \left(1 - \delta_{ab} \right) \left(\delta_{mn} \delta_{op} + \delta_{mp} \delta_{no} \right) \\
&+ \delta c^2 \delta_{ab} \delta_{cd} \left(1 - \delta_{ac} \right) \left(\delta_{mo} \delta_{np} + \delta_{mp} \delta_{no} \right) \\
&+ \delta c^2 \delta_{ad} \delta_{bc} \left(1 - \delta_{ab} \right) \left(\delta_{mn} \delta_{op} + \delta_{mo} \delta_{np} \right) .
\end{aligned}$$

Let $\hat{}$ denote the empirical estimates of mean activities and covariances from N recorded neurons in N_T trials of the experiment. Unbiased estimators for sample means and covariances are (using Bessel's correction)

$$\begin{aligned}
\hat{m}_i &= \frac{1}{N_T} \sum_{k=1}^{N_T} n_i^k \ , \\
\hat{c}_{ij} &= \frac{1}{N_T - 1} \sum_{k=1}^{N_T} (n_i^k - \hat{m}_i)(n_j^k - \hat{m}_j) \, .
\end{aligned}$$

The latter can be expressed purely in terms of activities

$$\hat{c}_{ij} = \sum_{k,l=1}^{N_T} \left(\frac{\delta_{kl}}{N_T} - \frac{1-\delta_{kl}}{N_T(N_T-1)} \right) n_i^k n_j^l .$$

These estimators are unbiased because

$$\begin{aligned}
\langle \hat{m}_i \rangle &= \frac{1}{N_T} \sum_{k=1}^{N_T} \underbrace{\langle n_i^k \rangle}_{\bar{m}} = \bar{m} = 0 \,, \\
\langle \hat{c}_{ij} \rangle &= \sum_{k,l=1}^{N_T} \left(\frac{\delta_{kl}}{N_T} - \frac{1-\delta_{kl}}{N_T(N_T-1)} \right) \langle n_i^k n_j^l \rangle \\
&= \sum_{k,l=1}^{N_T} \left(\frac{\delta_{kl}}{N_T} - \frac{1-\delta_{kl}}{N_T(N_T-1)} \right) \delta_{kl} \left(\delta_{ij} \overline{a} + (1-\delta_{ij}) \overline{c} \right) \\
&= \delta_{ij} \overline{a} + (1-\delta_{ij}) \overline{c} \ .
\end{aligned}$$

The mean covariance defined as

$$\hat{\overline{a}} = \frac{1}{N} \sum_{i=1}^{N} \hat{c}_{ii} \,, \tag{S3}$$

$$\hat{\overline{c}} = \frac{1}{N(N-1)} \sum_{i \neq j}^{N} \hat{c}_{ij} \tag{S4}$$

is therefore also unbiased

$$\langle \hat{\overline{a}} \rangle = \frac{1}{N} \sum_{i=1}^{N} \langle \hat{c}_{ii} \rangle = \overline{a} \,, \tag{S5}$$

$$\langle \hat{\overline{c}} \rangle = \frac{1}{N(N-1)} \sum_{i \neq j}^{N} \langle \hat{c}_{ij} \rangle = \overline{c} \,. \tag{S6}$$

The variance of variances and covariances needs to be properly defined using Bessel's correction

$$\begin{aligned}
\delta \hat{a}^2 &= \frac{1}{N-1} \sum_{a=1}^{N} \left(\hat{c}_{aa} - \hat{\overline{a}} \right)^2 \\
&= \sum_{a,b}^{N} \left(\frac{\delta_{ab}}{N} - \frac{1-\delta_{ab}}{N(N-1)} \right) \hat{c}_{aa} \hat{c}_{bb} \,, \\
\delta \hat{c}^2 &= \frac{1}{N(N-1)-2} \sum_{a \neq b}^{N} \left(\hat{c}_{ab} - \hat{\overline{c}} \right)^2 \\
&= \frac{1}{N(N-1)} \sum_{a,b \neq}^{N} \hat{c}_{ab} \hat{c}_{ab} \\
&\quad - 4 \frac{1}{N(N-1)-2} \frac{1}{N(N-1)} \sum_{a,b,d \neq}^{N} \hat{c}_{ab} \hat{c}_{ad} \\
&\quad - \frac{1}{N(N-1)-2} \frac{1}{N(N-1)} \sum_{a,b,c,d \neq}^{N} \hat{c}_{ab} \hat{c}_{cd} \,.
\end{aligned}$$

Note that Bessel's correction amounts to a normalization $\frac{1}{N-1}$ for the variances (subtract one degree of freedom as usual) and $\frac{1}{N(N-1)-2}$ for covariances (subtract two degrees of freedom due to symmetry $\hat{c}_{ab} = \hat{c}_{ba}$). These corrections are the right ones as can be seen by calculating the estimated variances of variances

$$\begin{aligned}\left\langle \hat{\delta a^2} \right\rangle &= \sum_{a,b}^{N} \left(\frac{\delta_{ab}}{N} - \frac{1-\delta_{ab}}{N(N-1)} \right) \langle \hat{c}_{aa}\hat{c}_{bb} \rangle \\ &= \delta a^2 + \frac{2\left(\delta a^2 - \delta c^2\right)}{N_T - 1} + \frac{2\left(\overline{a}^2 - \overline{c}^2\right)}{N_T - 1}\end{aligned}$$

and covariances

$$\begin{aligned}\left\langle \hat{\delta c^2} \right\rangle &= \frac{1}{N(N-1)} \sum_{a,b\neq}^{N} \langle \hat{c}_{ab}\hat{c}_{ab} \rangle \\ &- 4\frac{1}{N(N-1)-2}\frac{1}{N(N-1)} \sum_{a,b,d\neq}^{N} \langle \hat{c}_{ab}\hat{c}_{ad} \rangle \\ &- \frac{1}{N(N-1)-2}\frac{1}{N(N-1)} \sum_{a,b,c,d\neq}^{N} \langle \hat{c}_{ab}\hat{c}_{cd} \rangle \\ &= \delta c^2 + \frac{\delta c^2 + \overline{a}^2 - \overline{c}^2}{N_T - 1} + \frac{4}{N+1}\frac{\overline{c}^2 - \overline{ac}}{N_T - 1},\end{aligned}$$

where the true variances δa^2 and δc^2 on the right hand sides of the equations are not receiving any correction due to the number of recorded neurons N. To obtain the above expressions, we used

$$\begin{aligned}\langle \hat{c}_{ab}\hat{c}_{cd} \rangle = &\sum_{m,n,o,p=1}^{N_T} \left(\frac{\delta_{mn}}{N_T} - \frac{1-\delta_{mn}}{N_T(N_T-1)} \right) \left(\frac{\delta_{op}}{N_T} - \frac{1-\delta_{op}}{N_T(N_T-1)} \right) \langle n_a^m n_b^n n_c^o n_d^p \rangle \\ &= \delta a^2 \delta_{ab}\delta_{ac}\delta_{ad} \left(1 + \frac{2}{N_T - 1} \right) \\ &+ \delta c^2 \delta_{ac}\delta_{bd} \left(1 - \delta_{ab}\right) \left(1 + \frac{1}{N_T - 1} \right) \\ &+ \delta c^2 \delta_{ab}\delta_{cd} \left(1 - \delta_{ac}\right) \frac{2}{N_T - 1} \\ &+ \delta c^2 \delta_{ad}\delta_{bc} \left(1 - \delta_{ab}\right) \left(1 + \frac{1}{N_T - 1} \right) \\ &+ \left(\delta_{ab}\overline{a} + (1-\delta_{ab})\overline{c}\right) \left(\delta_{cd}\overline{a} + (1-\delta_{cd})\overline{c}\right) \\ &+ \frac{1}{N_T - 1} \left(\delta_{ad}\overline{a} + (1-\delta_{ad})\overline{c}\right) \left(\delta_{bc}\overline{a} + (1-\delta_{bc})\overline{c}\right) \\ &+ \frac{1}{N_T - 1} \left(\delta_{ac}\overline{a} + (1-\delta_{ac})\overline{c}\right) \left(\delta_{bd}\overline{a} + (1-\delta_{bd})\overline{c}\right)\end{aligned}$$

and

$$\left\langle n_i^m n_j^n n_k^o n_l^p \right\rangle = \left\langle\left\langle n_i^m n_j^n n_k^o n_l^p \right\rangle\right\rangle + \left\langle n_i^m n_j^n \right\rangle \left\langle n_k^o n_l^p \right\rangle + \left\langle n_i^m n_l^p \right\rangle \left\langle n_j^n n_k^o \right\rangle + \left\langle n_i^m n_k^o \right\rangle \left\langle n_j^n n_l^p \right\rangle . \tag{S7}$$

In summary, these results provide a procedure to infer the true statistics of covariances from estimators based on N recorded neurons in N_T trials:

$$
\begin{aligned}
\overline{a} &= \hat{\overline{a}}\,,\\
\overline{c} &= \hat{\overline{c}}\,,\\
\delta a^2 &= \frac{N_T-1}{N_T+1}\delta\hat{a}^2 - \frac{2\left(\hat{\overline{a}}^2-\hat{\overline{c}}^2\right)}{N_T+1} + \frac{2\delta c^2}{N_T+1}\,,\\
\delta c^2 &= \frac{N_T-1}{N_T}\delta\hat{c}^2 - \frac{\hat{\overline{a}}^2-\hat{\overline{c}}^2}{N_T} - \frac{4}{N+1}\frac{\hat{\overline{c}}^2-\hat{\overline{a}}\hat{\overline{c}}}{N_T}\,,
\end{aligned}
$$

with

$$
\begin{aligned}
\hat{\overline{a}} &= \frac{1}{N}\sum_{i=1}^{N}\hat{c}_{ii}\,,\\
\hat{\overline{c}} &= \frac{1}{N(N-1)}\sum_{i\neq j}^{N}\hat{c}_{ij}\,,\\
\delta\hat{a}^2 &= \frac{1}{N-1}\sum_{a=1}^{N}\left(\hat{c}_{aa}-\hat{\overline{a}}\right)^2\,,\\
\delta\hat{c}^2 &= \frac{1}{N(N-1)-2}\sum_{a\neq b}^{N}\left(\hat{c}_{ab}-\hat{\overline{c}}\right)^2\,.
\end{aligned}
$$

2 Extraction and analysis of intrinsic covariances

The participation ratio (Eq. 1) formally depends on all first and second moments of variances and covariances. Yet, for large recordings, due to the large prefactor $N-1$ in the denominator, mainly cross-neuronal coordination in form of the rescaled mean $m=\frac{\bar{c}}{\bar{a}}$ and standard deviation $s=\frac{\delta c}{\bar{a}}$ of covariances determine the dimensionality. For spontaneous activity in mouse visual cortex we found that s is much larger than m (Fig. 1i), so that in good approximation the intrinsic dimensionality is determined by the variability of intrinsic covariances across neuron pairs:

$$\frac{D_{\mathrm{PR}}}{N}=\frac{1}{1+Ns^2}\,. \tag{S8}$$

The analyses in the main text are based on the assumption that the statistics of covariances, measured within individual HMM states, are predominantly shaped by intrinsic network mechanisms and recurrent connectivity. In the following, we discuss how, in this scenario, s is related to the recurrency R of the circuit. We furthermore assess the impact of the other covariance statistics on dimensionality, and how these statistics depend on network features. Finally, we introduce a control analysis, where we assume that all low-rank network activity is not generated within the circuit but induced by correlated external input. In this scenario we design a workflow which removes such components and we verify the robustness of our results against this potential influence of external inputs.

2.1 Relation between intrinsic covariances and network recurrency

Intrinsic correlations, that are those correlations which are generated via local recurrent reverberations of fluctuations in networks, have been explained using linear response theory [1, 2, 3, 4, 5]. Linearization around some stationary state yields an effective connectivity that couples neuronal fluctuations around their mean activities [2, 6, 7]. Such fluctuations can be formally described with a "linear rate model," a coupled set of Ornstein-Uhlenbeck processes [8]

$$\tau_i\partial_t x_i(t)=-x_i(t)+\sum_{j=1}^{N}W_{ij}x_j(t)+\xi_i(t)\,, \tag{S9}$$

where the centered Gaussian white noise $\xi_i(t)$ reflects intrinsic stochasticity of neuronal processes. Its variance $\langle\xi_i(t)\xi_j(s)\rangle=\delta_{ij}\delta(t-s)D$ derives from the mean activity of neurons in the stationary state that is used as a working point for linearization [9, 7].

Reference [10] used beyond-mean field techniques to study the statistics of correlations in such systems and found that, in homogeneous networks, the width s of the covariance distribution is determined by the network recurrency R, that is the spectral radius of the effective connectivity

$$Ns^2=\frac{1}{\left(1-R^2\right)^2}-1. \tag{S10}$$

Thus, when measuring dimensionality from all $N=N^*$ neurons in the cicuit, inserting (S10) into (S8) yields a relation between the dimensionality of the full network dynamics and the recurrency of the network, via the relationship

$$\frac{D_{\mathrm{PR}}}{N}=(1-R^2)^2. \tag{S11}$$

The robustness of equation (S10) with respect to other network topologies shown in [10] implies a robustness of equation (S11) (cf. Fig. 3b of the main text).

2.2 Effect of other covariance statistics on dimensionality

Apart from the contribution of s to the dimensionality, one can employ the generalized results on the statistics of variances and covariances derived in [11] to refine how the dimensionality depends on

network parameters. More specifically, for a network with mean connectivity $\langle W_{ij} \rangle = \mu$ and variance $\langle W_{ij}^2 \rangle - \langle W_{ij} \rangle^2 = \sigma^2/N$ as well as intrinsic stochasticity of mean $\langle D_{ii} \rangle = \bar{D}$ and variance $\langle D_{ii}^2 \rangle - \langle D_{ii} \rangle^2 = \overline{\delta D^2}$ one obtains

$$\bar{a} = \left(1 + 2M + NM^2\right) \frac{\bar{D}}{1 - R^2} \tag{S12}$$

$$\bar{c} = \left(2M + NM^2\right) \frac{\bar{D}}{1 - R^2} \tag{S13}$$

and

$$\overline{\delta c^2} = \left(2M^2\right) \overline{\delta D^2} + \bar{R}\frac{1}{N}(1 + 2M)^2\overline{\delta D^2} + \bar{R}\left\{\left(2(1 + 2M)M^2 + NM^4\right)\overline{\delta D^2}\right\} + \bar{R}\frac{1}{N}\left(\bar{a}^2 + (N - 1)\bar{c}^2\right) \tag{S14}$$

$$\overline{\delta a^2} = (1 + 4M)\overline{\delta D^2} + 2\overline{\delta c^2} \tag{S15}$$

with

$$M = \frac{\mu}{1 - N\mu}$$
$$R = (1 + 2M + M^2)\sigma^2$$
$$\bar{R} = 2\frac{R^2}{1 - R^2} + \left(\frac{R^2}{1 - R^2}\right)^2$$

Note that these equations reduce to the results in [10] for $M \to 0$ and $\overline{\delta D^2} = 0$. The above expressions can be inserted in Eq. 1 to obtain a more detailed dependence of dimensionality on the network parameters. These equations are used in Fig. 2 and Sec. 3 to quantitatively fit network models to experimental data and infer the recurrency (Fig. S4).

2.3 Shared covariances from correlated external inputs

We next detail the control experiments where we assumed that correlated external inputs affect the measured covariance statistics in visual cortex spontaneous activity.

Under a linear assumption the covariance matrix of neural activity splits into two contributions: a shared and an intrinsic component (cf. Suppl. Mat. Sec. 2.4). In order to estimate these two components we developed a three stage procedure that could be performed by utilizing different algorithms at its core. Here we use Latent Factor Analysis (LFA). In the following we will explain this procedure with LFA but it would work equivalently with PCA or other algorithms. The first stage bins the spikes of neurons into spike counts within non-overlapping windows. We used 100ms bins, Fig. S1a. Then we performed LFA multiple times with an increasing number of hidden factors and computed the log-likelihood as a function of factors with a 5-fold cross-validation technique, Fig. S1b. We selected the number of factors by choosing the corresponding point in the log-likelihood curve where the cross-validated log-likelihood didn't increase more than 5% for the first time. This yielded a robust estimation of where the plateau or peak in the curve is found, Fig. S1b. The second stage estimated the activity of the shared neural activity and intrinsic neural activity by running LFA with the selected number of components, Fig. S1c. The computed shared and intrinsic covariance (Fig. S1d) yielded a first estimate of the standard deviation of intrinsic covariances δc, Fig. S1e. In the third stage we removed the bias on such estimates by subsampling the intrinsic neural activity (Fig. S1f) and computing δc as a function of the number of samples used N_T (Fig. S1g). We then fit the dependence (cf. Eq. 9 in the Methods) $\hat{\delta c}^2 = \delta c^2 + \frac{\text{const.}}{N_T}$ to extract the true value of δc from the estimates $\hat{\delta c}$. All analyses were run through custom scripts based on the scikit learn library.

Fig. S2 compares the resulting statistics of covariances and dimensionality of activity in mouse visual cortex with and without removal of low-rank components and shows that our results on low

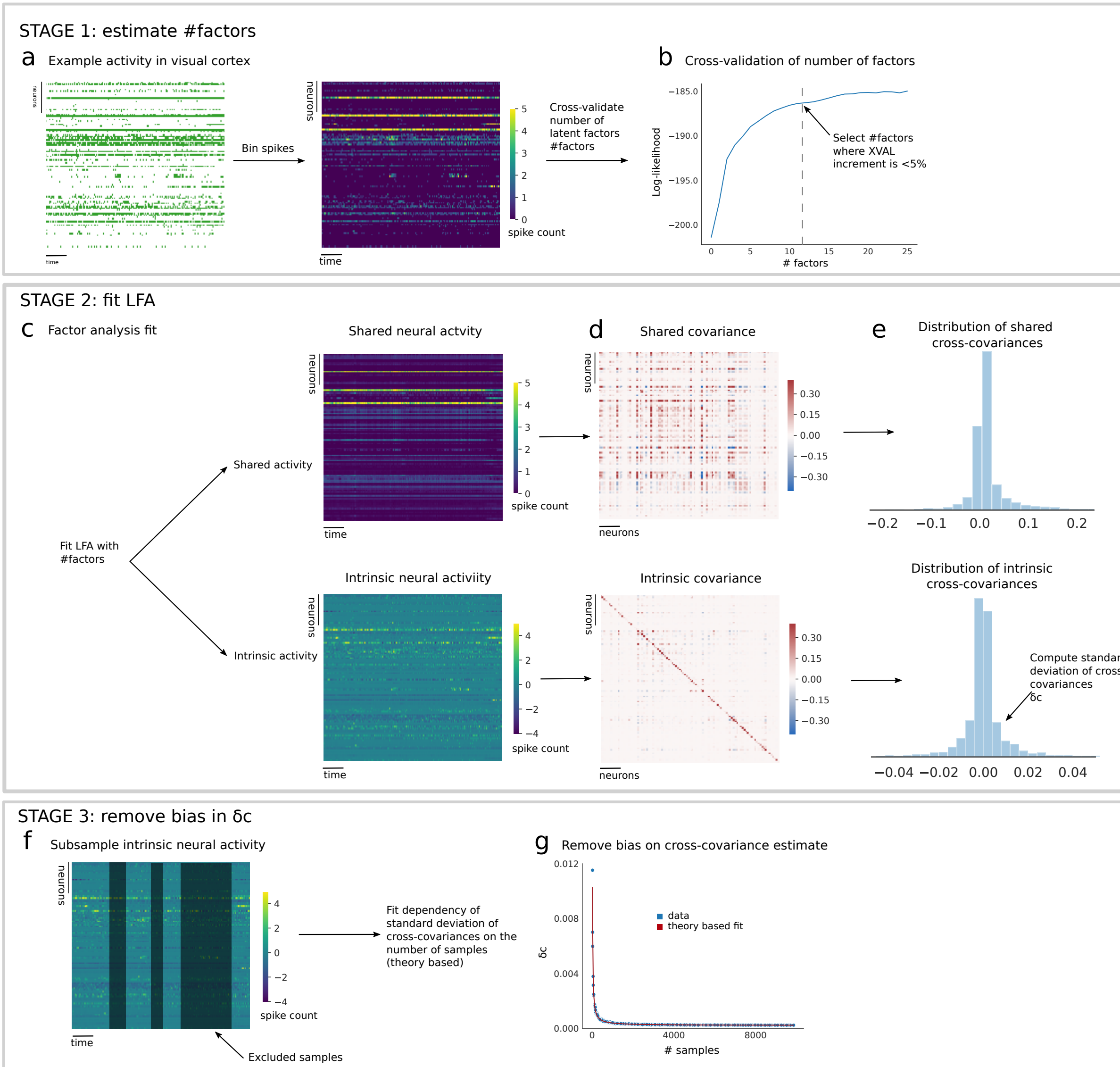

Figure S1: **Method for removing shared low-rank activity components to infer intrinsic activity statistics.** This figure illustrates the cross-validated Latent Factor Analysis (LFA) method applied to the activity within various neural states, in order to extract low-rank components that could have originated from external inputs. This analysis validates our results on strongly constrained dimensionality of the experimentally recorded neural activity against the potential influence of external inputs. In Fig. S2, the resulting statistics of covariances and dimensionality of activity are compared with and without removal of low-rank components. In addition, the figure illustrates how we eliminate the bias in the covariance statistics due to a finite number of samples (time bins) and obtain an unbiased estimate of the covariance standard deviation (for details, see Sec. 1), as is needed to obtain an unbiased estimate of the dimensionality.
The full procedure works as follows: a) Bin spikes of each recorded neuron in each experimental session into 100ms windows to obtain spike-count vectors. b) Perform 5 fold cross-validated latent factor analyses (LFA) with increasing number of factors (from 1 to 25). Determine log-likelihood as a function of the number of factors. Select number of factors (#factors) as the minimal one that yields a relative increment of the cross-validation curve lower than 5%. c) Perform LFA with #factors to split total activity into shared and intrinsic activity components. d) Compute covariance of shared and intrinsic components. e) Compute distribution of covariances for shared and intrinsic components. f) Subsample intrinsic neural activity and compute standard deviation of covariances δc as a function of samples (#bins) used. g) Fit dependence of δc on the number of bins to estimate and remove bias induced by the finite duration of the recording (see Methods). This procedure yielded an unbiased estimate of the statistics of intrinsic variances and covariances.

dimensionality relative to network sizes are valid even if we assume all low-rank activity to be attributed to external inputs.

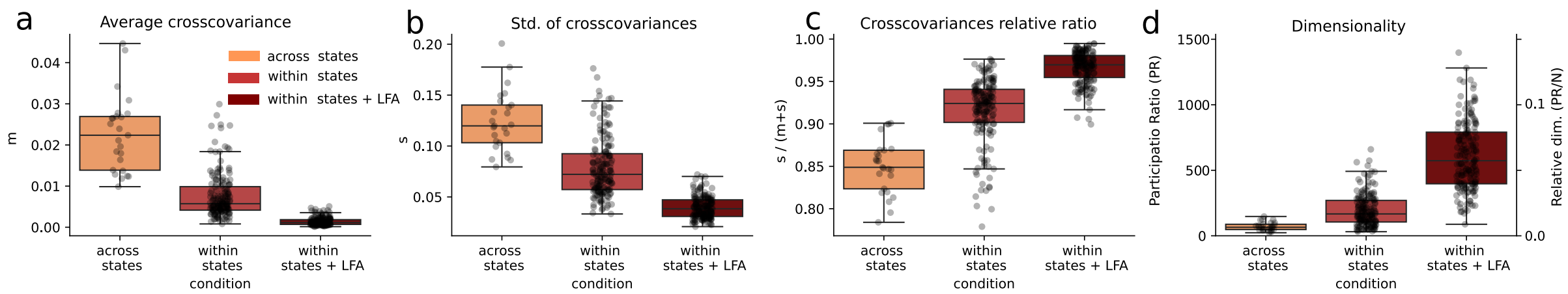

Figure S2: **Covariance and dimensionality analysis before and after subtraction of low-rank activity components.** This figure shows the results of the cross-validated Latent Factor Analysis (LFA) on the visual cortex data. LFA removes low-rank activity components. The remaining activity is less correlated and therefore higher dimensional. As we show in Fig. S3, LFA not only removes activity components that potentially stem from correlated external input, but also to a certain extent intrinsically generated correlations. The dimensionality values after applying LFA should therefore be considered as upper bounds (see Fig. S3). a) Normalized covariances $m = \frac{\bar{c}}{\bar{a}}$ averaged across neurons and across the entire experimental session (orange), averaged within each neural state (red) and after performing LFA for each state (bordeaux). b) Same as a but for the normalized standard deviation of covariances $s = \frac{\delta c}{\bar{a}}$. c) Same as a for the relative ratio ($s/(s+m)$). d) Same as a for the dimensionality measured by the participation ratio (PR), Eq. 1. Left scale (y-axis) extrapolated dimensionality to 10^4 neurons, right scale (y-axis) in percentage normalized to the number of neurons. Boxes show median and IQR, whiskers show 1.5 IQR. Overlaid points show individual observations.

2.4 Extraction of intrinsic covariances from driven network dynamics using Latent Factor Analysis

In this section we apply the latent factor analysis (LFA) performed on the experimental data (Fig. S1) to model data, from an input-driven network model with known ground truth intrinsic activity and external drive. To construct the model data we make use of the linear rate model Eq. S9 introduced above.

For networks that receive correlated external inputs, the mean of those inputs changes the firing rate as well as the gain of neurons, and thereby the effective connectivity W [11]. Temporal fluctuations of external inputs directly drive local correlations. This latter effect can be modeled in the framework above as an additional independent, centered and correlated external input $\eta(t)$, which we here assume to have a covariance $\langle \eta(t)\eta(s)^{\mathrm{T}} \rangle = \delta(t-s)D_{\mathrm{external}}$.

We are interested in covariances of spike counts computed for large time bins. These covariances are equivalent to time-lag integrated covariances or zero-frequency cross-spectra [10]. For the network dynamics Eq. S9 including the additional external drive η, we obtain these zero-frequency cross-spectra by studying the zero-frequency Fourier mode X of the dynamics

$$0 = -X + WX + \tilde{\xi} + \tilde{\eta} \tag{S16}$$

where $\tilde{\xi}$ and $\tilde{\eta}$ are the zero-frequency components of the inputs $\xi(t)$ and $\eta(t)$, respectively. $\tilde{\xi}$ and $\tilde{\eta}$ follow Gaussian statistics with zero mean and covariance $D_{\mathrm{intrinsic}}$ and D_{external}, respectively. Eq. (S16) can be solved for X, such that

$$X = (1-W)^{-1}(\tilde{\xi} + \tilde{\eta}). \tag{S17}$$

The zero-frequency mode X therefore follows Gaussian statistics

$$X \sim \mathcal{N}(0, C) \tag{S18}$$

with

$$C = C_{\mathrm{intrinsic}} + C_{\mathrm{external}} \tag{S19}$$

$$C_{\mathrm{intrinsic}} = (1-W)^{-1}D_{\mathrm{intrinsic}}(1-W^{\mathrm{T}})^{-1} \tag{S20}$$

$$C_{\mathrm{external}} = (1-W)^{-1}D_{\mathrm{external}}(1-W^{\mathrm{T}})^{-1}. \tag{S21}$$

The theory developed in this study linking dimensionality and network recurrency, see Sec. S4.1-4.2, strictly holds for intrinsic network activity, i.e. $D_{\text{external}} = 0$. In the experiments we analyzed, however, local recorded networks are potentially driven by external inputs as well. Such external inputs may affect local covariances and the dimensionality. This effect could even persist after removing strong activity transients by restricting the analysis to individual HMM states. In our control experiments we first extracted the intrinsic covariances $C_{\text{intrinsic}}$ from the measured covariances C by removing the externally induced components C_{external}.

The extraction of $C_{\text{intrinsic}}$ is based on the following conservative assumption: all low-rank activity inferred by Latent Factor Analysis [12] is attributed to external inputs. Latent Factor Analysis uses an expectation maximization procedure to infer low-dimensional latent factors $\tilde{X}$ in the activity X such that the covariance

$$C_{LFA} = C_{\text{shared}} + R$$

can be split into a shared low-rank component $C_{\text{shared}} = \langle X_{\text{shared}} X_{\text{shared}}^{\mathrm{T}} \rangle$ and a diagonal remainder R. Here, X_{shared} denotes the projection of the low-dimensional latent factors into the high-dimensional space of neurons using the loading matrix L: $X_{\text{shared}} = L\tilde{X}$. In case of an ideal fit, $C = C_{LFA}$ and latent factors have zero mean and unit covariance such that the shared covariance reduces to $C_{\text{shared}} = LL^T$. However, since the above splitting procedure cannot be done exactly for every covariance matrix C, the above operation is in general an approximation. Therefore we introduce the covariance ΔC as the mismatch between C and C_{LFA}, and write

$$\begin{aligned} C &= C_{\text{shared}} + R + \Delta C \\ &=: C_{\text{shared}} + \hat{C}_{\text{intrinsic}}. \end{aligned}$$

Assuming that the external component C_{external} is the only low-rank component of C, LFA yields $C_{\text{shared}} = C_{\text{external}}$ such that $\hat{C}_{\text{intrinsic}} = C_{\text{intrinsic}}$. In this case, we can extract an estimate of $C_{\text{intrinsic}}$ as

$$\hat{C}_{\text{intrinsic}} = C - C_{\text{shared}} \,, \tag{S22}$$

where $C = \langle XX^{\mathrm{T}} \rangle$ and $C_{\text{shared}} = \langle X_{\text{shared}} X_{\text{shared}}^{\mathrm{T}} \rangle$.

Fig. S3 shows a systematic analysis of the above procedure for the case of strong ($\langle \eta_i^2 \rangle = \langle \xi_i^2 \rangle$), weak ($\langle \eta_i^2 \rangle = 0.1 \langle \xi_i^2 \rangle$) and vanishingly small ($\langle \eta_i^2 \rangle = 0.01 \langle \xi_i^2 \rangle$) external input. For all these input scenarios, the LFA procedure yields conservative estimates for the intrinsic covariances, as well as the recurrent coupling and dimensionality: Strong external inputs significantly broaden the distribution of covariances (Fig. S3a) and change the spectrum of covariance eigenvalues for low PC dimensions (Fig. S3b). As a result, the dimensionality is reduced (Fig. S3c, Fig. S3d). Both effects can be removed by extracting $\hat{C}_{\text{intrinsic}}$ via a latent factor analysis. This works well for low and intermediate spectral radii, as can be seen from the correct inference of the recurrency and dimensionality in these regimes (Fig. S3e, Fig. S3f). For large spectral radii, the dimensionality of $C_{\text{intrinsic}}$ is low, so that C_{shared} of LFA not only extracts C_{external} but also components from $C_{\text{intrinsic}}$. The vanishingly small input case thereby serves as a control, because almost all inferred low-rank activity is not due to external input but rather due to intrinsic activity. The results for this control case and the other input cases show that the LFA procedure with parameters chosen as for the experimental data underestimates recurrency and overestimates intrinsic dimensionality. Therefore LFA yields conservative results in terms of how strongly connectivity constrains dimensionality.

We deliberately chose a rather simple model setting for the parameter scan in the previous results. In particular, low-rank external inputs were projected into the network with random loadings drawn from a Gaussian distribution. One consequence of this simple choice of external input is for example the gap in the spectrum of eigenvalues in Fig. S3b that is typically not present in experimental data. In the next section, we show that linear models can be fit to better reproduce the experimentally observed covariance features.

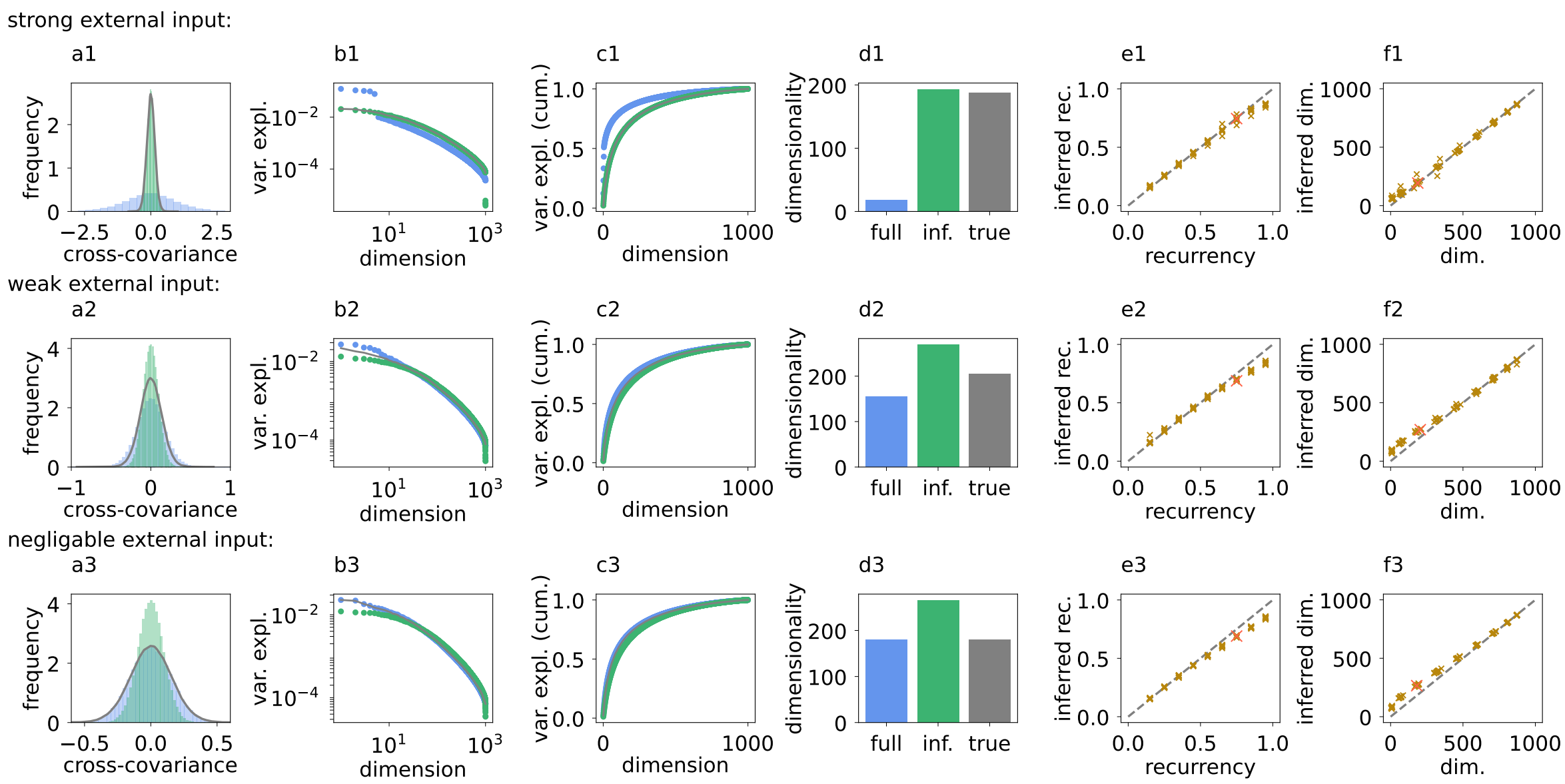

Figure S3: **Latent Factor Analysis applied to simulation data.** This figure applies the cross-validated LFA procedure to model data generated by simulating a network model. This allows us to validate whether the cross-validated LFA technique recovers the known ground truth. The figure shows that LFA faithfully removes externally driven low-rank components of activity, but also tends to remove strong intrinsically generated activity components that arise from local reverberation of fluctuations via strong recurrent connections. Consequently, the recurrency inferred from simulated activity is typically underestimated, resulting in an overestimation of the inferred dimensionality of intrinsic activity, especially in the strongly recurrent regime. Therefore, the LFA procedures yield a conservative estimate of how low the dimensionality of intrinsic activity is. The dimensionality values shown in Fig. S2 (within states + LFA) can therefore be interpreted as upper bounds to the true values. For more details, see Sec. 2.4. The figure shows that LFA yields an upper bound on the dimensionality of intrinsic network activity in linear rate networks. Correlated external inputs can in principle strongly alter covariances within the network, but LFA can extract intrinsic covariances over a wide range of recurrencies (spectral radii) and ranks of external input covariances. In the strongly recurrent regime, our LFA procedure tends to excessively (incorrectly) subtract strong intrinsic activity components, leading to conservative results on recurrency and intrinsic dimensionality. Top row: strong external input ($d_{\text{ext}} = d_{\text{int}}$). Middle row: weak external input ($d_{\text{ext}} = 0.1 d_{\text{int}}$). Bottom row: negligible external input ($d_{\text{ext}} = 0.01 d_{\text{int}}$). a) A low rank input can lead to broader distributions of covariances (blue) with respect to intrinsically generated covariances (gray). Applying LFA, inferred intrinsic covariances (green) recover features of ground-truth intrinsic covariances well. Parameters: recurrency $R = 0.75$, external input $\text{rank}(D_{ext}) = 5$. b) Covariance spectra corresponding to distributions in a. c) Cumulative variance explained corresponding to distributions in a. d) Dimensionality of full, inferred intrinsic and true intrinsic activity corresponding to distributions in a. e) Inferred recurrency vs ground truth recurrency for networks of different recurrency R and different ranks of external input (between 1 and 20). Red marker shows configuration shown in a-d. f) Inferred intrinsic dimensionality vs ground truth intrinsic dimensionality for networks of different recurrency R and different ranks of external input (between 1 and 20). More details: see Sec. 2.4.

3 Fitting linear network models to experimental data

Eq. S12-Eq. S15 relate the mean and variance of intrinsic variances and covariances to the five network parameters $\mu, \sigma, \bar{D}, \overline{\delta D^2}$ and N^*, which are the mean and standard deviation of connections, the mean and variance of intrinsic neuron stochasticity $D_{\text{intrinsic}}$ and the network size, respectively. For every network size N^*, we can invert these relations and find network parameters to reproduce the given means and variances of covariance in the experimental data. Although the fit is only based on the four statistics of covariances, Fig. S4 shows that the model can largely reproduce the curves of variance explained and many features of the covariance distribution. In the top row, where all activity is assumed to be generated intrinsically (no correlated external input), a few strong covariances cannot be captured by the model (Fig. S4a1); this, however, does not have a strong impact on the shape of the variance explained curves (Fig. S4b, Fig. S4c). These unpredicted strong covariances get subtracted using the

LFA procedure, which is shown in the bottom row where low-rank activity components have been removed using LFA (Fig. S4a2). In this latter case, we assumed that all low-rank activity stems from external input. The remaining activity can also be fit well by the linear model. In both scenarios (top: all activity is treated as intrinsic, bottom: only activity after LFA subtraction is treated as intrinsic), the linear network models need to operate in the strongly recurrent regime to reproduce the data (Fig. S4e).

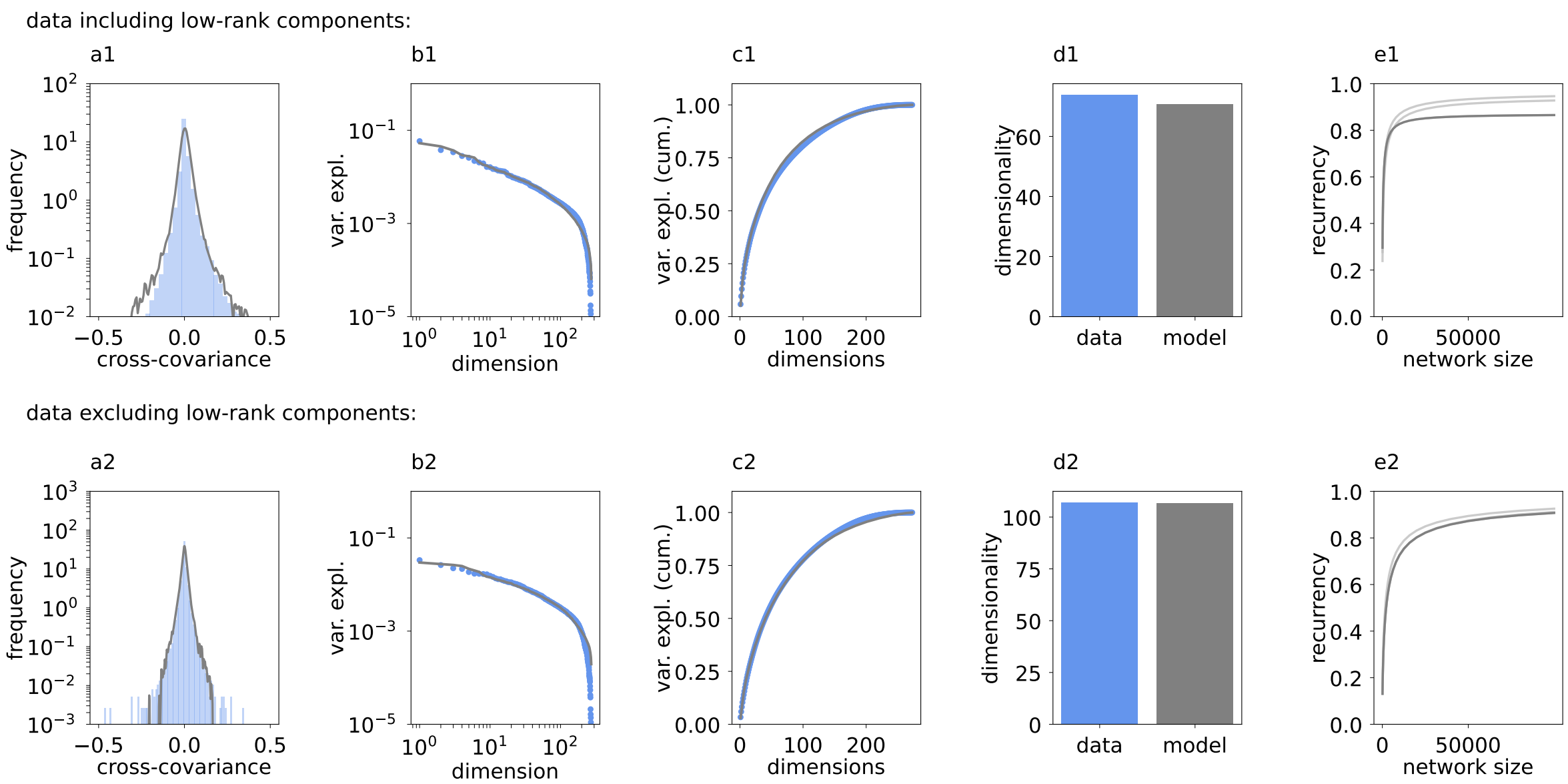

Figure S4: **Fitting of linear network model to mean and standard deviation of variances and covariances of HMM states in visual cortex.** This figure demonstrates that the network model used in our theoretical study, when fit to the experimentally measured mean and standard deviation of variances and covariances, can accurately describe the experimental activity data in terms of the covariance eigenvalue spectra and dimensionality. For networks of reasonable size, the fit model must operate in the strongly recurrent regime in order to adequately explain the data, even in the extreme case where all low-rank activity components are attributed to external inputs. Overall, the analysis indicates that a linear network model operating in the strongly recurrent regime is a minimal but adequate model for capturing the experimentally observed covariance statistics and dimensionality. For more details, see Sec. 3. The figure shows a linear rate model with Gaussian white noise input of variable strength across neurons that captures many features of experimentally observed covariances and dimensionality. Top row: fit to data of one example HMM state without removal of low-rank activity components. Bottom row: fit to data of the same HMM state after removal of low-rank activity components using LFA. Even if all removed low-rank activity components were attributed to correlated external inputs and only the activity after LFA is considered to be intrinsic, for reasonable network sizes the network still needs to operate in the strongly recurrent regime to reproduce the data. a) Distribution of experimentally observed covariances (blue) and model fit (gray) in logarithmic frequency scale. b) Variance explained of individual dimensions for data and model fit (rescaled and ranked PCA eigenvalues). c) Cumulative variance explained of data and model fit. d) Dimensionality (participation ratio) of data and model fit. e Inferred recurrency of the fit model as a function of the network size. Dark gray curve shows example HMM state shown in panels a - d. Light gray curves show other HMM states of example session. Parameters: Panels a - d show results of model fits with network size $N = 275$, which equals the number of recorded neurons in the example session. More details: see Sec. 3.

In Fig. S5 we analyze how the dimensionality of network activity changes with recurrency in the presence of external input. To do this for plausible external inputs we follow a five-step procedure: 1) We perform LFA to split the covariance of a given HMM state into $C_{\mathrm{intrinsic}}$ and C_{external}. 2) We fit network parameters $\mu, \sigma, \bar{D}, \overline{\delta D^2}$ on the mean and variances of variances and covariances $C_{\mathrm{intrinsic}}$ (cf. Fig. S4 bottom). For the fitting, we choose the network size to be equal to the number of recorded neurons in the data. 3) We construct a realization of a Gaussian connectivity matrix W and a Gaussian noise matrix $D_{\mathrm{intrinsic}}$ based on the inferred network parameters. 4) We infer the external input covariance D_{external} based on C_{external} obtained in step 1 and the connectivity matrix W obtained in step 3 via $D_{\mathrm{external}} = (1 - W)C_{\mathrm{external}}(1 - W^T)$. 5) We rescale the connectivity to modify the recurrency of the model to a desired value leaving all other network and input parameters ($\mu, D_{\mathrm{intrinsic}}, D_{\mathrm{external}}$) the same.

The resulting change in dimensionality with recurrency for fixed external input is shown in Fig. S5 as an average over 50 repetitions of the procedure (realizations of W). The external input overall reduces the dimensionality, but the general trend with which dimensionality is further reduced with stronger recurrency is similar to the case without inputs. Also similarly to the case without inputs, the relative change in dimensionality with respect to a change in recurrency is largest in the strongly recurrent regime.

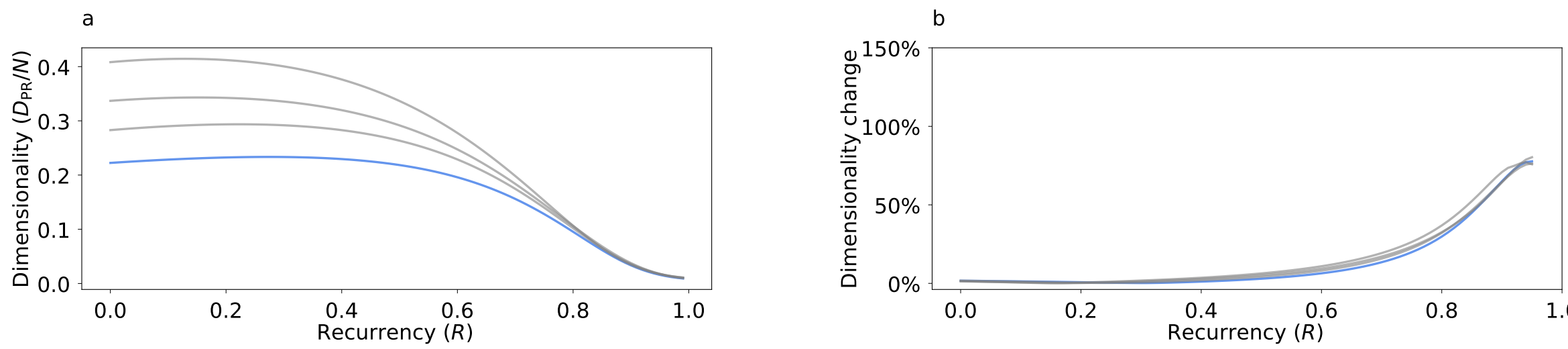

Figure S5: **Dimensionality and its sensitivity to recurrency in the presence of external inputs.** This figure shows the dependence of dimensionality on network recurrency in the presence of experimentally realistic external input. Network and input parameters are first inferred from experimental activity data from some example HMM states. Next the recurrency R of the fit models is modulated as in Fig. 3b,c of the main text, to assess its role in determining dimensionality. We find that while external input and distributed firing rates lead to an overall reduction in dimensionality, network recurrency further constrains the activity in the strongly recurrent regime and leads to a high sensitivity with respect to changes in R. This analysis thereby serves as a control to confirm that the relation between low/sensitive dimensionality and strong recurrency shown in Fig. 3b,c in the main text is also valid in the presence of external experimentally realistic inputs. For more details, see Sec. 3. This figure is analogous to Fig. 3b,c of the main text, but with external inputs. Correlated external inputs reduce the dimensionality of network activity, but the overall dependence on and sensitivity to recurrency R is similar to the case without external inputs. a) Dimensionality (normalized to the number of recorded neurons) of activity (including low-rank components) as a function of recurrency for network models from Fig. S4 that were fit to HMM states in visual cortex. Along the x-axis, only the recurrency of the fit model is changed, while all other network and input parameters are preserved. b) Relative dimensionality change as a function of recurrency for $\Delta R = -0.05$ (cf. Fig. 3c in main text) for fit network models in a. More details: see Sec. 3.

4 Field theory of spectral radius

4.1 Derivation of spectral radius in homogeneous networks with second-order motifs

4.1.1 Moment generating functional and disorder average

The analysis starts from the equation for the moment generating function of the time-averaged activity $\mathbf{X}$ (cf. (S16) for a network of linear coupled rate neurons, [10])

$$Z(\mathbf{J}) \quad = \quad |\det(\mathbf{1}-\mathbf{W})| \int_{\mathbf{X},\tilde{\boldsymbol{X}}} \exp\left(\tilde{\boldsymbol{X}}^T(\mathbf{1}-\mathbf{W})\boldsymbol{X}+\frac{1}{2}\tilde{\mathbf{X}}^T\boldsymbol{D}\tilde{\mathbf{X}}+\mathbf{J}\mathbf{X}\right)$$

In the following, we assume uniform noise covariance $\boldsymbol{D}=D\mathbb{1}$ as the noise amplitude and correlation does not determine stability. Our goal is to calculate the ensemble average $\langle Z(\mathbf{J})\rangle_W$ of the moment generating functional over all realizations of the effective connectivity $\mathbf{W}$. Dahmen et al. [10] showed that the $\mathbf{W}$-dependence of the prefactor $|\det(\mathbf{1}-\mathbf{W})|$ can, to a good approximation, be factorized such that

$$\langle Z(\mathbf{J})\rangle_W \quad \approx \quad \frac{\left\langle \tilde{Z}(\mathbf{J})\right\rangle_W}{\left\langle \tilde{Z}(0)\right\rangle_W},$$

where we defined $\tilde{Z}(\mathbf{J})=\int_{\mathbf{X},\tilde{\boldsymbol{X}}}\exp\left(\tilde{\boldsymbol{X}}^T(\mathbf{1}-\mathbf{W})\boldsymbol{X}+\frac{D}{2}\tilde{\mathbf{X}}^T\tilde{\mathbf{X}}+\mathbf{J}\mathbf{X}\right)$. To calculate the ensemble average we need to compute the integral $\int D\mathbf{W}=\prod_{i,j}\int \mathrm{d}W_{ij}$ of $\tilde{Z}(\mathbf{J})$ and weigh each contribution $\mathbf{W}$ according to its joint probability $p(\mathbf{W})$ with mean connection strengths $\langle W_{ij}\rangle=\mu_{ij}$ and covariance of connection strengths $\langle\langle W_{ik}W_{jl}\rangle\rangle=\frac{\Delta_{ijkl}}{N}$

$$\begin{aligned}
\left\langle \tilde{Z}(\mathbf{J})\right\rangle_W &= \int_{\mathbf{X},\tilde{\boldsymbol{X}}}\exp\left(-\tilde{\boldsymbol{X}}^T\boldsymbol{X}+\frac{D}{2}\tilde{\mathbf{X}}^T\tilde{\mathbf{X}}+\mathbf{J}\mathbf{X}\right)\\
&\times \int D\mathbf{W}p(\mathbf{W})\exp\left(\tilde{\boldsymbol{X}}^T\mathbf{W}\boldsymbol{X}\right)\\
&\approx \int_{\mathbf{X},\tilde{\boldsymbol{X}}}\exp\left(-\tilde{\boldsymbol{X}}^T\boldsymbol{X}+\frac{D}{2}\tilde{\mathbf{X}}^T\tilde{\mathbf{X}}+\mathbf{J}\mathbf{X}\right)\\
&\times \exp\left(\sum_{i,j}\mu_{ij}\tilde{X}_iX_j+\frac{1}{2N}\sum_{i,j,k,l}\Delta_{ijkl}\tilde{X}_i\tilde{X}_jX_kX_l\right).
\end{aligned}$$

Note that, when applying the often-used scaling of connection strengths $W_{ij}\sim 1/\sqrt{N}$ with network size N [13], we get $\mu_{ij}=\mathcal{O}(1/\sqrt{N})$ and $\Delta_{ijkl}=\mathcal{O}(1)$, and higher-order connectivity cumulants are suppressed, as we assume large networks.

4.1.2 Second order motifs

Second order motifs are encoded in the covariance Δ_{ijkl} as

$$\begin{aligned}
\Delta_{ijkl} &= \langle\langle W_{ik}W_{jl}\rangle\rangle N\\
&= \sigma^2\left(\delta_{ij}\delta_{kl}+\tau_{\mathrm{div}}(1-\delta_{ij})\delta_{kl}+\tau_{\mathrm{con}}(1-\delta_{kl})\delta_{ij}\right)\\
&+ \sigma^2\left(\tau_{\mathrm{chn}}\left(\delta_{il}(1-\delta_{jk})+\delta_{jk}(1-\delta_{il})\right)+\tau_{\mathrm{rec}}\delta_{il}\delta_{jk}\right),
\end{aligned}$$

such that we obtain a "'four-point interaction term"' of the form

$$\frac{1}{\sigma^2}\sum_{i,j,k,l}\Delta_{ijkl}\tilde{X}_i\tilde{X}_jX_kX_l = \underbrace{(1-\tau_{\text{div}}-\tau_{\text{con}})}_{=:A}\sum_i\tilde{X}_i\tilde{X}_i\sum_k X_kX_k + \underbrace{(\tau_{\text{rec}}-2\tau_{\text{chn}})}_{=:B}\left(\sum_i\tilde{X}_iX_i\right)^2 + \tau_{\text{div}}\left(\sum_i\tilde{X}_i\right)^2\sum_k X_kX_k + \tau_{\text{con}}\sum_i\tilde{X}_i\tilde{X}_i\left(\sum_k X_k\right)^2 + 2\tau_{\text{chn}}\sum_i\tilde{X}_iX_i\left(\sum_{j,k}\tilde{X}_jX_k\right), \tag{S23}$$

where we used $\sum_{i\neq j}X_iX_j=\left(\sum_i X_i\right)^2-\sum_i X_iX_i$. The first two lines of the above equation show a similar structure as in [14, 15]: inserting a field $Q=A\frac{\sigma^2}{N}\sum_k X_kX_k$ yields a renormalization of the noise amplitude D, while the insertion of a field $P=B\frac{\sigma^2}{N}\sum_k\tilde{X}_kX_k$ results in a renormalization of the propagator. The other lines $3-4$ in (S23) contain terms $R=\sum_k X_k$ and $S=\sum_i\tilde{X}_i$ which on expectation vanish exactly. Therefore, we could introduce the auxiliary fields R and S along with P and Q, but in the saddle-point approximation, we would find that $R^*=\sum_k\langle X_k\rangle=0$ and $S^*=\sum_i\left\langle\tilde{X}_i\right\rangle=0$ such that, at this level of approximation, we can neglect lines $3-4$ in (S23). We could also express line 5 using R and S, which would in principle lead to no contribution. However, line 5 will yield a renormalization of the response propagator, which is informative about outlier eigenvalues of the connectivity that are being induced by chain motifs, as will be discussed below. Introducing auxiliary fields P,Q and $T=\frac{\tau_{\text{chn}}\sigma^2}{N}\sum_{j,k}\tilde{X}_jX_k$ as well as their conjugate variables arising from the Fourier representation of the delta distribution yields

$$\begin{aligned}
e^{\frac{A\sigma^2}{2N}\sum_i\tilde{X}_i\tilde{X}_i\sum_k X_kX_k} &\sim \int_{Q,\tilde{Q}}e^{-\frac{N}{A\sigma^2}Q\tilde{Q}+\tilde{Q}\sum_k X_kX_k+\frac{1}{2}Q\sum_i\tilde{X}_i\tilde{X}_i},\\
e^{\frac{B\sigma^2}{2N}\left(\sum_i\tilde{X}_iX_i\right)^2} &\sim \int_P e^{-\frac{N}{2B\sigma^2}P^2+P\sum_i\tilde{X}_iX_i},\\
e^{\frac{\tau_{\text{chn}}\sigma^2}{N}\sum_i\tilde{X}_iX_i\sum_{j,k}\tilde{X}_jX_k} &\sim \int_{T,\tilde{T}}e^{-\frac{N}{\tau_{\text{chn}}\sigma^2}T\tilde{T}+\tilde{T}\sum_{j,k}\tilde{X}_jX_k+T\sum_i\tilde{X}_iX_i}
\end{aligned}$$

as well as the disorder-averaged generating function

$$\begin{aligned}
\left\langle\tilde{Z}(\mathbf{J})\right\rangle_W &\sim \int_{P,Q,\tilde{Q},T,\tilde{T}}e^{-\frac{N}{A\sigma^2}Q\tilde{Q}-\frac{N}{2B\sigma^2}P^2-\frac{N}{\tau_{\text{chn}}\sigma^2}T\tilde{T}+\ln\Omega(P,Q,T)},\\
\Omega\left(P,Q,T\right) &= \int_{\mathbf{X},\tilde{\mathbf{X}}}e^{-\tilde{\boldsymbol{X}}^T\boldsymbol{K}\boldsymbol{X}+\frac{D+Q}{2}\tilde{\mathbf{X}}^T\tilde{\mathbf{X}}+\tilde{Q}\boldsymbol{X}^T\boldsymbol{X}+\mathbf{J}\mathbf{X}},
\end{aligned}$$

where we define the inverse propagator

$$\boldsymbol{K}:=\left((1-P-T)\mathbf{1}-\boldsymbol{\mu}-\tilde{T}\{\mathbf{1}\}\right)$$

with $\mathbf{1}$ denoting the matrix of ones. As for fully random networks [10], we evaluate the exponent of the disorder-averaged generating function in saddle-point approximation, which yields

$$\tilde{Q}^* \sim \left\langle \tilde{\mathbf{X}}^T \tilde{\mathbf{X}} \right\rangle = 0$$

and, in addition,

$$Q^* = A\frac{\sigma^2}{N}\left\langle \boldsymbol{X}^T\boldsymbol{X}\right\rangle_{P^*,Q^*,T^*} = A\frac{\sigma^2}{N}\sum_i \left[\boldsymbol{K}^{-1}(D+Q^*)\boldsymbol{K}^{-T}\right]_{ii}, \tag{S24}$$

$$P^* = B\frac{\sigma^2}{N}\left\langle \tilde{\boldsymbol{X}}^T\boldsymbol{X}\right\rangle_{P^*,Q^*,T^*} = B\frac{\sigma^2}{N}\sum_i \left[\boldsymbol{K}^{-1}\right]_{ii}, \tag{S25}$$

$$T^* = \tau_{\text{chn}}\frac{\sigma^2}{N}\left\langle \tilde{\boldsymbol{X}}^T\{\mathbf{1}\}\boldsymbol{X}\right\rangle_{P^*,Q^*,T^*} = \tau_{\text{chn}}\frac{\sigma^2}{N}\sum_{i,j} \left[\boldsymbol{K}^{-1}\right]_{ij}, \tag{S26}$$

$$\tilde{T}^* = \tau_{\text{chn}}\frac{\sigma^2}{N}\left\langle \tilde{\boldsymbol{X}}^T\boldsymbol{X}\right\rangle_{P^*,Q^*,T^*}. \tag{S27}$$

In the following, we investigate the divergences in these equations as these give insight into the eigenvalues of the connectivity: The network activity becomes unstable when eigenvalues of the propagator diverge. From the propagator for the full linear network, $(\mathbf{1}-\boldsymbol{W})^{-1}$, this occurs when eigenvalues of $\boldsymbol{W}$ have amplitude > 1. So, we search for divergences in the saddle-point equations for the propagator of the disorder-averaged generating functional to find conditions on $\boldsymbol{W}$ that lead to instability.

4.1.3 Outlier eigenvalues of connectivity and relation to the propagator

While equations (S24)-(S27) hold for arbitrary mean connectivities, we in the following assume a homogeneous network with $\mu_{ij} = \mu$. In this case, the propagator drastically simplifies

$$\boldsymbol{K}^{-1} = (1-P^*-T^*)^{-1}\left(\mathbf{1} + \frac{\mu+\tilde{T}^*}{1-P^*-T^*-N(\mu+\tilde{T}^*)}\{\mathbf{1}\}\right) \tag{S28}$$

$$= (1-P^*-T^*)^{-1}\left(\mathbf{1} + \mathcal{O}(1/N)\{\mathbf{1}\}\right). \tag{S29}$$

From the saddle point equations, we then obtain

$$P^* = B\frac{\sigma^2}{N}\sum_i\left[\boldsymbol{K}^{-1}\right]_{ii} = \frac{(\tau_{\text{rec}}-2\tau_{\text{chn}})\,\sigma^2}{1-P^*-T^*} + \mathcal{O}(1/N),$$

$$\tilde{T}^* = \tau_{\text{chn}}\frac{\sigma^2}{N}\left\langle \tilde{\boldsymbol{X}}^T\boldsymbol{X}\right\rangle_{P^*,Q^*,T^*} = \frac{\tau_{\text{chn}}\sigma^2}{1-P^*-T^*} + \mathcal{O}(1/N),$$

$$T^* = \tau_{\text{chn}}\frac{\sigma^2}{N}\sum_{i,j}\left[\boldsymbol{K}^{-1}\right]_{ij} = \frac{\tau_{\text{chn}}\sigma^2}{1-P^*-T^*-N(\mu+\tilde{T}^*)} = \mathcal{O}(1/N),$$

so we can neglect T^* and obtain

$$P^* = \frac{1}{2} \pm \sqrt{\frac{1}{4} - B\sigma^2}, \tag{S30}$$

$$\tilde{T}^* = \frac{\tau_{\text{chn}}\sigma^2}{1-P^*}. \tag{S31}$$

Only the solution of (S30) with the minus sign is physically plausible as $P^* = 0$ for $B = 0$.

In order to extract information about eigenvalues of the effective connectivity $\boldsymbol{W}$, we next analyze the divergence of $\boldsymbol{K}^{-1}$: Both μ and σ depend linearly on the magnitude of the weights. Let's denote this scale with α and rescale $\mu \to \alpha\mu$ and $\sigma^2 \to \alpha^2\sigma^2$. The task is to find the scale of weights α that, for given parameters τ, μ, σ, leads to a divergence. In order to find that scale α, we need to solve the divergence criterion in (S28)

$$0 = 1 - P^*(\alpha) - N\mu\alpha + \frac{N\tau_{\text{chn}}\sigma^2\alpha^2}{1 - P^*(\alpha)} \tag{S32}$$

for α. Here we neglected T^* as this is $\mathcal{O}(1/N)$. The outlier eigenvalues λ_o of $\boldsymbol{W}$ are then obtained by knowing that all eigenvalues λ also scale with α, so $\lambda \to \alpha\lambda$, and the divergence takes place if one eigenvalue is at least one. So we investigate the limit case $\alpha\lambda = 1$. Therefore, the outlier eigenvalues are given by $\lambda_o = \alpha^{-1}$. The general solution of (S32) for homogeneous networks is

$$\begin{aligned}
\alpha &= \frac{(B - N\tau_{\text{chn}})\, N\mu}{2\left((B + N\tau_{\text{chn}})^2\,\sigma^2 + BN^2\mu^2\right)} \\
&\pm \frac{\sqrt{(-B + N\tau_{\text{chn}})^2\, N^2\mu^2 + 4\left((B + N\tau_{\text{chn}})^2\,\sigma^2 + BN^2\mu^2\right) N\tau_{\text{chn}}}}{2\left((B + N\tau_{\text{chn}})^2\,\sigma^2 + BN^2\mu^2\right)}
\end{aligned}$$

which reduces to $\lambda_o = \alpha^{-1} = N\mu + \mathcal{O}(\tau_{\text{rec}}/\sqrt{N})$ for $\tau_{\text{chn}} = 0$. This special case shows that reciprocal motifs only have a minor effect on the outlier eigenvalues of $\boldsymbol{W}$, such that the dominant contribution is $\lambda_o = N\mu$, which is known from purely random inhibitory networks. In summary, outlier eigenvalues of $\boldsymbol{W}$ are created by non-zero mean connectivity as well as chain motifs.

To avoid instabilities from outlier eigenvalues of $\boldsymbol{W}$, the mean connectivity μ must be either negative, or positive but smaller than $1/N$ (see discussion below on excitatory and inhibitory networks). The above equations show that not only μ appears in combination with N, but the same is true for τ_{chn}. Therefore, if outliers are not close to the instability line and therefore leading to divergences, then their effect is small and can be neglected.

Outlier eigenvalues of $\boldsymbol{W}$ and dimensionality If outlier eigenvalues of $\boldsymbol{W}$ are close to the instability line, then their corresponding mode dominates the dynamics. At the critical point, the dynamics is effectively one dimensional [10]. This illustrates that the outlier eigenvalues of $\boldsymbol{W}$ shape the dimensionality in networks, where they are close to the instability line. In networks where the bulk of eigenvalues is closer to the instability line - the scenario we focus on here - the dimensionality is shaped by the spectral radius.

Excitation-dominated networks For excitatory networks, we have $\mu_{ij} > 0$, so the eigenvalue due to the mean connectivity could yield a divergence, especially in large networks when we use the above mentioned scaling of weights as $1/\sqrt{(N)}$. Therefore, for stable single-population excitatory networks, one would rather employ a scaling that yields $\mu_{ij} \sim 1/N$. The outlier eigenvalues of $\boldsymbol{W}$ are furthermore affected by $P^*, T^*, \tilde{T}^*$, i.e. reciprocal and chain motifs (cf. (S28)). Therefore, in excitation-dominated networks, only chain and reciprocal motifs affect the dimensionality, but not convergent and divergent motifs, in line with findings in [16].

Inhibition-dominated networks In inhibitory networks we can apply the standard standard weight scaling $W_{ij} \sim 1/\sqrt{(N)}$, so we have $\mu \sim \mathcal{O}(1/\sqrt{N}) < 0$ and thus no divergences due to the mean connectivity. In this case, we can ignore effects from the mean connectivity. Let us for the remainder also assume that there is no instability due to the chain outlier. Then the $\mathcal{O}(1/N)$ correction in (S28) is small and we can approximate $\boldsymbol{K}^{-1} = \frac{1}{1-P^*}\mathbf{1}$.

4.1.4 Spectral radius and correlation functions

For the inhibitory network setting discussed above, we obtain from the saddle-point equations (S24) for the correlation function

$$Q^{*}=\frac{A\sigma^{2}}{(1-P^{*})^{2}-A\sigma^{2}}D. \tag{S33}$$

If the spectral radius R of effective connectivity approaches one, then one eigendirection of the dynamics becomes unstable, and as a consequence correlations diverge. Since Q^{*} measures correlations (cf. (S24)), the aim is to find a divergence of Q^{*}. From the equation above follows the condition

$$(1-P^{*})^{2}-A\sigma^{2}=0, \tag{S34}$$

which has to be rewritten in the following general form

$$R(\sigma^{2},\tau_{\mathrm{con}},\tau_{\mathrm{div}},\tau_{\mathrm{chn}},\tau_{\mathrm{rec}})=1 \tag{S35}$$

to infer the functional dependence of the spectral radius on the various second order statistics. Eigenvalues of $\boldsymbol{W}$ scale with the strength of connections W_{ij} and therefore does the standard deviation σ. Hence, R needs to be proportional to σ. Starting from (S34), we get

$$P^{*}+\sqrt{A}\sigma=1 \tag{S36}$$

which, due to the fact that P contains a square root, is not yet of the desired form (S35). Inserting (S30), squaring and reordering the resulting equation yields the desired form

$$\frac{A+B}{\sqrt{A}}\sigma=1\,. \tag{S37}$$

The spectral radius can then be read off as

$$R=\frac{A+B}{\sqrt{A}}\sigma=\frac{1-\tau_{\mathrm{div}}-\tau_{\mathrm{con}}+\tau_{\mathrm{rec}}-2\tau_{\mathrm{chn}}}{\sqrt{1-\tau_{\mathrm{div}}-\tau_{\mathrm{con}}}}\sigma. \tag{S38}$$

The spectral radius is therefore affected by all motifs.

4.2 Generalization to networks with multiple populations

In the following, we consider networks with multiple (M) populations where the connection statistics to neurons only depends on the presynaptic populations. The latter constraint significantly reduces the parameter space. This is possible for all second order statistics except for the reciprocal motifs, as for the latter the postsynaptic neuron of the first connection is the presynaptic neuron of the second connection. For such connection statistics, we obtain

$$\begin{aligned}
\Delta_{ijkl} &= \langle\langle W_{ik}W_{jl}\rangle\rangle\, N\\
&= \sigma_{k}^{2}\delta_{ij}\delta_{kl}+\kappa_{\mathrm{div},k}(1-\delta_{ij})\delta_{kl}+\kappa_{\mathrm{con},kl}(1-\delta_{kl})\delta_{ij}\\
&+ \kappa_{\mathrm{chn},lk}\delta_{il}(1-\delta_{jk})+\kappa_{\mathrm{chn},kl}\delta_{jk}(1-\delta_{il})+\kappa_{\mathrm{rec},kl}\delta_{il}\delta_{jk}\\
&= \delta_{ij}\delta_{kl}\left(\sigma_{k}^{2}-\kappa_{\mathrm{div},k}-\kappa_{\mathrm{con},kl}\right)\\
&+ \delta_{il}\delta_{jk}\left(\kappa_{\mathrm{rec},kl}-\kappa_{\mathrm{chn},kl}-\kappa_{\mathrm{chn},lk}\right)\\
&+ \text{terms containing a single}\,\delta\,,
\end{aligned}$$

where we can neglect the terms containing a single Kronecker delta (see discussion of (S23) above). Inserted in the action, we obtain a term

$$
\begin{aligned}
\sum_{i,j,k,l}\Delta_{ijkl}\tilde{X}_i\tilde{X}_jX_kX_l &= \sum_{i,k}\left(\sigma_k^2-\kappa_{\text{div},k}-\kappa_{\text{conv},kk}\right)\tilde{X}_i\tilde{X}_iX_kX_k \\
&+ \sum_{k,l}\left(\kappa_{\text{rec},kl}-\kappa_{\text{chain},kl}-\kappa_{\text{chain},lk}\right)\tilde{X}_kX_k\tilde{X}_lX_l \\
&= \sum_i \tilde{X}_i\tilde{X}_i\sum_\alpha \underbrace{\left(\sigma_\alpha^2-\kappa_{\text{div},\alpha}-\kappa_{\text{conv},\alpha\alpha}\right)}_{A_\alpha}\sum_{k\in\alpha}X_kX_k \\
&+ \sum_{\alpha,\beta}\underbrace{\left(\kappa_{\text{rec},\alpha\beta}-\kappa_{\text{chain},\alpha\beta}-\kappa_{\text{chain},\beta\alpha}\right)}_{B_{\alpha\beta}=B_{\beta\alpha}}\sum_{k\in\alpha}\tilde{X}_kX_k\sum_{l\in\beta}\tilde{X}_lX_l
\end{aligned}
$$

where the sum over α and β run over all M populations. Now we insert auxiliary fields

$$
\begin{aligned}
Q_\alpha &= \frac{1}{N_\alpha}\sum_{k\in\alpha}X_kX_k\,, \\
P_\alpha &= \frac{1}{N_\alpha}\sum_{k\in\alpha}\tilde{X}_kX_k\,,
\end{aligned}
$$

and write

$$
\begin{aligned}
1 &\sim \int DQ_\alpha\int D\tilde{Q}_\alpha\,\exp\left(-N_\alpha Q_\alpha\tilde{Q}_\alpha+\tilde{Q}_\alpha\textstyle\sum_{k\in\alpha}X_kX_k\right)\,, \\
1 &\sim \int DP_\alpha\int D\tilde{P}_\alpha\,\exp\left(-N_\alpha P_\alpha\tilde{P}_\alpha+\tilde{P}_\alpha\textstyle\sum_{k\in\alpha}\tilde{X}_kX_k\right)\,,
\end{aligned}
$$

so that we get a disorder-averaged generating function (for simplicity we set $\boldsymbol{\mu}=0$ which is a good approximation as long as $\boldsymbol{\mu}$ is not tuned to drive the system close to instability, see above)

$$
\begin{aligned}
\left\langle\tilde{Z}(\mathbf{J})\right\rangle_W &\sim \int\boldsymbol{DP}\int\boldsymbol{DQ}\exp\left(S(\boldsymbol{P},\boldsymbol{Q})\right) \\
S(\boldsymbol{P},\boldsymbol{Q}) &= -\sum_\alpha\left(N_\alpha Q_\alpha\tilde{Q}_\alpha+N_\alpha P_\alpha\tilde{P}_\alpha\right)+\frac{1}{2N}\sum_{\alpha,\beta}B_{\alpha\beta}N_\alpha P_\alpha N_\beta P_\beta+\ln\Omega\left(\boldsymbol{P},\boldsymbol{Q}\right) \\
\Omega\left(\boldsymbol{P},\boldsymbol{Q}\right) &= \int D\mathbf{X}\int D\tilde{\boldsymbol{X}}\,\exp\left(-\tilde{\boldsymbol{X}}^T\left(\mathbf{1}-\tilde{\boldsymbol{P}}\right)\boldsymbol{X}+\frac{1}{2}\left(D+\frac{\sum_\alpha N_\alpha Q_\alpha A_\alpha}{N}\right)\tilde{\mathbf{X}}^T\tilde{\mathbf{X}}+\boldsymbol{X}^T\tilde{\boldsymbol{Q}}\boldsymbol{X}+\mathbf{J}\mathbf{X}\right)\,.
\end{aligned}
$$

As for fully random networks, we evaluate the exponent of the disorder-averaged generating function in saddle-point approximation, which yields

$$
\tilde{Q}_\alpha^*\sim\left\langle\tilde{X}_i\tilde{X}_i\right\rangle=0
$$

and, in addition,

$$
Q_\alpha^* = \frac{1}{N_\alpha}\left\langle\sum_{k\in\alpha}X_kX_k\right\rangle_{P^*,Q^*} = \frac{1}{N_\alpha}\sum_{k\in\alpha}\left[\left(\mathbf{1}-\tilde{\boldsymbol{P}}^*\right)^{-1}\left(D+\frac{\sum_\gamma N_\gamma Q_\gamma A_\gamma}{N}\right)\left(\mathbf{1}-\tilde{\boldsymbol{P}}^*\right)^{-T}\right]_{kk} = \frac{D+\frac{\sum_\gamma N_\gamma Q_\gamma A_\gamma}{N}}{(1-\tilde{P}_\alpha^*)^2} \tag{S39}
$$

$$
P_\alpha^* = \frac{1}{N_\alpha}\left\langle\sum_{k\in\alpha}\tilde{X}_kX_k\right\rangle_{P^*,Q^*} = \frac{1}{N_\alpha}\sum_{k\in\alpha}\left[\left(\mathbf{1}-\tilde{\boldsymbol{P}}^*\right)^{-1}\right]_{kk} = \frac{1}{1-\tilde{P}_\alpha^*} \tag{S40}
$$

$$
\tilde{P}_\alpha^* = \frac{1}{N}\sum_\beta B_{\alpha\beta}N_\beta P_\beta^* \tag{S41}
$$

Solving (S39) yields

$$Q^*_\alpha \;=\; \sum_\beta M_{\alpha,\beta} P^{*2}_\alpha D,$$

with $M_{\alpha,\beta} = \delta_{\alpha,\beta} - P^{*2}_\alpha \frac{N_\beta}{N} A_\beta$. We obtain a divergence if $\det(M) = 0$. Since M is the identity matrix shifted by an outer product, we can use the matrix determinant lemma to calculate the determinant of M:

$$\det M = 1 - \sum_\gamma \frac{N_\gamma}{N} A_\gamma P^{*2}_\gamma = 0 \tag{S42}$$

which shows a divergence, similar as in the case for the single-population network above. In fact, the divergence expression reduces to that of the single-population network for homogeneous $\tilde{P}^*_\alpha = \tilde{P}^*$, which follows from homogeneous $B_{\alpha\beta} = B$ for all α, β. If the latter equality does not hold, then finding the spectral radius amounts to solving a set of coupled nonlinear equations, which gives very convoluted expressions. We therefore here approximate $B_{\alpha\beta} \approx \frac{1}{M} \sum_\alpha B_{\alpha\beta} = B_\beta$ and obtain from (S40) and (S41)

$$P^* = \frac{1}{1 - \sum_\beta B_\beta \frac{N_\beta}{N} P^*} \,. \tag{S43}$$

Note that an alternative approximation would be a weighted average $B_{\alpha\beta} \approx \sum_{\alpha,\beta} \frac{N_\alpha}{N} B_{\alpha\beta} = B_\beta$ yielding qualitatively similar results. Equations (S42) and (S43) then have the same form as in the single-population network and can be solved analogously. Redefining

$$\begin{aligned}
\sigma^2 &= \sum_\alpha \frac{N_\alpha}{N} \sigma^2_\alpha \,, \\
\tau_{\text{div}} &= \frac{\kappa_{\text{div}}}{\sigma^2} = \frac{\sum_\alpha \frac{N_\alpha}{N} \kappa_{\text{div},\alpha}}{\sum_\alpha \frac{N_\alpha}{N} \sigma^2_\alpha} \,, \\
\tau_{\text{con}} &= \frac{\kappa_{\text{con}}}{\sigma^2} = \frac{\sum_\alpha \frac{N_\alpha}{N} \kappa_{\text{con},\alpha\alpha}}{\sum_\alpha \frac{N_\alpha}{N} \sigma^2_\alpha} \,, \\
\tau_{\text{rec}} &= \frac{\kappa_{\text{rec}}}{\sigma^2} = \frac{\frac{1}{M} \sum_{\alpha,\beta} \frac{N_\beta}{N} \kappa_{\text{rec},\alpha\beta}}{\sum_\alpha \frac{N_\alpha}{N} \sigma^2_\alpha} \,, \\
\tau_{\text{chn}} &= \frac{\kappa_{\text{chn}}}{\sigma^2} = \frac{\frac{1}{2M} \sum_{\alpha,\beta} \frac{N_\alpha + N_\beta}{N} \kappa_{\text{chn},\alpha\beta}}{\sum_\alpha \frac{N_\alpha}{N} \sigma^2_\alpha}
\end{aligned}$$

as combinations of motif statistics of multiple populations, we obtain the same expression for the spectral radius

$$R = \frac{1 - \tau_{\text{div}} - \tau_{\text{con}} + \tau_{\text{rec}} - 2\tau_{\text{chn}}}{\sqrt{1 - \tau_{\text{div}} - \tau_{\text{con}}}} \sigma. \tag{S44}$$

4.3 From covariances of connections to motif abundances

Due to the limited quantity of data on the effective strengths of synapses in the synaptic physiology datasets and the possible deviations of effective strengths between in-vivo and in-vitro settings, we quantified only binarized motif statistics in the data. Here we demonstrate how to translate between cumulants κ and binarized motif statistics for an example network of one excitatory and one inhibitory population. In the framework above all excitatory cells have the same strengths $J_E = \rho_E J$ and all inhibitory synapses have the same strengths $J_I = -\rho_I g J$. Here, ρ_E and ρ_I are scaling factor to individually modulate the effective strength of excitation and inhibition around the reference value 1,

which is used if not stated otherwise. In excitatory-inhibitory networks, the typically larger number of excitatory neurons is often compensated by a larger strength of inhibitory synapses (see [17, 2]). In going from covariances of connections to correlation coefficients τ, the overall synaptic strength J drops out, but the relative strengths given by $-g$, ρ_E and ρ_I remain. For excitatory connection probabilities p_E, inhibitory connection probabilities p_I and a ratio $\gamma = N_I/N_E$ between inhibitory and excitatory population sizes, we obtain

$$
\begin{aligned}
\sigma^2 &= \frac{N_E}{N}\rho_E^2 J^2 \hat{\sigma}_E^2 + \frac{N_I}{N}\rho_I^2 g^2 J^2 \hat{\sigma}_I^2 \,, \\
\tau_{\mathrm{div}} &= \frac{\rho_E^2 \hat{\sigma}_E^2 \tau_{\mathrm{div},E} + \gamma \rho_I^2 g^2 \hat{\sigma}_I^2 \tau_{\mathrm{div},I}}{\rho_E^2 \hat{\sigma}_E^2 + \gamma \rho_I^2 g^2 \hat{\sigma}_I^2} \,, \\
\tau_{\mathrm{con}} &= \frac{\rho_E^2 \hat{\sigma}_E^2 \tau_{\mathrm{con},EE} + \gamma g^2 \rho_I^2 \hat{\sigma}_I^2 \tau_{\mathrm{con},II}}{\rho_E^2 \hat{\sigma}_E^2 + \gamma \rho_I^2 g^2 \hat{\sigma}_I^2} \,, \\
\tau_{\mathrm{rec}} &= \frac{1}{2}\frac{\rho_E^2 \hat{\sigma}_E^2 \tau_{\mathrm{rec},EE} - (1+\gamma) g \rho_E \rho_I \sqrt{\hat{\sigma}_E^2 \hat{\sigma}_I^2}\,\tau_{\mathrm{rec},EI} + \gamma \rho_I^2 g^2 \hat{\sigma}_I^2 \tau_{\mathrm{rec},II}}{\rho_E^2 \hat{\sigma}_E^2 + \gamma \rho_I^2 g^2 \hat{\sigma}_I^2} \,, \\
\tau_{\mathrm{chn}} &= \frac{1}{2}\frac{\rho_E^2 \hat{\sigma}_E^2 \tau_{\mathrm{chn},EE} - \frac{1+\gamma}{2} g \rho_E \rho_I \sqrt{\hat{\sigma}_E^2 \hat{\sigma}_I^2}\,(\tau_{\mathrm{chn},EI} + \tau_{\mathrm{chn},IE}) + \gamma \rho_I^2 g^2 \hat{\sigma}_I^2 \tau_{\mathrm{chn},II}}{\rho_E^2 \hat{\sigma}_E^2 + \gamma \rho_I^2 g^2 \hat{\sigma}_I^2} \,,
\end{aligned}
$$

with $\hat{\sigma}_E^2 = p_E(1-p_E)$ and $\hat{\sigma}_I^2 = p_I(1-p_I)$.

5 Constraints on motif abundances and algorithms for network construction

Given that the spectral radius is affected by all motifs, we now ask the question, which abundances of motifs are possible. We already saw one restriction above, namely that in homogeneous networks τ_{chn} must be either on the order $\mathcal{O}(1/N)$ or negative in order to yield stable dynamics (see discussion on outlier eigenvalues above). However, there are more restrictions on the motif abundances coming from the fact that the covariance matrix of connections weights must be positive definite: no negative eigenvalues are allowed. Reciprocal motifs always yield positive definite covariance matrices, therefore all values $\tau_{\mathrm{rec}} \in [-1, 1]$ are possible.

The situation is different for divergent and convergent motifs: while any positive value $\tau \in [0, 1]$ is possible, only negative values $> -1/N$ are allowed. An intuitive explanation for this is that for divergent and convergent motifs any connection is not only part of one possible motif - as it is the case for reciprocal motifs - but of N motifs (here we also count motifs with self-connections). In order to have negative values for τ_{div} or τ_{con}, pairs of connections must be negatively correlated. For Gaussian networks, this can be achieved by drawing a common Gaussian distributed number α_i and setting $W_{ij} \leftarrow W_{ij} + \alpha_i$ and $W_{ik} \leftarrow W_{ik} - \alpha_i$ for one $k \neq i$. In order for the connection W_{ij} to be negatively correlated to all $k \neq i$, one would need to draw a new number α_i for each neuron k, therefore in total N numbers. These N numbers of course also contribute to the variance of connection W_{ij}. Therefore each number α_i can only have a variance of order $1/N$ and therefore τ can only be of order $1/N$. One might think that the same situation applies for positive τ. However, in this case it is possible to draw a single number α_i for all k. This also creates a higher-order motif, but also leads to the fact that all pairs of connections ending at neuron i are positively correlated. Therefore, any value $\tau \in [0, 1]$ is possible for convergent motifs. The same rationale holds for divergent motifs.

We find again another situation for chain motifs: if one wants to include chains only, then the range of τ_{chn} is restricted to values $|\tau_{\mathrm{chn}}| < 1/N$. For positive τ_{chn}, this is in line with the stability issue of the outlier eigenvalue above. But for negative τ_{chn} we find the same argument as for convergent and divergent motifs above: each connection is part of $\mathcal{O}(N)$ chains. Negative correlations of sizable strength can therefore not be realized. However, there is another possibility. If one allows creation of divergent, convergent and reciprocal motifs while creating chains, one possibility is to draw one random number α_i

for all connections that start or end at neuron i. This creates chain correlations, but also divergent, convergent and reciprocal ones. In this case, one can obtain a positive definite covariance matrix for larger values of τ_{chn}, but large positive values are additionally ruled out by the outlier instability. Therefore, only one possible case remains, where a single α_i is drawn for all connections starting and ending at neuron i, but $W_{ij} \leftarrow W_{ij} + \alpha_i$ and $W_{ji} \leftarrow W_{ji} - \alpha_i$ for all j to create negative chain correlations.

The above considerations lead to the following algorithms for the creation of Gaussian networks with desired second-order statistics (see also [18]): We draw weights

$$W_{ij} = \mu + A\nu_{ij} + B\nu_{ji} + C\eta_i + D\eta_j$$

with independent and identically distributed variables $\nu_{ij} \overset{i.i.d.}{\sim} \mathcal{N}(0,1)$ and $\eta_i \overset{i.i.d.}{\sim} \mathcal{N}(0,1)$, so that coefficients A, B, C, D encode covariances between weights

$$\begin{aligned}
\langle W_{ij}W_{kl}\rangle - \langle W_{ij}\rangle\langle W_{kl}\rangle &= \langle (A\nu_{ij} + B\nu_{ji} + C\eta_i + D\eta_j)\,(A\nu_{kl} + B\nu_{lk} + C\eta_k + D\eta_l)\rangle \\
&= \left(A^2 + B^2\right)\delta_{ik}\delta_{jl} + 2AB\delta_{il}\delta_{jk} \\
&+ C^2\delta_{ik} + D^2\delta_{jl} + CD\delta_{il} + CD\delta_{jk} \\
&= \underbrace{\left(A^2 + B^2 + C^2 + D^2\right)}_{=\sigma^2/N}\delta_{ik}\delta_{jl} + \underbrace{(2AB + 2CD)}_{=\sigma^2\tau_{\mathrm{rec}}/N}\delta_{il}\delta_{jk} \\
&+ \underbrace{C^2}_{=\sigma^2\tau_{\mathrm{con}}/N}\delta_{ik}(1-\delta_{jl}) + \underbrace{D^2}_{=\sigma^2\tau_{\mathrm{div}}/N}\delta_{jl}(1-\delta_{ik}) \\
&+ \underbrace{CD}_{=\sigma^2\tau_{\mathrm{chn}}/N}\left(\delta_{il}(1-\delta_{jk}) + \delta_{jk}(1-\delta_{il})\right)\,.
\end{aligned}$$

Solving the above relations yields equations for the coefficients A, B, C, D in terms of the motif statistics:

$$\begin{aligned}
A &= \frac{\sigma}{2\sqrt{N}}\sqrt{1-\tau_{\mathrm{con}}-\tau_{\mathrm{div}}}\left(\sqrt{1+U} + \sqrt{1-U}\right)\,, \\
B &= \frac{\sigma}{2\sqrt{N}}\sqrt{1-\tau_{\mathrm{con}}-\tau_{\mathrm{div}}}\left(\sqrt{1+U} - \sqrt{1-U}\right)\,, \\
C &= \pm\frac{\sigma}{\sqrt{N}}\sqrt{\tau_{\mathrm{con}}}\,, \\
D &= \pm\frac{\sigma}{\sqrt{N}}\sqrt{\tau_{\mathrm{div}}}\,,
\end{aligned}$$

with

$$U = \frac{\tau_{\mathrm{rec}} - 2\tau_{\mathrm{chn}}}{1 - \tau_{\mathrm{con}} - \tau_{\mathrm{div}}}\,.$$

We obtain the restriction $\tau_{\mathrm{chn}} = \pm\sqrt{\tau_{\mathrm{con}}\tau_{\mathrm{div}}}$, i.e. chain motifs can only be created along with convergent and divergent motifs, as previously reported in [19]. To avoid positive outlier eigenvalues of $\boldsymbol{W}$, in the numerical simulations presented in the main text, we mostly chose $C = \frac{\sigma}{\sqrt{N}}\sqrt{\tau_{\mathrm{con}}}$, and $D = -\frac{\sigma}{\sqrt{N}}\sqrt{\tau_{\mathrm{div}}}$ such that $\tau_{\mathrm{chn}} = -\sqrt{\tau_{\mathrm{con}}\tau_{\mathrm{div}}} < 0$. Since values for U are restricted to the interval $[-1, 1]$ we furthermore chose $\tau_{\mathrm{rec}} = 2\tau_{\mathrm{chn}}$ and $\tau_{\mathrm{con}} = \tau_{\mathrm{div}}$ to increase the range of possible values τ_{chn}.

For excitatory-inhibitory networks, the coefficients receive population indices $\alpha, \beta, \gamma, \delta \in \{E, I\}$. For $i \in \alpha$, $j \in \beta$, $k \in \gamma$ and $l \in \delta$, we draw weights $W_{ij} = \mu + A_{\alpha\beta}\nu_{ij} + B_{\alpha\beta}\nu_{ji} + C_\beta\eta_i + D_\beta\eta_j$ and obtain the following coefficients

$$
\begin{aligned}
A_{\alpha\beta} &= \frac{\sigma_\beta}{2\sqrt{N}}\sqrt{1-\tau_{\mathrm{con},\beta\beta}-\tau_{\mathrm{div},\beta}}\left(\sqrt{1+U_{\alpha\beta}}+\sqrt{1-U_{\alpha\beta}}\right)\\
B_{\alpha\beta} &= \frac{\sigma_\beta}{2\sqrt{N}}\sqrt{1-\tau_{\mathrm{con},\beta\beta}-\tau_{\mathrm{div},\beta}}\left(\sqrt{1+U_{\alpha\beta}}-\sqrt{1-U_{\alpha\beta}}\right)\\
C_\alpha &= \pm\frac{\sigma_\alpha}{\sqrt{N}}\sqrt{\tau_{\mathrm{con},\alpha\alpha}}\,,\\
D_\alpha &= \pm\frac{\sigma_\alpha}{\sqrt{N}}\sqrt{\tau_{\mathrm{div},\alpha}}\,,
\end{aligned}
$$

with

$$
U_{\alpha\beta} = (-1+2\delta_{\alpha\beta})\,\frac{\tau_{\mathrm{rec},\alpha\beta}-\tau_{\mathrm{chn},\alpha\beta}-\tau_{\mathrm{chn},\beta\alpha}}{\sqrt{1-\tau_{\mathrm{con},\alpha\alpha}-\tau_{\mathrm{div},\alpha}}\sqrt{1-\tau_{\mathrm{con},\beta\beta}-\tau_{\mathrm{div},\beta}}}\,.
$$

Note that the overall minus sign in U_{EI} arises from a relative minus sign between correlation coefficients $\tau_{\mathrm{rec},EI}$ of motif abundances (binarized statistics) and covariances $\kappa_{\mathrm{rec},EI}$ that include synaptic weights, i.e. $\kappa_{\mathrm{rec},EI} = -gJ^2\left(\sqrt{\hat{\sigma}_E^2\hat{\sigma}_I^2}\tau_{\mathrm{rec},EI}\right) = -\sigma_E\sigma_I\tau_{\mathrm{rec},EI}$. The same holds true for chain motifs between excitatory and inhibitory neurons, $\kappa_{\mathrm{chn},\alpha\beta} = -\sigma_E\sigma_I\tau_{\mathrm{rec},\alpha\beta}$ for $\alpha \neq \beta$. We obtain the restriction $\tau_{\mathrm{chn},\alpha\alpha} = -\sqrt{\tau_{\mathrm{con},\alpha\alpha}\tau_{\mathrm{div},\alpha}}$ and $\tau_{\mathrm{chn},\alpha\beta} = +\sqrt{\tau_{\mathrm{con},\alpha\alpha}\tau_{\mathrm{div},\beta}}$ for $\alpha \neq \beta$. Networks generated with this algorithm allow us to validate the theory for the spectral radius in multi-population networks with population-specific motif statistics (cf. Sec. 4.2, Fig. S6).

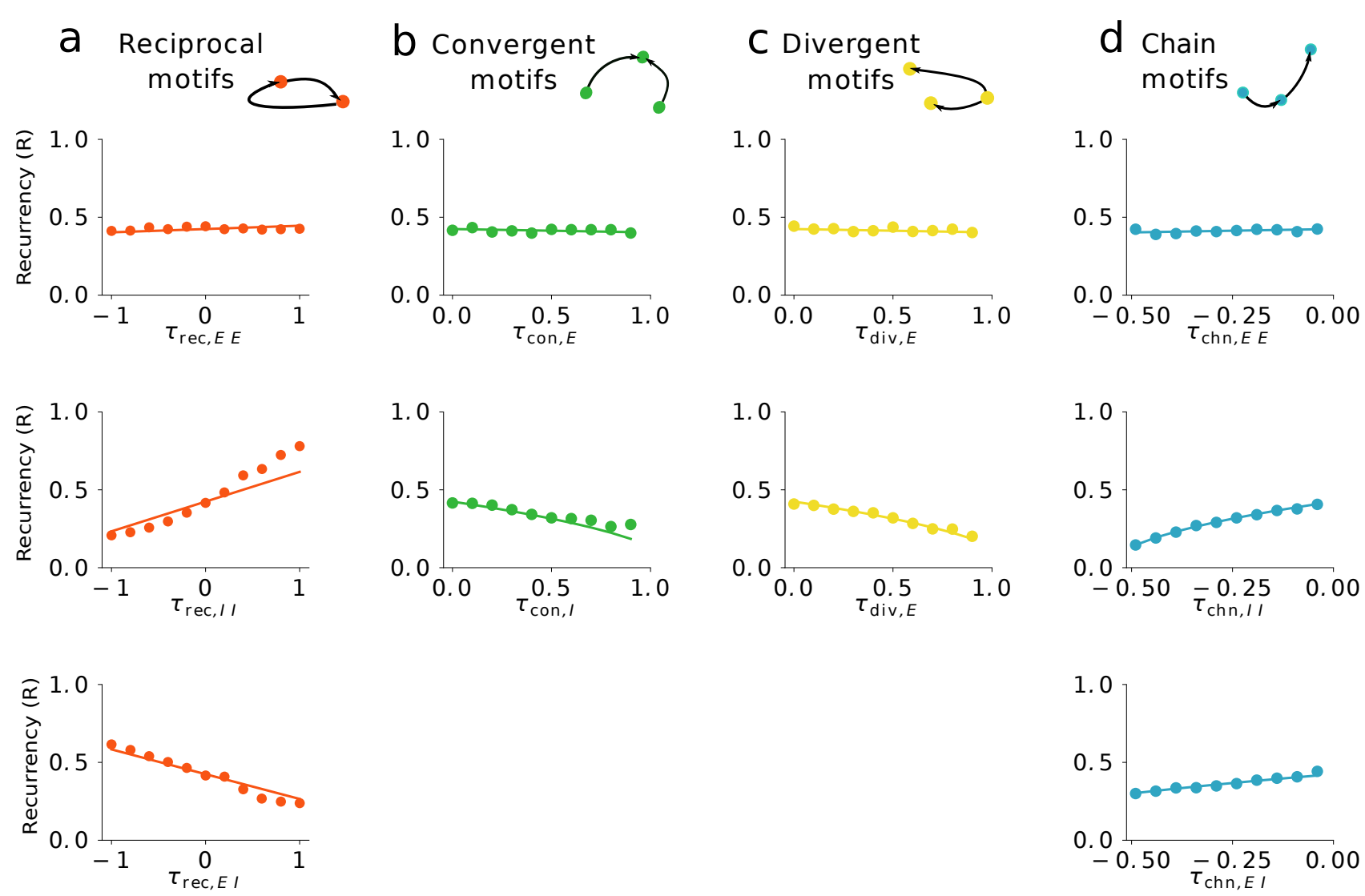

Figure S6: **Numerical validation of theory for recurrency (spectral radius) in excitatory-inhibitory networks with population-specific motif abundances.** This figure shows a validation of our theoretical predictions (see Sec. 4.2) for the recurrency (spectral radius) of excitatory-inhibitory networks with population specific abundances of reciprocal, convergent, divergent and chain motifs, against numerical analyses of connectivity matrices generated with the algorithm described in Sec. 5. The figure is analogous to Fig. 4a of the main text, but for the case of two populations. Recurrency (spectral radius) R as a function of abundances of excitatory and inhibitory a) recurrent motifs, b) convergent motifs, c) divergent motifs, and d) chain motifs. Only one parameter is varied at a time with all other motif abundances set to zero. Theoretical predictions are given by solid curves, numerical evaluations of single network realizations are shown by colored markers. Cf. Sec. 5 for network construction algorithms. The approximate theory correctly predicts the influence of changes in abundance of each type of motif on the spectral radius. In particular, an over-representation of reciprocal EE and II motifs increases the spectral radius while an over-representation of reciprocal EI motifs decreases the spectral radius. These effects are the dominant contributions to the results obtained from the synaptic physiology dataset. More details: see Sec. 4.2ff.

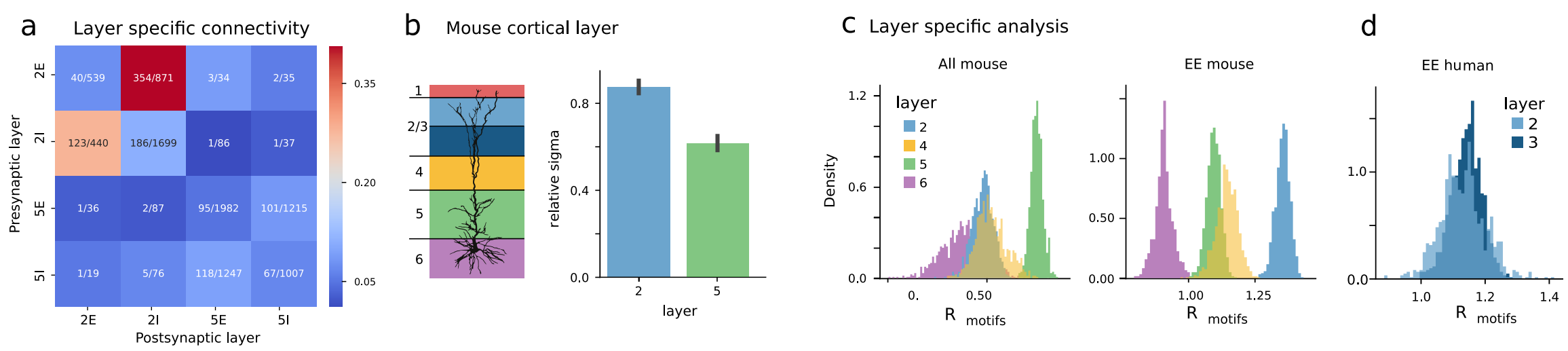

Figure S7: **Layer-resolved analysis of recurrency.** a) Statistics of mouse connectivity across layers for excitatory and inhibitory synapses (synapses identified / synapses probed). b) Relative first-order recurrence strength σ in layer 2 and layer 5 mouse cortex, computed from paired-patch connectivity and normalized to the pooled layers 2+5 reference (see Suppl. Mat. Sec. 8.1). Bars summarize n=1,000 Monte Carlo draws propagating binomial uncertainty in connection probabilities; error bars ± 1 s.d. c) Inferred R_{motifs} for layer specific neural populations. The computation includes synapses of a given layer that are both excitatory and inhibitory (left) or only excitatory (right). Same statistics utilized in panel b. d) Layer-wise estimation of R_{motifs} for EE-only motifs in human. Bootstrap distribution n=1,000 resamples, 80% of experiments per draw.

6 Reanalysis of synaptic physiology data from Song et al.

We analyzed data from [20] on motif abundances in excitatory connections of rat visual cortex and evaluated their influence on R_{motifs} in an analogous way as we did for the Allen synaptic physiology dataset [21]. The probability of occurrence for each motif type is given by the ratio between the observed occurrences and the possible occurrences of motifs, each summed across all simultaneous patch clamp recordings of triplets of neurons. The data in [20] is presented in their Fig. 4b in terms of 16 different patterns which each contain possibly multiple of the motifs discussed here. For example, pattern 12 contains one reciprocal motif, one divergent motif, two convergent motifs and two chain motifs. We thus calculated the probability of each motif in terms of the occurrences M_p of patterns $p \in \{1, 2, ..., 16\}$, given by the numbers above the bars in Fig. 4b of [20]), and the number of occurrences of this particular motif in each pattern:

$$p_{\text{motif}} = \frac{\sum_{\text{triplets}} \text{observed} \quad \text{motifs}}{\sum_{\text{triplets}} \text{possible} \quad \text{motifs}} = \frac{\sum_{p=1}^{16} M_p \cdot \#\text{motifs} \quad \text{in} \quad \text{p}}{\sum_{p=1}^{16} M_p \cdot \#\text{possible} \quad \text{motifs} \quad \text{in} \quad \text{triplet}}. \tag{S45}$$

This yielded values $\tau_{\text{rec}} = 0.40, \tau_{\text{chn}} = 0.02, \tau_{\text{div}} = 0.02, \tau_{\text{con}} = 0.04$ and $R_{\text{motifs}} = 1.34$, which are broadly consistent with our findings on excitatory connections from the synaptic physiology dataset [21].

7 Layer- and cell-type resolved analysis of synaptic physiology dataset

In this analysis we considered all synapses and computed their statistics across either layers (Fig. S7) or cell types (Fig. S8, Fig. S10).

7.1 Connection probabilities and motif abundances

First, we observed that neurons within and across the excitatory and inhibitory populations of layer 2 are on average more connected than those of layer 5 (Fig. S7a). Second, as in [21], we noted that PV cells are more connected to pyramidal cells, compared with SST and VIP cells (Fig. S8a). We furthermore separately quantified cell-type specific motifs and found strong differences across the analyzed cell types (Fig. S9). Next, we combined these statistics to determine properties of the circuit recurrency.

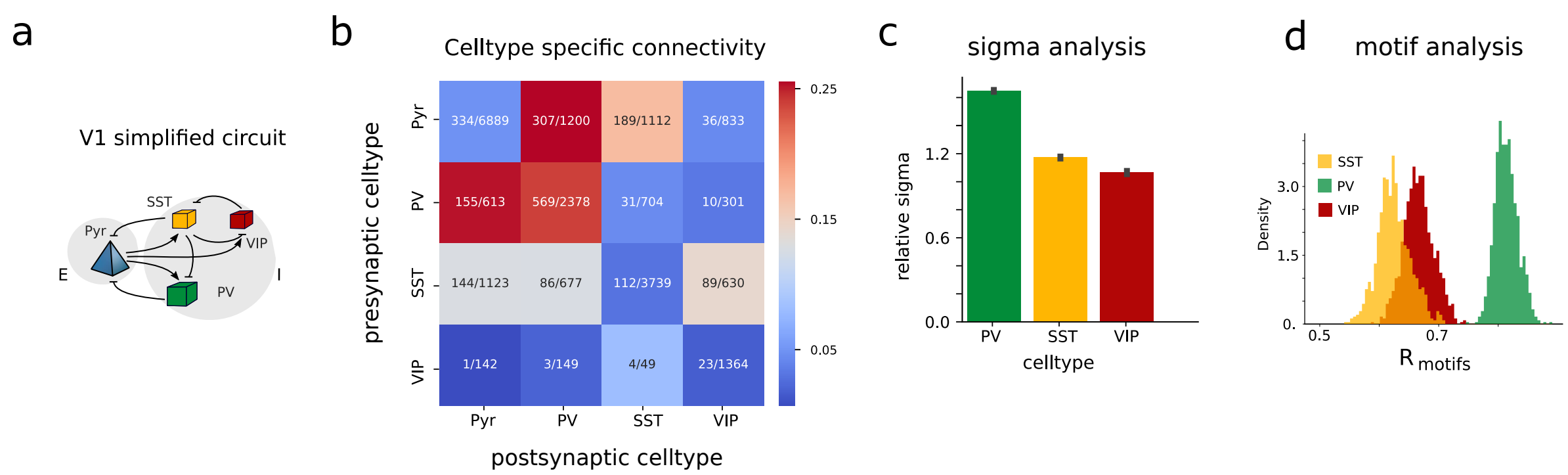

Figure S8: **Cell-type-resolved analysis of recurrency.** a) Sketch of simplified V1 circuit with distinction between different inhibitory cell types. b) Statistics of mouse connectivity across cell types (synapses identified / synapses probed). c) Relative strength of connectivity for mouse V1 circuit with multiple cell types ($\sigma_{\mathrm{X}}/\sigma_{\mathrm{PV+SST+VIP}}$ for $X \in \{PV, SST, VIP\}$). Statistics computed from paired-patch connectivity and normalized to the pooled celltypes PV, SST, VIP for reference (see Suppl. Mat. Sec. 8.1). Bars summarize n=1,000 Monte Carlo draws propagating binomial uncertainty in connection probabilities; error bars ±1 s.d. d) Inferred R_{motifs} for strengthened SST (yellow), PV (green) or VIP (red) populations. Same statistics as panel c.

7.2 Analysis of σ

Recall that the recurrency $R = \sigma \cdot R_{\mathrm{motifs}}$ has an overall scaling term, σ, and a motif contribution term R_{motifs} given by Eq. 3 of the main text. We began by computing σ, which is determined by the probabilities and strengths of individual connections within the mouse V1 network, and how these differ across layers and cell types. We computed the value of σ via the multi-population analysis introduced in [22] and detailed for our application in Supp. Mat. Sec. 8. To compute the relative σ, as displayed in Fig. S7b and Fig. S8c, we first computed a baseline value $\sigma_{baseline}$. In the layer-resolved analysis, this baseline corresponds to the full circuit of excitatory and inhibitory populations across layers 2 and 5. We then computed the relative decrease in σ upon limiting the analysis to the subcircuit of excitatory and inhibitory populations of a given layer (Fig. S7b). In the cell-type specific analysis, the baseline considered synapses of all inhibitory cell types to be equally strong. In this case we computed the relative increase or decrease in σ upon strengthening the efficacy of synapses of a given cell type (for simplicity by multiplying their strength by a factor 2, Fig. S8c), for details see Supp. Mat. Sec. 8).

The stronger connectivity of layer 2 vs layer 5 (Fig. S7a) results in a higher σ and suggests a larger R in layer 2 (Fig. S7b), consistent with the lower dimensionality found there (Fig. 3d right). The stronger connectivity of PV neurons vs SST and VIP cells (Fig. S8b) predicts that PV cells contribute stronger to σ and thus the recurrency of the overall circuit than other inhibitory cell types. This in turn predicts that changes in PV activity will therefore also yield greater changes in recurrency (Fig. S8c) and hence dimensionality.

7.3 Analysis of R_{motifs}

The above calculated σ contribution is not the only factor determining the recurrency. Therefore, we next analyzed the probability of occurrence of connection motifs, hence estimating the remaining contribution to recurrency R_{motifs} to test whether it predicts similar trends as the σ contribution.

For the comparisons across layers we again limited our analyses to the excitatory and inhibitory cells of a specific layer, and we asked whether cross-layer differences in connectivity motifs could likewise affect the recurrency, and hence dimensionality and its modulation across states.

We found that the corresponding motif contribution R_{motifs} for excitatory synapses was significantly stronger for layer 2 and 5 than for layer 6 (Fig. S7c right).

However, when combined with II and EI effects, the overall difference in R_{motifs} across layers 2 and 5 reversed (Fig. S7c left) for our default parameters. We conclude that we have evidence that differences in connection strength σ do contribute to R varying across layers, but in opposite effect to the differences

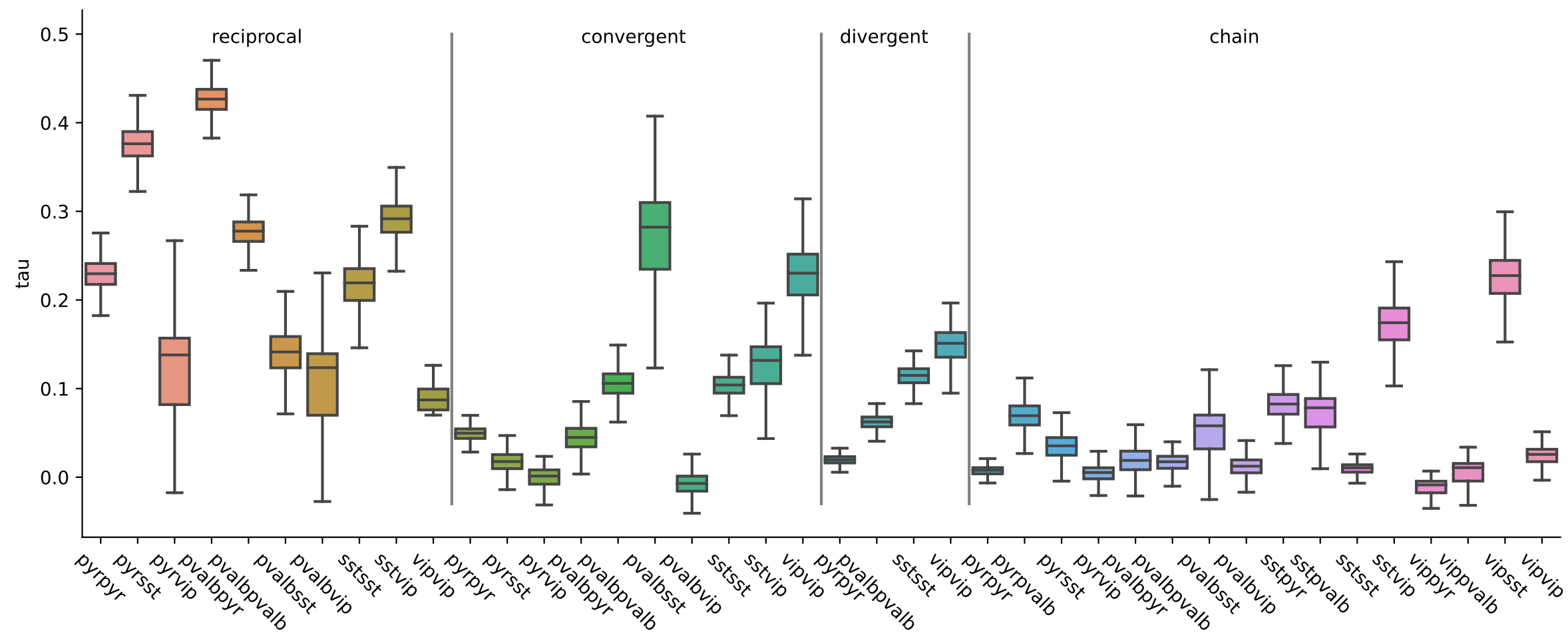

Figure S9: **Motifs of pyramidal neurons with different inhibitory cell types.** This figure shows that the abundance of reciprocal, convergent, divergent and chain motifs of pyramidal cells with different inhibitory cell types varies strongly in the synaptic physiology dataset, indicating that differential activation of inhibitory cell types can have differential impacts on the overall recurrency of the underlying network, as shown in Fig. S8d. Boxes show median and IQR of n=1000 bootstraps based on random subsets of 80% of sessions, whiskers show 1.5 IQR.

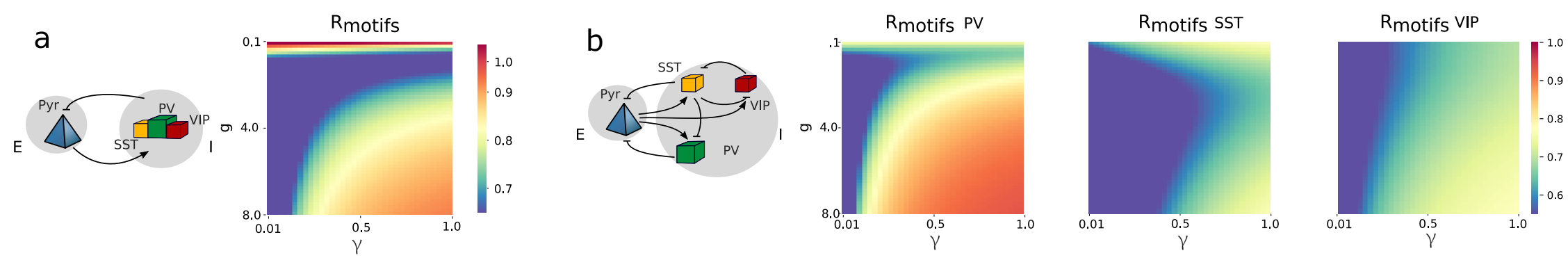

Figure S10: **Parameter scan over coupling strengths and population sizes.** This figure shows that our results on the reduction of recurrency by motifs hold for large ranges of inhibitory-to-excitatory coupling strengths g and relative population sizes γ. a) Inferred R_{motifs} as a function of relative strength g of inhibitory and excitatory synapses and ratio γ of inhibitory to excitatory population size. Unless stated otherwise, the reference values used in the analyses are $g = 4, \gamma = 0.25$. b) Same as a where PV (left), SST (center) or VIP (right) synapses have been strengthened to test the impact of these connections on R_{motifs}.

in synaptic motifs. Finally, we performed a similar analysis on the human synaptic dataset. While the more limited data allowed for fewer comparisons, this analysis showed that the values of R_{motifs} for excitatory connections in human cortex layers 2 and 3 are broadly consistent with those observed in layer 2/3 of the mouse cortex (Fig. S7d).

To test whether PV, SST and VIP activity modulation could alter recurrency by different engagement of their motifs in cortical circuits, we compared the values of the motif contribution to recurrency, R_{motifs}, for three distinct scenarios: when either the PV, the SST or the VIP is the dominant inhibitory population. For this analysis we strengthened synapses whose presynaptic site was of a given cell type by a factor 2.

We found that a circuit with a dominant PV inhibitory population had a substantially higher level of R_{motifs} compared to SST and VIP (Fig. S8d). This was true across all choices of parameters (Fig. S10b). In contrast to the case for layers, for cell type modulation, we thus find consistent roles for differences in both σ and R_{motifs} to induce changes in R and hence changes in dimensionality. In sum, our analyses show how cell-type specific control of interneural populations could lead to a global modulation of dimensionality via local circuit mechanisms.

8 Theory of σ in multi-population networks with statistics depending on pre- and post-synaptic population identities

The result from [22] on the spectral radius $R = \sigma$ of block-structured connectivity matrices is

$$\sigma = \sqrt{\max(\lambda(M))}\,, \tag{S46}$$

where $\lambda(M)$ are eigenvalues of the matrix M that contains the variances of each block of the connectivity and the size of the block:

$$M_{\alpha\beta} = \gamma_\beta \mathcal{G}^2_{\alpha\beta} = N_\beta \frac{\mathcal{G}^2_{\alpha\beta}}{N}\,. \tag{S47}$$

Here $\gamma_\beta = \frac{N_\beta}{N}$ is the fraction of all cells that belong to population β and $\frac{\mathcal{G}^2_{\alpha\beta}}{N}$ is the variance of connections in block (α, β). For Bernoulli connectivity, the variance is given by the connection probability $p_{\alpha\beta}$ and the synaptic strength $J_{\alpha\beta} = g_{\alpha\beta} J$ of connections from population β to population α:

$$\frac{\mathcal{G}^2_{\alpha\beta}}{N} = p_{\alpha\beta}(1 - p_{\alpha\beta}) g^2_{\alpha\beta} J^2\,. \tag{S48}$$

So in total one obtains

$$\begin{aligned} M_{\alpha\beta} &= NJ^2 \gamma_\beta p_{\alpha\beta}(1 - p_{\alpha\beta}) g^2_{\alpha\beta} \\ &= NJ^2 m_{\alpha\beta}\,, \end{aligned}$$

which has an overall scaling factor NJ^2 and a block-specific component $m_{\alpha\beta}$ that depends on population sizes, population specific connection probabilities and relative synaptic efficacy factors.

8.1 Relative recurrency

In Fig. S7b and Fig. S8c we compare the recurrency of a circuit with the recurrency of modified versions of that circuit. In case of the layer analysis (Fig. S7b), the circuit corresponds to 4 populations (2 excitatory, 2 inhibitory) spread across layers 2 and 5. The modified circuits are the subcircuits within either layer 2 or 5, respectively. For $X \in \{2, 5\}$, we obtain the relative recurrency as

$$\frac{\sigma_{LX}}{\sigma_{all}} = \sqrt{\frac{\max(\lambda(M_{LX}))}{\max(\lambda(M_{all}))}} = \sqrt{\frac{\max(\lambda(m_{LX}))}{\max(\lambda(m_{all}))}}$$

with

$$m_{all} = \begin{pmatrix} m_{L2E,L2E} & m_{L2E,L2I} & m_{L2E,L5E} & m_{L2E,L5I} \\ m_{L2I,L2E} & m_{L2I,L2I} & m_{L2I,L5E} & m_{L2I,L5I} \\ m_{L5E,L2E} & m_{L5E,L2I} & m_{L5E,L5E} & m_{L5E,L5I} \\ m_{L5I,L2E} & m_{L5I,L2I} & m_{L5I,L5E} & m_{L5I,L5I} \end{pmatrix} \tag{S49}$$

and

$$m_{L2} = \begin{pmatrix} m_{L2E,L2E} & m_{L2E,L2I} & 0 & 0 \\ m_{L2I,L2E} & m_{L2I,L2I} & 0 & 0 \\ 0 & 0 & 0 & 0 \\ 0 & 0 & 0 & 0 \end{pmatrix}, \quad m_{L5} = \begin{pmatrix} 0 & 0 & 0 & 0 \\ 0 & 0 & 0 & 0 \\ 0 & 0 & m_{L5E,L5E} & m_{L5E,L5I} \\ 0 & 0 & m_{L5I,L5E} & m_{L5I,L5I} \end{pmatrix}. \tag{S50}$$

In case of the cell-type specific analysis (Fig. S8c), the circuits consist of one pyramidal cell population and three inhibitory populations (PV, SST and VIP). The modified circuits model the case

where one of the inhibitory populations has an increased synaptic efficacy with respect to the others, e.g. $g_{\alpha,PV} = 2 \cdot g_{\alpha,SST} = 2 \cdot g_{\alpha,VIP}$ for the case of a PV dominated circuit. The relative recurrency of the modified circuit with strengthened population κ is then given by

$$\frac{\sigma_\kappa}{\sigma_{\mathrm{ref}}} = \sqrt{\frac{\max(\lambda(M_\kappa))}{\max(\lambda(M_{\mathrm{ref}}))}} = \sqrt{\frac{\max(\lambda(m_\kappa))}{\max(\lambda(m_{\mathrm{ref}}))}}\,,$$

where m_{ref} has the reference values $g_{\alpha,PV} = g_{\alpha,SST} = g_{\alpha,VIP}$ and m_κ has an increased $g_{\alpha,\kappa}$ by a factor 2.

Note that this analysis which takes into account pre- and postsynaptic identity of connections refines the analysis of σ in Sec. 4.3. Qualitatively, the two expressions for σ (pre-post vs pre-only), however, yield the same results for the layer and cell-type resolved analyses.

References

[1] Ginzburg I, Sompolinsky H. Theory of Correlations in Stochastic Neural Networks. Physical Review E. 1994;50(4):3171–3191. doi:10.1103/PhysRevE.50.3171.

[2] Tetzlaff T, Helias M, Einevoll GT, Diesmann M. Decorrelation of Neural-Network Activity by Inhibitory Feedback. Plos Computational Biology. 2012;8(8):e1002596. doi:10.1371/journal.pcbi.1002596.

[3] Pernice V, Staude B, Cardanobile S, Rotter S. How Structure Determines Correlations in Neuronal Networks. Plos Computational Biology. 2011;7(5):e1002059. doi:10.1371/journal.pcbi.1002059.

[4] Lindner B, Doiron B, Longtin A. Theory of oscillatory firing induced by spatially correlated noise and delayed inhibitory feedback. Physical Review E. 2005;72(6):061919. doi:10.1103/PhysRevE.72.061919.

[5] Trousdale J, Hu Y, Shea-Brown E, Josic K. Impact of Network Structure and Cellular Response on Spike Time Correlations. Plos Computational Biology. 2012;8(3):e1002408. doi:10.1371/journal.pcbi.1002408.

[6] Helias M, Tetzlaff T, Diesmann M. Echoes in correlated neural systems. New Journal of Physics. 2013;15:023002.

[7] Layer M, Helias M, Dahmen D. Effect of Synaptic Heterogeneity on Neuronal Coordination. PRX Life. 2024;2:013013. doi:10.1103/PRXLife.2.013013.

[8] Ricciardi L, Sacerdote L. Ornstein-Uhlenbeck Process as a Model for Neuronal-Activity .1. Mean and Variance of the Firing Time. Biological Cybernetics. 1979;35(1):1–9. doi:10.1007/BF01845839.

[9] Grytskyy D, Tetzlaff T, Diesmann M, Helias M. A unified view on weakly correlated recurrent networks. Frontiers in Computational Neuroscience. 2013;7:UNSP 131. doi:10.3389/fncom.2013.00131.

[10] Dahmen D, Grün S, Diesmann M, Helias M. Second type of criticality in the brain uncovers rich multiple-neuron dynamics. Proceedings of the National Academy of Sciences. 2019;116(26):13051–13060.

[11] Dahmen D, Layer M, Deutz L, Dabrowska PA, Voges N, von Papen M, et al. Global organization of neuronal activity only requires unstructured local connectivity. eLife. 2022;11:e68422.

[12] Williamson RC, Cowley BR, Litwin-Kumar A, Doiron B, Kohn A, Smith MA, et al. Scaling Properties of Dimensionality Reduction for Neural Populations and Network Models. PLOS Computational Biology. 2016;12(12):1–27. doi:10.1371/journal.pcbi.1005141.

[13] van Vreeswijk C, Sompolinsky H. Chaos in neuronal networks with balanced excitatory and inhibitory activity. Science (New York, NY). 1996;274(5293):1724–1726. doi:10.1126/science.274.5293.1724.

[14] Sommers HJ, Crisanti A, Sompolinsky H, Stein Y. Spectrum of large random asymmetric matrices. Physical Review Letters. 1988;60(19):1895–1898.

[15] Martí D, Brunel N, Ostojic S. Correlations between synapses in pairs of neurons slow down dynamics in randomly connected neural networks. Physical Review E. 2018;97(6):062314. doi:10.1103/PhysRevE.97.062314.

[16] Recanatesi S, Ocker GK, Buice MA, Shea-Brown E. Dimensionality in recurrent spiking networks: global trends in activity and local origins in connectivity. PLoS computational biology. 2019;15(7):e1006446.

[17] Brunel N. Dynamics of sparsely connected networks of excitatory and inhibitory spiking neurons. Journal of Computational Neuroscience. 2000;8(3):183–208. doi:10.1023/A:1008925309027.

[18] Hu Y, Brunton SL, Cain N, Mihalas S, Kutz JN, Shea-Brown E. Feedback through graph motifs relates structure and function in complex networks. Phys Rev E. 2018;98:062312. doi:10.1103/PhysRevE.98.062312.

[19] Zhao L, Beverlin B, Netoff T, Nykamp DQ. Synchronization from Second Order Network Connectivity Statistics. Frontiers in Computational Neuroscience. 2011;5. doi:10.3389/fncom.2011.00028.

[20] Song S, Sjostrom PJ, Reigl M, Nelson S, Chklovskii DB. Highly nonrandom features of synaptic connectivity in local cortical circuits. Plos Biology. 2005;3(3):507–519. doi:10.1371/journal.pbio.0030068.

[21] Campagnola L, Seeman SC, Chartrand T, Kim L, Hoggarth A, Gamlin C, et al. Local connectivity and synaptic dynamics in mouse and human neocortex. Science. 2022;375(6585):eabj5861. doi:10.1126/science.abj5861.

[22] Aljadeff J, Stern M, Sharpee T. Transition to Chaos in Random Networks with Cell-Type-Specific Connectivity. Physical Review Letters. 2015;114:088101. doi:10.1103/PhysRevLett.114.088101.
